# Ecological Sustainability Assessment of Water Distribution for the Maintenance of Ecosystems, their Services and Biodiversity

**DOI:** 10.1007/s00267-022-01662-3

**Published:** 2022-06-14

**Authors:** Anna Schlattmann, Felix Neuendorf, Kremena Burkhard, Elisabeth Probst, Estanislao Pujades, Wolfram Mauser, Sabine Attinger, Christina von Haaren

**Affiliations:** 1 Institute of Environmental Planning, Leibniz University Hannover, Herrenhaeuserstr. 2, 30419 Hannover, Germany; 2 Department of Geography, Ludwig-Maximilians-Universität München, Luisenstr. 37, 80333 München, Germany; 3 Department of Computational Hydrosystems, Helmholtz Centre for Environmental Research - UFZ, Permoserstr. 15, 04318 Leipzig, Germany

**Keywords:** Sustainability assessment, Water distribution, Biodiversity, SDGs, Ecosystem Services, GIS

## Abstract

Water provision and distribution are subject to conflicts between users worldwide, with agriculture as a major driver of discords. Water sensitive ecosystems and their services are often impaired by man-made water shortage. Nevertheless, they are not sufficiently included in sustainability or risk assessments and neglected when it comes to distribution of available water resources. The herein presented contribution to the Sustainable Development Goals Clean Water and Sanitation (SDG 6) and Life on Land (SDG 15) is the Ecological Sustainability Assessment of Water distribution (ESAW-tool). The ESAW-tool introduces a watershed sustainability assessment that evaluates the sustainability of the water supply-demand ratio on basin level, where domestic water use and the water requirements of ecosystems are considered as most important water users. An ecological risk assessment estimates potential impacts of agricultural depletion of renewable water resources on (ground)water-dependent ecosystems. The ESAW-tool works in standard GIS applications and is applicable in basins worldwide with a set of broadly available input data. The ESAW-tool is tested in the Danube river basin through combination of high-resolution hydro-agroecological model data (hydrological land surface process model PROMET and groundwater model OpenGeoSys) and further freely available data (water use, biodiversity and wetlands maps). Based on the results, measures for more sustainable water management can be deduced, such as increase of rainfed agriculture near vulnerable ecosystems or change of certain crops. The tool can support decision making of authorities from local to national level as well as private enterprises who want to improve the sustainability of their supply chains.

## Introduction

Driven by climate change and growing user demands, water is becoming an increasingly contested resource (Bos et al. 2009). This applies not only to the naturally dry regions but to most of the populated parts of the world. A major pressure in many river basins is the alteration of water regimes caused by increased agricultural water use (Falkenmark and Rockström 2006; United Nations 2018). Unsustainable water use is often correlated with the loss of ecosystem services such as drinking water provision, regulating services, habitat function and biodiversity (Foley et al. 2015), particularly in wetlands (Grizzetti et al. 2016; Russi et al. 2013; UN Water 2020). The driving forces behind unsustainable water use are manifold. A common denominator seems to be, that regulation of water use very often has a problem of spatial fit (Moss and Newig 2010), meaning that the multiscale management according to the motto “think of the basin act local” is not sufficiently implemented and decisions are taken on levels which cannot take responsibility for the impacts on higher scales. Water availability on watershed level needs also to set the limits of water use for subordinate spatial entities.

If limits for water use are not communicated including their legitimate value basis (van Oudenhoven et al. 2018; Heink and Kowarik 2010; Hagan and Whitman 2006) and are not implemented, many unconnected small decisions on local scale lead in their summative effect to regional overuse. Legitimate but abstract norms for water use limits are provided by international conventions and treaties (Schlattmann et al. 2021). Even in countries with a systematic water management such as in Europe, the normative principles of local distribution are seldom transparent and in times of droughts local use extends the boundaries of what is available in the catchment areas—a problem which is increasing with climate change.

Furthermore, very often actual water allocation neglects international standards of biodiversity protection as well as resulting consequences such as hydrological buffer zones around water sensitive ecosystems. A reason may be the lack of integrative environmental and spatial planning (Rijsberman and Molden 2001; Cooper and Hiscock 2019) which might be solved by the application of sustainability assessments. Their added value is given when their indicators account for intra-annual variations of water supply and demand on basis of monthly time steps (Vanham et al. 2018) and the assessment is spatially specific (Albert et al. 2016; Grizzetti et al. 2016).

In essence, the present challenge to manage sustainable water use lies in spatial modelling of limits and priorities of water distribution and use expressed in abstract legal norms—including the needs of water sensitive ecosystems—at spatial scales reaching from local to watershed level. The design of water sustainability assessments should include the demands of implementation by considering that responsibilities are allocated to different political levels as well as the demand to deduce concrete measures on local scale from the spatially explicit results.

Existing tools for water scarcity assessment provide a baseline for further methodological progress to respond to these challenges. They range from simple depletion ratios and single indicators such as the Water Stress Indicator (Smakhtin et al. 2004a) to more elaborate assessment tools with multiple indicators i.e. Watershed Sustainability Index (Chaves and Alipaz 2007) or Water Footprint (Hoekstra et al. 2011). A common, well-accepted indicator is the minimum stream flow (FAO 2018), because of its key role for maintaining habitats and biodiversity a prerequisite for further ecosystem services provision (Wallace et al. 2003; Pastor et al. 2014). Green and green-blue water assessments (Schyns et al. 2015) additionally include evapotranspiration as environmental water requirement (Rockström and Gordon 2001; Fisher et al. 2017). However, until now sustainability assessments do not systematically consider the legitimacy of their sustainability indicators, leading to a weak normative basis for prioritization of rights to water. Furthermore, mostly they do not cover multiple scales or do not lead to area specific results about the impairment of ecosystem functioning and biodiversity (Smakhtin et al. 2004b; Vanham et al. 2018).

This study attempts to overcome these limitations by developing a sustainability assessment approach that builds on existing methods but reacts to the challenges of:

- referencing to legitimate international sustainability standards
- a focus on biodiversity and related ecosystem services and thus on ecological integrity of water use assessments
- multiscale application reacting to the interrelations between different decision levels and water-governance tasks
- suitability for broad practical implementation in water management and spatial planning

**Fig. 1:**
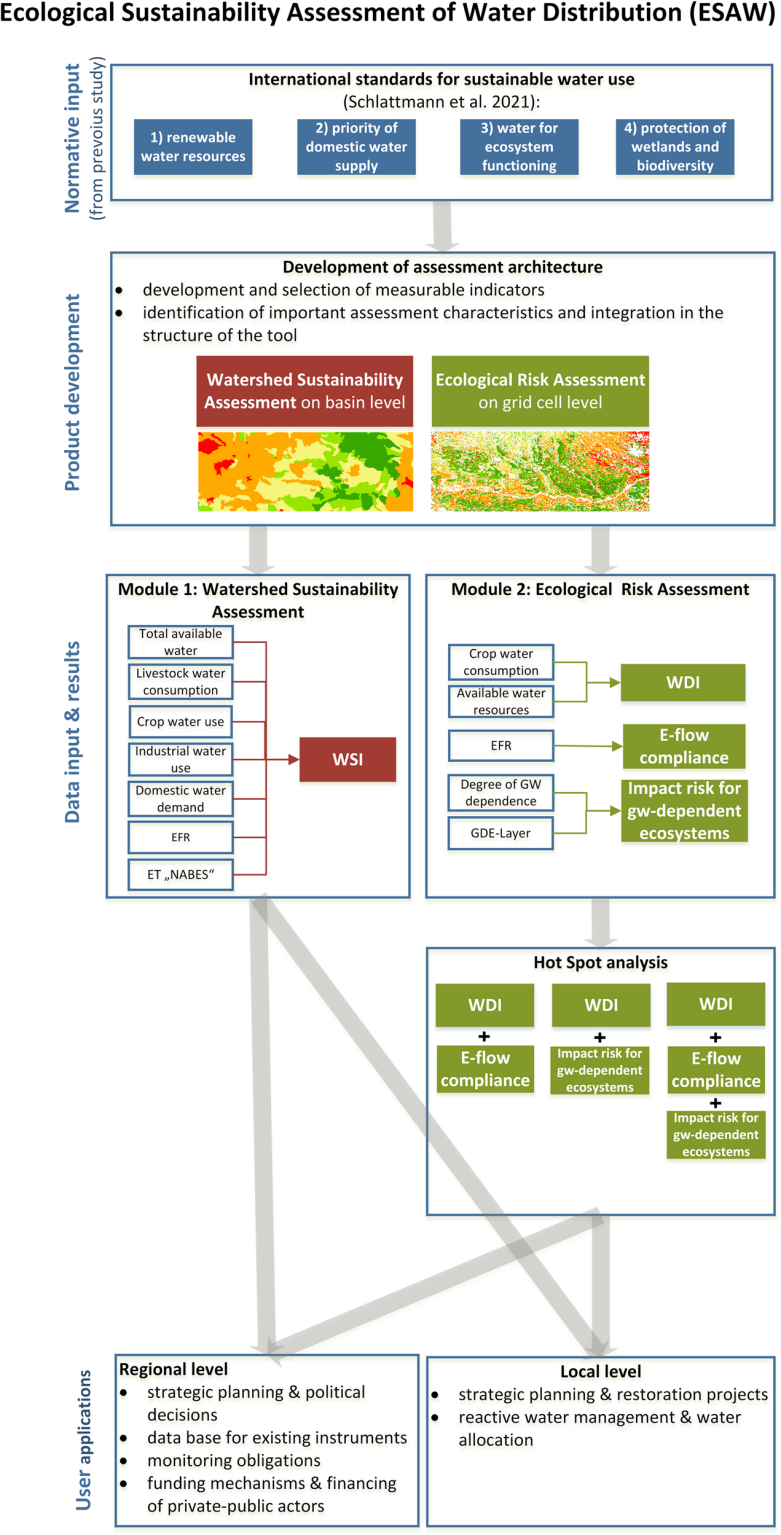
Workflow of the development and application of the multiscale ESAW-tool including the main data inputs, processes, outcomes, and user applications

The herein presented Ecological Sustainability Assessment of Water distribution (ESAW-tool) is a watershed sustainability tool with an ecological risk assessment. The watershed sustainability is assessed by a single indicator. The ecological risk assessment is implemented through three indicators on finer grid resolution (Fig. 1). Ecosystems and related water allocation issues often transgress national boundaries. To overcome normative issues across boundaries, the assessment applies a globally equal basis for sound management of water resources (Schlattmann et al. 2021) and evidence-based decision-making (Opdam et al. 2002; von Haaren et al. 2008). Sustainability, in this study is inspired by the definition given in the Brundtland Report (1987): “Sustainable development is development that meets the needs of the present without compromising the ability of future generations to meet their own needs”. The authors appreciate that sustainability is inseparable composed of economy, society, and ecology. However, multi-dimensional sustainability assessments directly based on Brundtland or the SDGs, have inherent conflicts of priorities between the objectives. Those are considered solvable on the implementation level, which often is not the case. The authors pay tribute to this problem and base their assessment on the two SDGs: Clean Water and Sanitation (SDG 6) and Life on Land (SDG 15) (UN 2015) with clear priorities between the water-related sustainability objectives. This focus enables place-based implementation of the targets concerning ecosystem health. Thus, the developed ESAW-tool reconciles the evaluation of ecosystem related targets for sustainable water use, as the combination of all sustainability dimensions would not be beneficial in a standardized assessment. The inter-generation aspiration for sustainability can be addressed by applying the tool for long-term periods.

The ESAW-tool is tested in the Danube basin for the vegetation periods 2015–2018 with high resolution hydro-agroecological and groundwater model data and further freely available data to explore sensitivity and adaptive capacity in a representative and large river basin with diversity of natural landscape conditions. Finally, the authors discuss possibilities of application in decision-making on multiple levels.

## Materials and Methods

The ESAW-tool is developed as application for standard geoinformation systems (GIS) that can be applied in river basins worldwide with a set of hydrological, water use and land cover variables. Freely available databases are applicable in the ESAW-tool. The present study is conducted with data from hydro-agroecological model PROMET (Processes of Mass and Energy Transfer) for surface water and agricultural variables and the hydrogeological model OpenGeoSys for groundwater variables. The dataset is supplemented with freely available data, including biodiversity maps, industrial and domestic water use, and livestock water consumption. Results are computed monthly in a GIS in WGS 84 coordinate system.

### Case Study

**Fig. 2:**
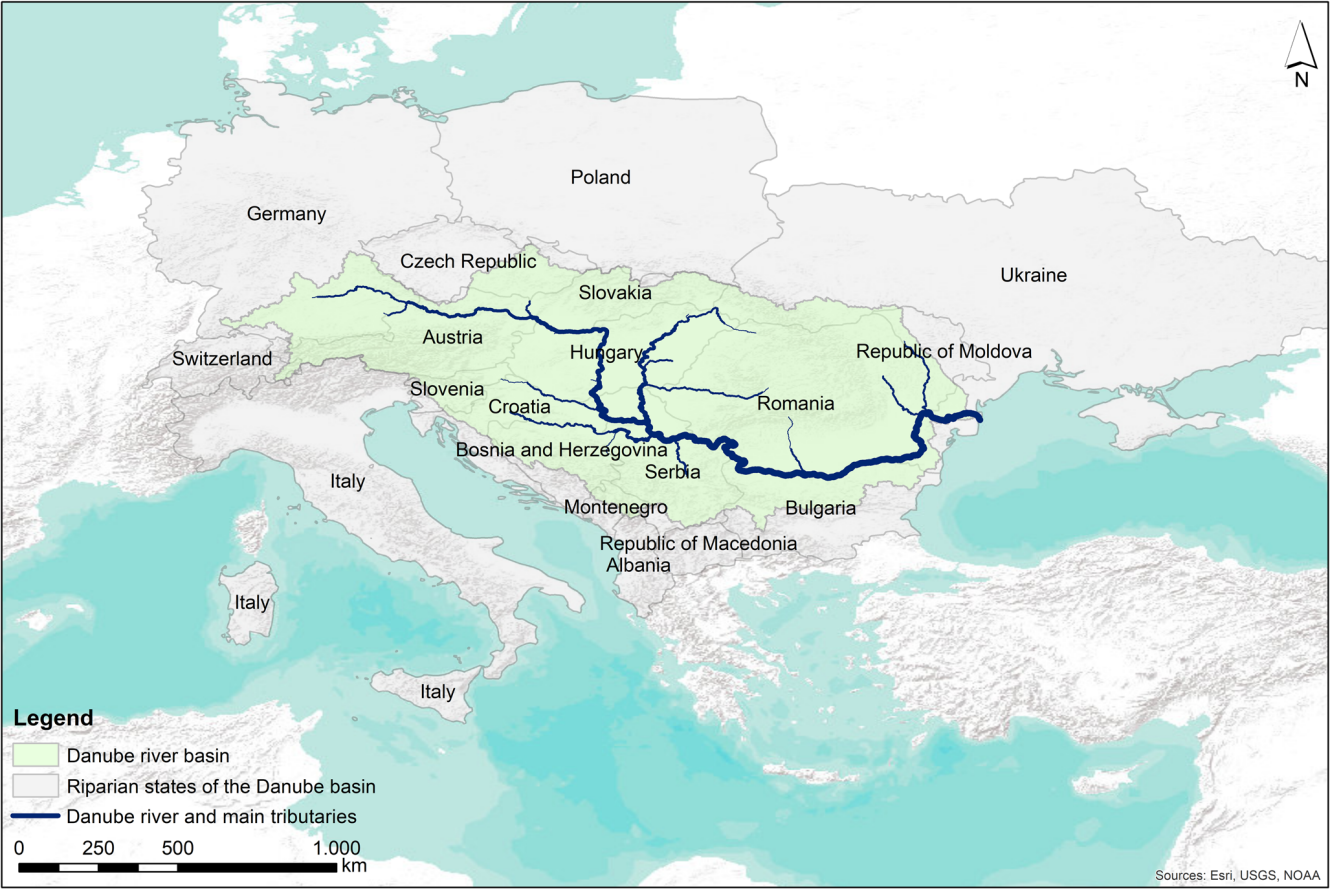
Location of the Danube river basin in Europe and riparian states of the Danube basin (Based on: ESRI World terrain Base; TM_WORLD_BORDERS-0.1, Lehner and Grill 2013)

The Danube river is 2857 km long and its basin area is 801,463 km², which makes it Europe’s second largest river basin (ICDPR 2009). The basin covers parts of 19 countries in Western, Central and Eastern Europe (Fig. 2), being the most international river basin in the world (ICDPR 2009). The Danube basin constitutes ideal conditions to test the ESAW-tool in a representative and large river basin with potential conflicts between socio-economic and environmental water demands.

### Mechanistic Hydro-Agroecological Model PROMET

The key variables related to the water balance in the Danube basin including river discharge and vegetation water use, are obtained from simulations with the mechanistic hydro-agroecological model PROMET (Mauser and Bach 2009; Mauser et al. 2015). PROMET simulates water and carbon fluxes under a closed mass and energy balance, calculating dynamic plant growth and river discharge in the Danube basin on hourly time steps with a spatial resolution of ~1 km² (0.00833333°). Within PROMET, dynamic plant growth is calculated with a biophysically based vegetation module (Hank et al. 2015) while river discharge is simulated using the Muskingum-Cunge-Todini approach (Cunge 1969; Todini 2007). Simulations are performed with a validated PROMET setup (Probst and Mauser 2022) using the ERA5-GPW meteorological forcing dataset. Here, ERA5 reanalysis data (Hersbach et al. 2020) were downscaled to 0.00833333° spatial resolution and bias-corrected with a combination of the global WorldClim2 temperature and precipitation climatologies (Fick and Hijmans 2017) and the Alpine precipitation climatologies GLOWA (Früh et al. 2006) and PRISM (Frei and Schär 1998).

### Hydrogeological Numerical Model—OpenGeoSys

Groundwater data is obtained from a hydrogeological numerical model developed with the code OpenGeoSys (Kolditz et al. 2012). The numerical model covers the whole Danube basin with a resolution of 500 m and allows computing the dynamics of shallow groundwater systems. It is a 2D model in which the aquifer thickness is accounted through the aquifer’s transmissivity, whose distribution is derived by analysing the spectral signal of the river baseflow measured at multiple gauging stations (Pujades et al. 2020; Di Dato et al. 2020). The values for storage coefficient were obtained from the GLobal Hydrology MaPs 2.0 (GLHYMPS 2.0) proposed by Gleeson et al. (2011). Groundwater elevation data is used to estimate groundwater dependence of ecosystems.

### Valuation Basis from Legitimate International Sustainability Standards

The following standards are used for the assessment: (1) Water use is restricted to renewable resources, where availability is estimated based on precipitation and water use must not exceed this amount; (2) Priority of domestic water supply over other uses defines that the realization of recommended minimum supply for domestic use is condition for sustainable water use patterns; (3) Water provision for ecosystem functioning defines the minimum amount of stream flows and evapotranspiration needed to maintain long-term functioning of ecosystems (and their service provision); (4) Protection of wetlands and biodiversity through adequate water supply comprises the identification of water sensitive ecosystems, their vulnerability and potential threats for their water supply.

These standards are considered as legitimate standards as they are declared and prioritized by international norms for sustainable water use (Schlattmann et al. 2021) and represent the minimum internationally accepted requirements of sustainable water use. Thus, they can guarantee for an unbiased transparent evaluation by governments and water related authorities.

The standards differ in their degree of specificity and are not directly measurable. In this study the standards were translated into measurable indicators using existing interpretations of the laws, legal specifications and already proposed indicators. The detailed workflow can be found in Online Resource 1. The development of the indicators is very much based on the ecosystem approach. In this study, an ecosystem is understood, similar to “habitat” or “biotope”, as the totality of abiotic and biotic characteristics, including typical species diversity, which together provide typical ecosystem related services.

### Methodological Framework

The ESAW-tool is designed in two modules to overcome restricted possibilities for quantification of some of the aforementioned standards and to enable a multi-scale assessment on sub-basin level and with finer grid resolution (Fig. 1):

1. Watershed sustainability assessment: a spatial sustainability evaluation of a calculated supply-demand ratio on sub-basin level. Demand is quantified according to actual water consumption. The legitimate international sustainability standards determine the minimum amount of water that needs to be allocated for domestic use and the water requirements of healthy ecosystems as *“priority water uses”*. Supply is determined through renewable resources availability. Conflicts where demand exceeds supply are identified and quantified as unsustainable water over-use. The degree of sustainability is expressed in one single indicator, the “water sustainability index (WSI).
2. Ecological Risk Assessment: a differentiating module, emphasizing the site-specific ecological risk for water-dependent biodiversity and ecosystem services with 1 × 1 km grid cell resolution. This ecological risk assessment is based on three indicators: water depletion index (WDI), environmental flow requirements (e-flows) and the impact risk for groundwater-dependent ecosystems. For the last indicator, quantification of water (over-)use is not possible due to vague normative context or limited availability of detailed data about water use on local scale. The results of (2) are interpreted for deducing local measures by using the calculated and spatially specific boundaries of sustainable water availability from component (1).

#### Watershed sustainability assessment

The watershed sustainability is assessed through the water sustainability index (WSI) that shows the degree of exploitation of sustainably usable water resources. It evaluates the supply-demand ratio on sub-basin level (HydroSHEDS level 8, Lehner and Grill 2013) through using the method of the *Water Stress Indicator* (Smakhtin et al. 2004b), expanded by the inclusion of sustainability requirements, that prioritise a minimum domestic water demand and the water requirements for sustaining ecosystem functioning (Schlattmann et al. 2021). The sustainable consumption capacity for other users, primarily industry and agriculture, is defined as the difference of total available water resources and the water that should be reserved for *“priority water uses”*. The degree of (un)sustainability of water use is calculated as WSI from water consumption of agricultural and industrial purposes, and the difference of total available water and water for priority uses (Eq. 1). The priority water uses (W_prio_) are based on World Health Organization (WHO) estimates for minimum domestic water demand (DWD), ‘environmental flow requirements’ (e-flows) and evapotranspiration of natural and semi-natural ecosystems (ET_nat_) (Eq. 2). Water consumption is considered as net consumption as for agriculture, net water consumption can be computed more reliably than gross consumption. The level of exploitation defined by the WSI are described in Table 1.

1 $$WSI = \frac{{ETagri + Wliv + Wind}}{{WAt - Wprio}}$$

2 $$W_{prio} = DWD + e - flows + ETnat$$

Note: WSI = Water Sustainability Index; ET_agri_ = agricultural water use for crop growth; W_liv_ = agricultural water use for livestock production; W_ind_ = industrial water consumption; WA_t_ = total available water; W_prio_ = priority water use; DWD = domestic water demand; e-flows = environmental flow requirements; ET_nat_ = evapotranspiration natural and semi-natural ecosystems

**Table 1:** Classification of WSI in five classes depending on the degree of exploitation of sustainably usable water resources, adopted from (Smakhtin et al. 2004a)

WSI	Degree of exploitation of sustainably usable water resources
WSI < 0	Extremely overexploited (monthly sustainably usable water is lower than sum of priority uses, fossil water or surplus of preceding months is used)
WSI ≥ 1	Overexploited (current water exploitation is higher than allowed sustainable levels)
0.6 ≤ WSI < 1	Heavily exploited (0 to 40% of sustainably usable water is still available in a sub-basin)
0.3 ≤ WSI < 0.6	Moderately exploited (40% to 70% of the sustainably usable water is still available in a sub-basin)
0 ≤ WSI < 0.3	Slightly exploited (70% or more of the sustainably usable water is still available in a sub-basin)

##### Renewable water and total available water

In this study, renewable water resources are defined as the water that is replenished by the hydrological cycle within monthly time steps. The main sources of renewable water are soil moisture and flows including groundwater recharge that are generated through local precipitation and inflows from upstream basins (Shiklomanov 1998). Thus, according to Gassert et al. (2015), the total available water of a sub-basin (WA_t_) is computed as sum of internal renewable water resources (WR_t_) and the outflow from upstream sub-basins (Q_out_) into basin *i* after upstream water demand has been subtracted (Eqs. 3–6). Some “losses” from the short-term hydrological cycle, such as deep aquifer recharge are not considered:

3 $$WA_t\left( i \right) = WR_t\left( i \right) + {\sum} {Qout\,\left( {iup} \right)}$$

where WR_t_*(i)* is precipitation

4 $$WR_t\left( i \right) = P$$

and Q_out_ is

5 $$Q_{out}\left( i \right) = {{{\mathrm{max}}}}\left( {0,\,WA_t\left( i \right) - WD\left( i \right)} \right)$$

where WD is the water demand from different users:

6 $$\begin{array}{l}WD\left( i \right) = W_{liv}\left( i \right) + ET_{agri} + W_{ind}\left( i \right)\\ \qquad\quad\quad\;\; +\, DWD\left( i \right) + e - flows\left( i \right) + ET_{nat}\left( i \right)\end{array}$$

Note: WA_t_ = total available water; WRt = internal renewable water; Q_up_ = outflow from upstream basins into basin; P = precipitation; Q_out_ = out flow from the basin; WD = water demand; W_liv_ = water for livestock; ET_agri_ = evapotranspiration in agriculture; DWD = domestic water demand; e-flows = Environmental Flow requirements; ET_nat_ = evapotranspiration of natural and semi-natural ecosystems

Negative values of Q_out_ were set to 0 since no sustainably usable water exits the sub-basin. In first order sub-basins Q_out_*(i*_*up*_*)* = 0, there, the total available water is equal to internal renewable water.

##### Domestic water demand

The Domestic Water Demand is computed as multiplication of the national values for water withdrawals per capita from 2008–2016 (FAO 2019b) and gridded population densities (Global Human Settlement Layer (GHSL), (Schiavina et al. 2019). The recommended values for minimum domestic water supply based on WHO recommendations (Schlattmann et al. 2021) are applied instead of the statistical values in the case that the statistical values are lower. The domestic water demand is assumed constant over the year. Return-flow ratios (Wada et al. 2011a) were applied to estimate the net water consumption.

##### Environmental flow requirements for rivers on basin scale

Environmental flow requirements (e-flows) estimate the share of the original flow regime of a river that should be preserved to maintain ecosystem functioning (King et al. 2008; Wallace et al. 2003). In this study the calculation of e-flows was based on the quantitative flow approach from Tessmann (1980) that considers intra-annual variation of flows. According to Tessmann, each month is classified as one of three types of hydrological months that are defined by the ratio of mean monthly flow (MMF) to mean annual flow (MAF) (Table 2).

**Table 2:** Rules for determination of low-, intermediate and high-flow months and recommended minimum flow, based on Tessmann (1980)

Type of hydrological month	Rule	Recommended monthly flow
Low-flow-month	MMF_long-term_ ≤ 40% of MAF_long-term_	100% of MMF_long-term_
Intermediate-flow-month	MMF_long-term_ > 40% of MAF_long-term_ & 40% of MMF_long-term_ ≤ 40% of MAF_long-term_	40% of MAF_long-term_
High-flow-month	MMF_long-term_ > 40% of MAF_long-term_ & 40% of MMF_long-term_ > 40% of MAF_long-term_	40% of MMF_long-term_

The type of hydrological month is estimated based on long-term MAF and long-term MMF of the period 1985–2015. Then, the recommended monthly flow is calculated as percentage of the modelled flow for each month of the observed vegetation periods, accordingly.

Herein, different from past approaches which used data from gauges, modelled discharge data from the PROMET is used. The minimum flow for each sub-basin is determined through modelled flow at its discharge point (Table 3).

**Table 3:** Classification of the WDI values into six classes depending on the degree of water resources exploitation

WDI	Degree of exploitation of water resources generated in grid cell
WDI < 0 (neg.)	Local available water is < 0, meaning that cell outflow is higher than monthly water input
WDI > 1.5	Crop water exploitation is significantly higher than available water resources
1 < WDI ≤ 1.5	Crop water exploitation is slightly to considerably higher than available water resources
0.7 < WDI ≤ 1	Almost full crop water exploitation of available water resources
0.3 < WDI ≤ 0.7	Intermediate crop water exploitation, recharge and storage of soil moisture possible
0 < WDI ≤ 0.3	Low crop water exploitation, recharge and storage of soil moisture possible

#### Evapotranspiration of natural and semi-natural ecosystems, important for biodiversity and ecosystem service provision

Natural and semi-natural ecosystems are defined as areas that are not urbanised, industrialised and not agriculturally managed, i.e. forestry (Online Resource 1). For the calculation of green water requirements to maintain functioning of these ecosystems the modelled evapotranspiration (ET_nat_) is summed up for each sub-basin.

##### Agricultural water consumption

The agricultural water consumption is composed of crop water consumption and livestock water demand. The calculation of crop water consumption (ET_agri_) is implemented analogous to the evapotranspiration of natural and semi-natural ecosystems (Online Resource 1).

Livestock water demand (W_liv_) is calculated according to the approach of Wada et al. (2011b). Livestock gross water demand is equated to net water demand. The gridded global livestock density for the most frequent livestock (Online Resource 1) are multiplied with breed specific water demands that are based on temperature and service water requirements (Steinfeld et al. 2006; Luke 1987; SCARM 2003). Water demand is averaged for different physiological conditions of the livestock types and production systems (Online Resource 1).

##### Industrial water consumption

The industrial water consumption is derived from country withdrawals (FAO 2019a). The spatially disaggregated industrial water consumption is computed assuming that the share of a countries’ area within the Danube basin is equal to the share of industrial water consumption in the basin. The amount of industrial water withdrawn in the Danube basin was then allocated to the industrial areas of the country within the Danube basin (Online Resource 1). Net water consumption was computed using return-flow ratios (see “Environmental flow requirements for rivers on basin scale”).

#### Ecological risk assessment

The relationship between agricultural water use and the impairment of ecosystems is evaluated following the concept of the ecological risk assessment (Bierhals et al. 1974), which is a common methodology applied in practical landscape planning (von Haaren 2004). The herein presented ecological risk assessment estimates the risk that agricultural water consumption in a particular area adversely impacts on biodiversity and further water-related ecosystem services. This assessment is composed of three indicators: (1) Water depletion in agricultural areas; (2) Environmental flow requirements of rivers; and (3) Risk for groundwater dependent ecosystems. The ecological risk assessment is computed with 0,00833333° (approx. 1 × 1 km) grid resolution. The results are rated on ordinal scales.

##### Water depletion in agricultural areas

Water depletion in agricultural areas is assessed through the water depletion index (WDI) that describes the degree to which crop growth exploits the available renewable water resources in five classes (Table 3). The WDI is calculated as ratio of crop water consumption (WC_agri_) and available renewable water (WA) (Eq. 7) within the agricultural area based on PROMET land cover distribution:

7 $$WDI = \frac{{WCagri}}{{WA}}$$

The crop water consumption is estimated as sum of modelled crop transpiration (rain fed and irrigated) and interception (Eq. 8), since both are strongly crop specific (Dunkerley and Booth 1999). The available renewable water resource is precipitation plus total inflow to the grid cell minus the total outflow of the grid cell minus (unproductive) soil evaporation including depression storage evaporation (E_soil_) (Eqs. 9–11):

8 $$WCagri = Tagri + Ei$$

9 $$WA = P + Qin - Qout - Esoil$$

where,

10 $$Qout = total\,discharge$$

and,

11 $$Qin = Qout\left( {up} \right)$$

Note: WDI = Water depletion index; WC_agri_ = agricultural water consumption for crop growth; WA = available renewable water resources on agricultural areas; T_agri_ = crop transpiration; E_i_ = interception evaporation; P = precipitation; Q_in_ = inflow to grid cell; Q_out_ = outflow from grid cell; E_soil_ = soil evaporation including depression storage evaporation.

The hydro-agroecological variables were calculated with PROMET. The inflow to the grid cells is derived from total discharge (routed) and flow direction information via cell IDs (Online Resource 1).

##### Compliance with environmental flow requirements

E-flows serve as indicator for the health of aquatic ecosystems and their connected wetlands. The gridded e-flows were computed as described in section “Environmental flow requirements for rivers on basin scale” and considering the presence of a”flushing period” with flow higher than 200% MAF as proposed by Tennant (1975) and Tessmann (1979) (Online Resource 1). The compliance of the river discharge with e-flows is implemented as difference between modelled discharge for the studied periods and required-flows. For the evaluation, the discharge values were ranked in five classes (Table 4).

**Table 4:** Classification of the degree of river flow compliance with e-flows in five classes depending on the degree of compliance with e-flows

Discharge (Q) surplus or shortage to recommended e-flows	Degrees of compliance with e-flows
Q ≥ e-flow and Q of one month of the year is ≥200% MAF	e-flow fully met—healthy river conditions that can be regarded as near natural
Q ≥ e-flow	Monthly e-flow met—good status of river flow, but functions depending on seasonal extreme events are not met
0.7 e-flow < Q < e-flow	e-flow slightly deteriorated
0.4 e-flow < Q ≤ 0.7 e-flow	Flow moderately unsustainable
Q ≤ 0.4 e-flow	Flow extremely unsustainable

##### Risk for groundwater-dependent ecosystems

Progressive groundwater pumping creates a cone of depression around the well which can potentially dry out groundwater dependent ecosystems such as rivers, springs and streams (Hiscock et al. 2002; Hiscock and Bense 2014). Shape and growth of the cone depend on the pumping rate and hydraulic properties of the aquifer (Hiscock and Bense 2014). The effect of groundwater drawdown on ecosystem well-being depends on their degree of groundwater dependency. Thus, even low groundwater decline of few metres or centimetres and small spread over few (hundred) metres close to rivers or wetlands can have detrimental impacts on shallow rooting ecosystems with high groundwater dependency (Rushton 2002).

The evaluation of the impact of agricultural groundwater consumption on groundwater-dependent ecosystems (GDEs) is implemented in two consecutive steps: (1) inventory analysis of GDEs, (2) evaluation of site-specific risks. The endangerment of a site and its sensitivity to changes in the groundwater regime determine the site-specific risk. The spatial relationship between agricultural water consumption and GDEs is implemented through buffer zones around the endangered GDEs. Within the buffer zones agricultural areas have a considerable impact risk.

#### Inventory analysis of GDEs

The identification of GDEs is carried out in two steps: (1) identification and mapping of potentially water-dependent ecosystem types through review of literature and relevant geodata; (2) analysis of the pre-selected areas regarding their actual dependence on groundwater, based on site specific characteristics.

Global and European ecosystem and habitat classifications were used as a basis for the mapping task. A thorough literature analysis (EEA 2019; Reich et al. 2012; BfN 2006; EC 2013) provided information about the potential water dependency of the ecosystem types. This information is included in an iterative process (Online Resource 1, Online Resource 2) that resulted in a global typology of ecosystem water dependency that can be linked to European habitat types (Online Resource 3) and a map of water dependent ecosystems for the Danube basin (Online Resource 4). All sites with ecosystem types that potentially rely on additional water resources than precipitation define the scope for the following analysis of actual groundwater-dependence.

In this study, GDEs are defined as “ecosystems that require access to groundwater to meet all or some of their water requirements on a permanent or intermittent basis, so as to maintain their communities of plants and animals, ecosystem processes and ecosystem services” (Richardson et al. 2011). From the different types of GDEs, this study considers wetlands and terrestrial ecosystems. The authors applied an inferential approach (Eamus et al. 2006) where, depth to groundwater and the water access of plant roots from the saturated zone are considered as decisive attributes. Monthly groundwater connection is identified either through (1) hydrologically active roots that reach the capillary fringe (GW_dep1_; Eqs. 12) or (2) through shallow groundwater (GW_dep2_; Eq. 15). For (1), the depth of hydrologically active roots (Fan et al. 2017) and the height of the capillary rise were subtracted from the depth to the groundwater table (Pujades et al. 2020; SRTM) (Eq. 12). The capillary rise is estimated through an empirical formula that is based on soil pore diameters (Rowell 1994) (Eq. 13). Pore diameter is deduced from a simplified relationship between particle size and pore diameters (ibid.) (Eq. 14). A detailed description is given in Online Resource 1. A site is identified as GDE if groundwater connection is given in at least one month of the vegetation period. The use of shallow expressions of groundwater is in the ESAW-tool identified when the mean monthly depth to the groundwater table of the period 2015–2018 is ≤100 cm during at least one month of the vegetation period (Eq. 15).

12 $$GW_{dep1} = GW_d - R_d - C_h \le 0$$

13 $$C_h = 3000/d$$

14 $$d = K/5$$

15 $$GW_{dep2} = GW_d\left( {MM_{veg2015 - 2018}} \right) \le 100\,cm\,for\,min.\,1\,month$$

Note: GW_dep_ = groundwater dependent; GW_d_ = depth to groundwater table; R_d_ = average maximum plant rooting depth; C_h_ = height of capillary rise [cm]; d = diameter of soil pores [µm]; K = diameter of particles [µm]

#### Evaluation of site-specific risks

The evaluation of site-specific risks for GDEs considers proven criteria of landscape planning (Kirsch-Stracke and Reich 2004), being: endangerment, rareness, and irreplaceability. For this study they were aggregated to the criteria *‘endangerment of site’* and *‘sensitivity of site’*. The endangerment of a site is identified by its conservation status according to global systems of protected areas and endangered sites. These are the IUCN protected areas (Dudley 2008), the RAMSAR protected sites (UNESCO 1971) and the Key Biodiversity Areas (KBA), Important Bird Areas (IBA) and Areas of Zero Extinction (AZE) (BirdLife International and Conservation International 2018). A high conservation status implies a high endangerment of the site (Table 5).

**Table 5:** Classification of the endangerment of the site based on the conservation status

Conservation status	Endangerment of site
● IUCN cat. 1a, 1b, 4 ● RAMSAR	Very high
● IUCN cat. 2	High
● IUCN cat. 5, 6, (3) ● IBA, AZE, KBA	Moderate
● None	Low

The sensitivity of a site to changes in the water regime is assessed through the risk of groundwater drawdown caused by pumping and the site’s degree of groundwater-dependence.

To estimate the risk of groundwater table decline, the authors consider aquifers with high productivity to have high transmissivities and specific yields. Meaning more water can be extracted per unit of decline in the water table (Hiscock and Bense 2014). The risk for significant changes in the water table caused by pumping is assumed inversely to aquifer productivity (Table 6). The aquifers classified as “Moderate and Low” productive (International Hydrogeological Map of Europe (IHME v1.2)) were divided into “Low” and “Moderate” productivity considering their hydraulic conductivity and transmissivity based on GLobal Hydrology MaPs 2.0 (GLHYMPS 2.0) (Gleeson et al. 2011); see Online Resource 1 for further information.

**Table 6:** Classification of aquifer productivity from negligible to high, of aquifer transmissivity from low to moderate and related risk of groundwater table decline for the two datasets used (based on: BGR 2019; Gleeson et al. 2011; Geological Survey Czech Republic 2007)

Aquifer class	Aquifer productivity “IHME“	Aquifer transmissivity “GLHYMPS 2.0” [m²/d]	Aquifer productivity “GLHYMPS”	Risk of groundwater table decline
Highly productive porous aquifers	High			Low
Highly productive fissured aquifers (including karstified rocks)	High			Low
Low and moderately productive porous aquifers & Low and moderately productive fissured aquifers (including karstified rocks)	Low/moderate	>100	Moderate	Moderate
		10.1–100	Moderate	Moderate
		1.1–10	Moderate	Moderate
		0.1–1	Low	High
		<0.1	Low	High
Locally aquiferous rocks, porous or fissured	Very low			Very high
Practically non-aquiferous rocks, porous or fissured	Very low/negligible			Very high

The degree of groundwater dependency of ecosystems relies on different conditions such as landscape morphology, the fraction of plants that access water from the saturated zone of the soil (Hatton and Evans 1998; Kuginis et al. 2016) and the depth to groundwater (Bell and Driscoll 2006; Froend and Loomes 2006). The authors concluded that the greater is the depth to groundwater, the lower is the requirement for groundwater and the more tolerant is the ecosystem to groundwater drawdown. Following, the degree of groundwater dependence of all GDEs is determined according to the present ecosystem group (Online Resource 1) and the average annual depth to the groundwater table (Table 7).

**Table 7:** Ecosystems’ degree of groundwater dependence (Based on: Hatton and Evans 1998; Froend and Loomes 2006; Eamus et al. 2006; Bell and Driscoll 2006)

Depth to groundwater ‘wetlands’	Depth to groundwater ‘terrestrial/trees’	Nature of groundwater dependence	Degree of groundwater dependence
≤1 m~	≤3 m°	Highly dependent; obligate use or obligate/ facultative mixed	Very high
1.1–2 m~	3.1–6 m°	Facultative dependence	High
2.1–3 m~	6.1–10 m°	Opportunistically use/ Individuals’ dependence	Moderate
>3 m~	>10’	No apparent dependence	Low

° Froend and Loomes 2006; ~ Bell and Driscoll 2006; Eamus et al. 2006

The aggregation of the degree of groundwater dependence and the risk of groundwater table drawdown to one value of the sensitivity of the site to changes in the groundwater regime is implemented through an aggregation matrix (Online Resource 1). The matrix weights the degree of groundwater dependence two thirds and the risk of groundwater table drawdown one third.

#### Aggregation of impact risk to GDEs

A second matrix is used for the aggregation of the criteria endangerment of the site and sensitivity of the site to the final valuation of the potential risk for the GDEs (Online Resource 1). The evaluation of risk follows the logic that high sensitivity and high threat of a site increase the impact risk through agricultural water use. The aggregation rules assign a higher weight of two thirds to the sensitivity of site since the protective status has only limited possibilities to reflect actual endangerment of an ecosystem.

The risk of impact on the GDEs is projected to the surrounding agricultural areas via spatial buffers. GDEs that are at low or moderate risk to suffer from groundwater exploitation generate 1 km buffers; high and very high risk generate 2 km buffers. The agricultural areas within the buffer zones received impact values that are identical to the risk value of the affected GDE. Where buffer zones of different GDEs do overlay, the higher risk value is assigned to the agricultural area.

## Results

**Fig. 3:**
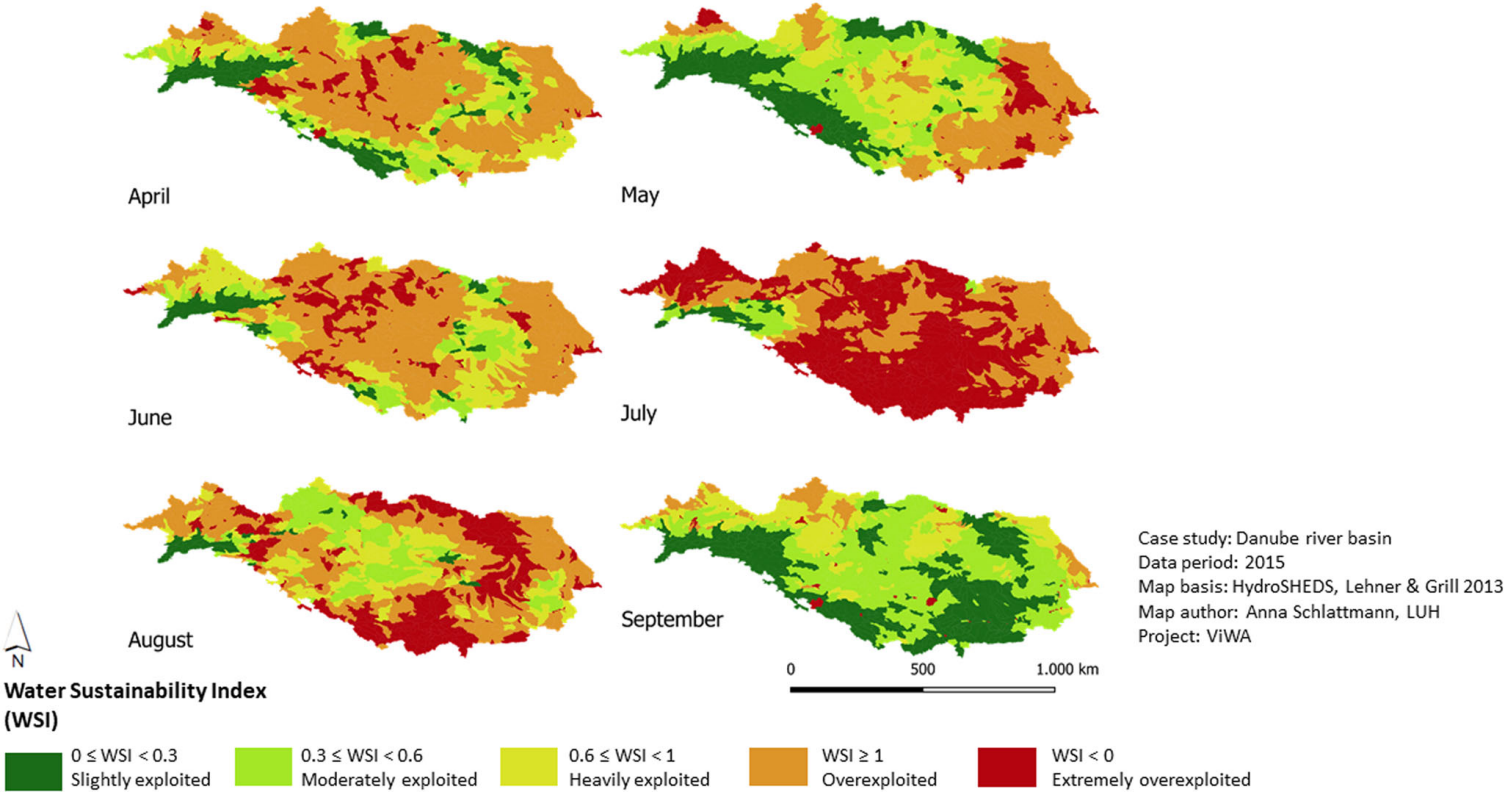
WSI results for the sub-basins of the Danube basin from April to September 2015. Green and yellow indicate sustainable index values, orange and red indicate unsustainable index values

**Fig. 4:**
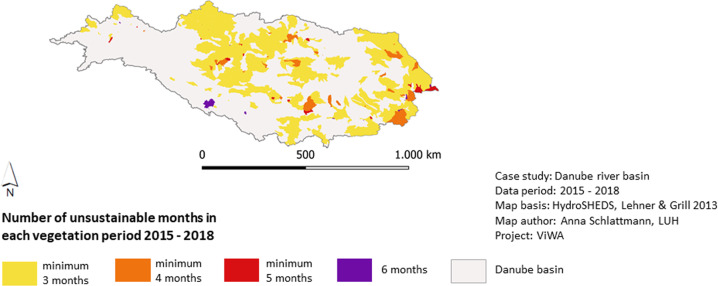
Summary of four-year period (2015–2018) for the WSI. Map shows all sub-basins that have an unsustainable water use for at least three months in each of the four vegetation periods

The Ecological Sustainability Assessment of Water Distribution, the ESAW-tool (Fig. 1) is developed according to the requirements outlined in sections “Introduction” and “Materials and Methods”. The translation of legitimate international sustainability standards for limits and priorities for sustainable water use into measurable indicators enables a water use sustainability assessment according to international norms within the Danube river basin. Thus, the selected standards cover the most important and the internationally most accepted norms for quantitative water use regarding ecological and social criteria. The multi-scale approach is realized through two assessment modules. The first module operates on sub-basin level. The second module provides results with 1 × 1 km grid cell resolution. The watershed sustainability assessment detects whether actual water demand can be satisfied staying within sustainable limits. The WSI results for the vegetation periods 2015–2018 show that the area with unsustainable water use varies between 6% (April 2017) and 93% (July 2015). “Extremely exploited” areas where even domestic and ecosystem water demands cannot be satisfied through the monthly available renewable water resources, occur on up to 58% (July 2015) of the basin area. In average (2015–2018) water use is most sustainably in September (33% unsustainable use) and most unsustainable in August and July (73% and 64% unsustainable use). The course during the vegetation periods shows a strong increase of unsustainable use between May and August (Fig. 3). Considering the spatiotemporal distribution of the availability-demand situation it can be stated that on 56% (2016) to 75% (2018) of the basin water use is unsustainable for at least three months within one vegetation period. Observing all studied vegetation periods 2015–2018, 38% of the basin area are managed unsustainably for minimum three months in each vegetation period (Fig. 4). Figures 3 and 4 show that areas under high pressure are predominantly located in the middle Danube and in the lower Danube, where agricultural activity is high.

**Fig. 5:**
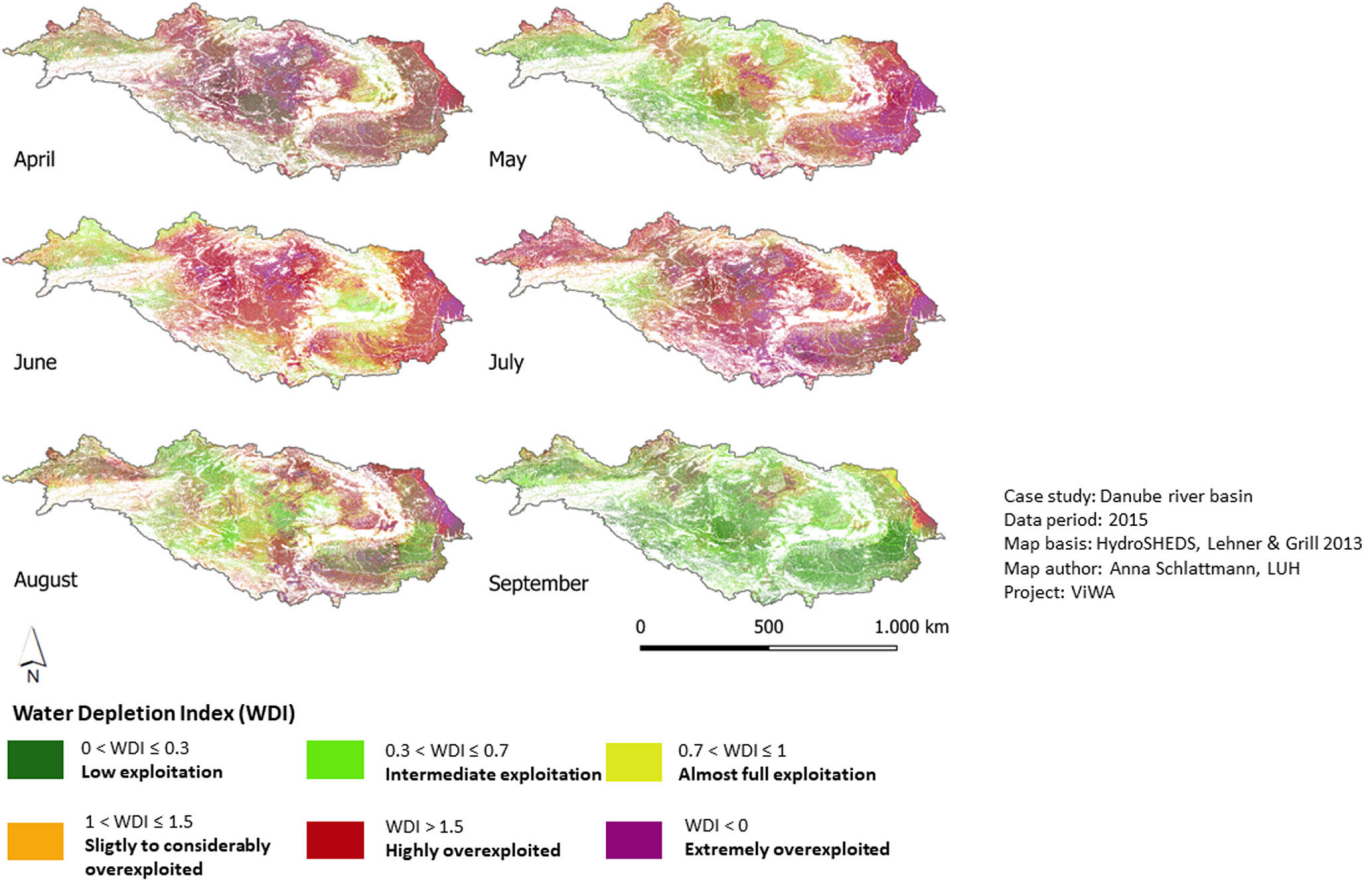
WDI results for the vegetation period 2015 in the Danube basin. Green and yellow indicate sustainable index values, orange, red and purple indicate unsustainable index values

The ecological risk assessment applies the three indicators WDI, e-flows and risk for groundwater-dependent ecosystems to detect areas where actual water use affects the performance of water sensitive ecosystems. The indicator results are presented separately and as overlaid Hot Spot analysis. According to the WDI results the highest exploitation appears in July 2015 where 84% of the agricultural area, predominantly located in the middle and lower Danube, is overexploited. Lowest exploitation is in September 2015 and September 2018 where 87% of the Danube basin stays within sustainable water exploitation limits (Fig. 5). However, the average over the studied period shows that the exploitation level is relatively constant between April and August with 57–63% of the area used unsustainably.

**Fig. 6:**
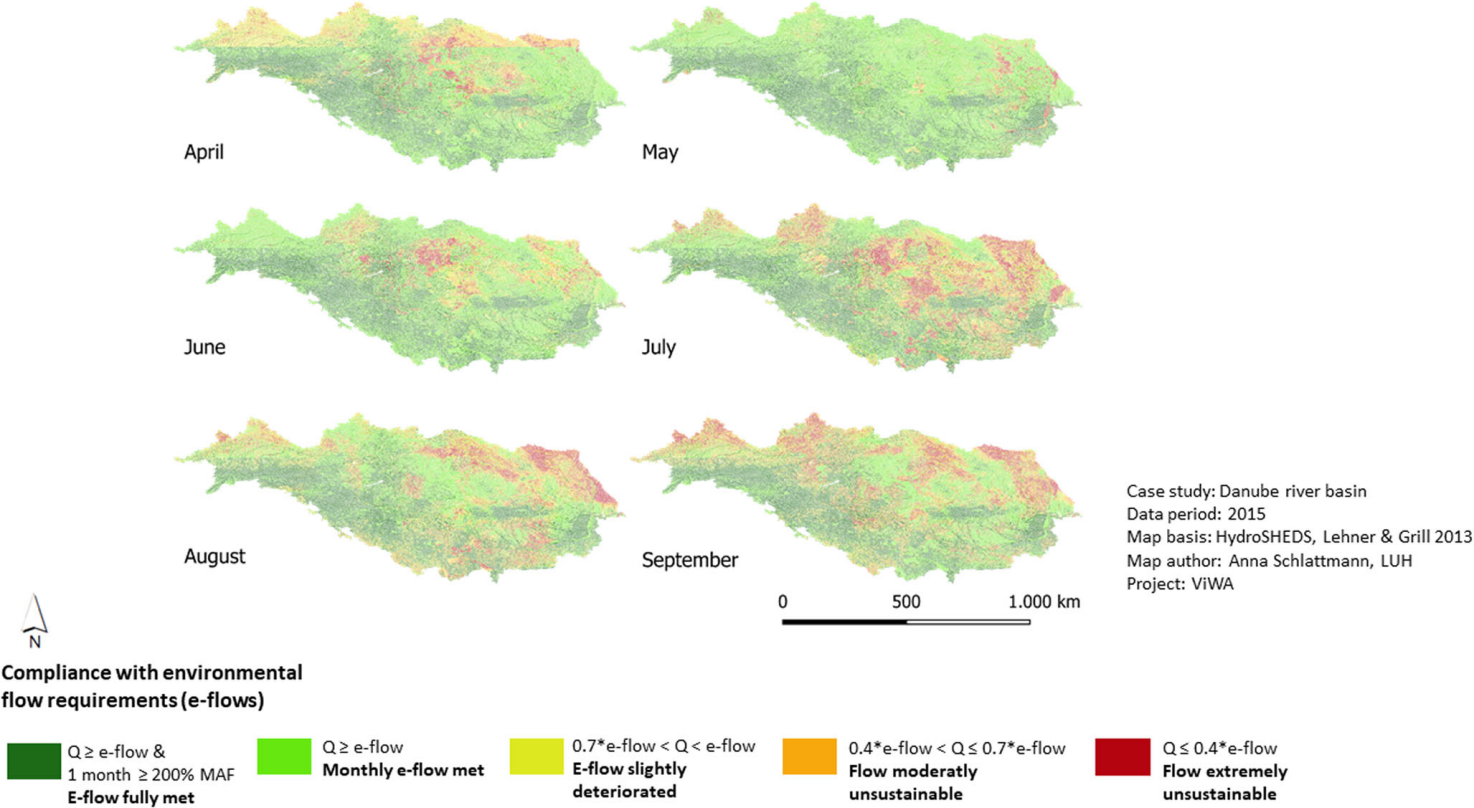
Results for compliance with e-flows from April to September 2015 the Danube basin. Green and yellow indicate compliance with e-flows, orange and red indicate no compliance with e-flows

The assessment of environmental flows shows where stream flows are sufficient to maintain aquatic ecosystems and their connected wetlands. The compliance with flow requirements is highest in May 2016 and June 2016 with 94% and 95% of the area having sustainable flows. The lowest compliance appears in September 2015 where 40% of the area shows unsustainable flows. These peaks are also reflected by the monthly averages: flow requirements are met best in May (90% of the basin) and June (86% of the basin). Areas that fall below required flows are predominantly located in the middle and lower Danube. At the beginning of the vegetation period also the northern part of the upper Danube show unsustainable flows. However, compared to the WDI results, the e-flow assessment shows a less critical situation. Additionally, it can be stated a time shift of the most unsustainable months towards the end of the vegetation periods (Fig. 6).

**Fig. 7:**
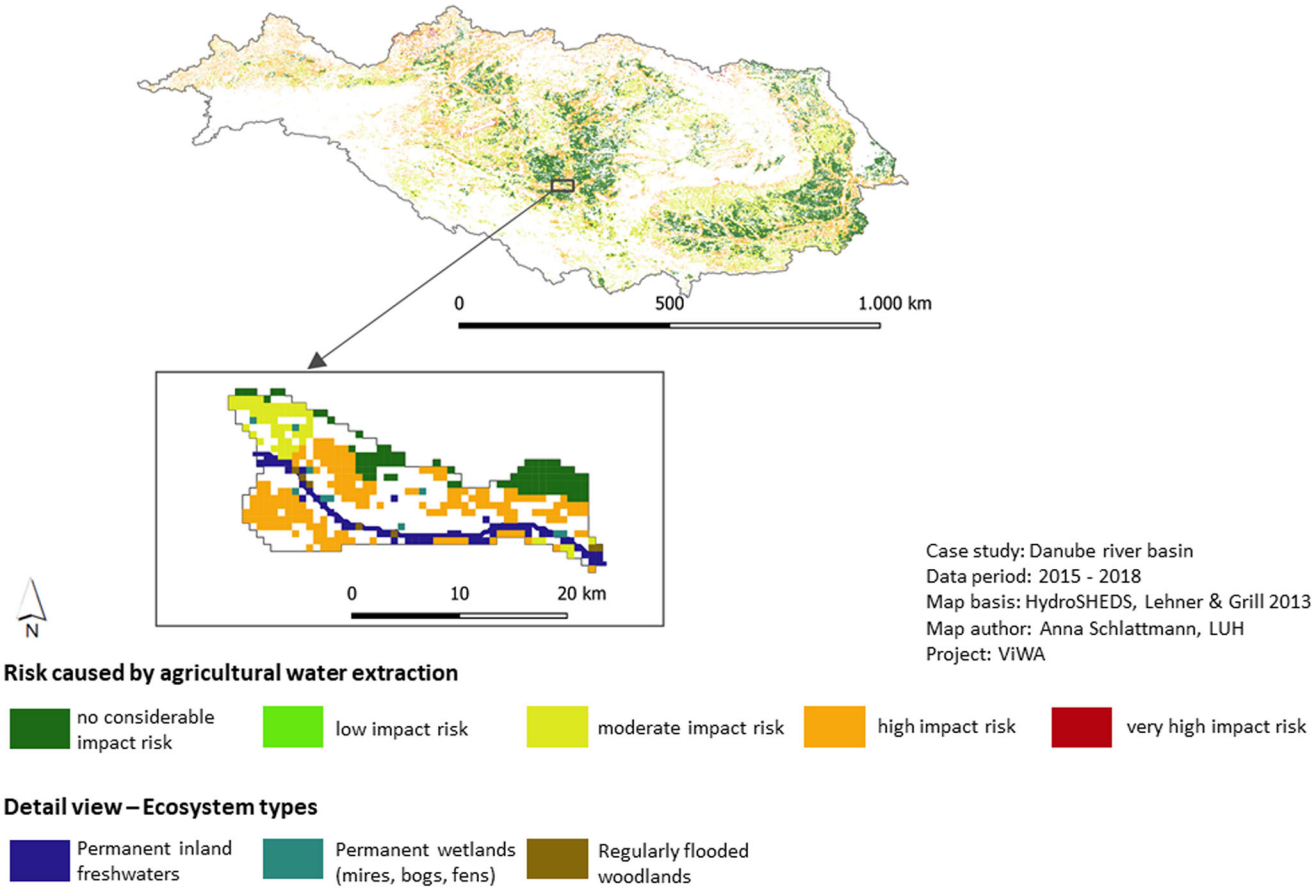
Risk of agricultural water use to impair GDEs in the Danube basin. Overview and detailed view with intersection with ecosystem map

The last indicator identifies agricultural areas where water overuse causes a risk for the maintenance and functionality of groundwater-dependent ecosystems. The present study indicates that 29% of the agricultural area, is at “high risk” or “very high risk” area to impair groundwater-dependent ecosystems. Regarding the spatial distribution of the critical agricultural areas in the Danube basin, it can be stated that they are predominantly located close to the Danube main river and its larger tributaries. However, one third of the agricultural areas is not at risk to impair groundwater-dependent ecosystems at all. The intersection of the agricultural risk areas with the ecosystem map reveals that, in the considered sub-basin, particularly inland waters, inland wetlands but also floodplain wetlands could be endangered from water extractions (Fig. 7).

**Fig. 8:**
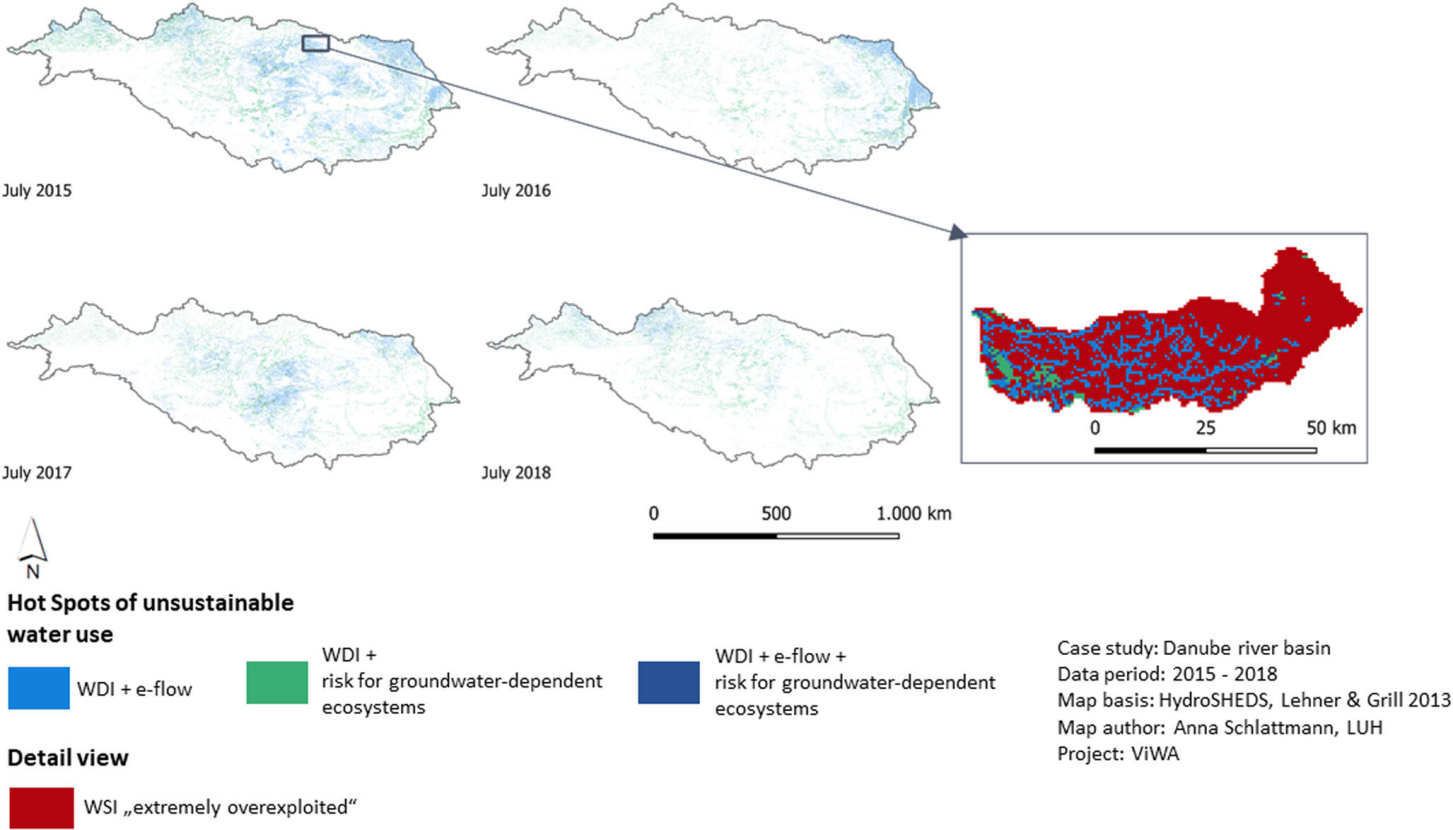
Hot Spots of unsustainable water use in July 2015–2018 in the Danube basin. Overview on the entire Danube basin and detailed view on a sub-basin overlaid with the WSI result for the respective month. The detail view shows that large areas of the sub-basin are Hot Spots of unsustainable water use

The combination of the indicator results allows to validate and to strengthen the assertion of the single results. The combination of a WDI and e-flow Hot Spot suggests that in these areas the unsustainable water exploitation for crop growth reasonably contributes to unsustainable stream flows in the same area (Fig. 8). The combination of WDI and risk for groundwater-dependent ecosystems indicates that the unsustainable water exploitation for crop growth realizes an actual risk for groundwater-dependent ecosystems in the respective areas. The combination of all three indicators characterizes complex interrelations of stressing factors that may affect each other. Summing up all three Hot Spot types, the largest coverage occurs in July 2017 (14,5% of the basin area). The monthly averages show that in April and June to August (7,8%; 7,5%; 8,5%; 8,7%) more Hot Spots occur than during the rest of the vegetation period. The Hot Spots are allocated over wide areas in the north-west, the centre and the delta region of the Danube basin (Fig. 8). The multi-scale analysis of Hot Spots and WSI results shows that in a sub-basin assessed as “extremely overexploited” 42% of the area are covered with Hot Spots (predominantly WDI + e-flow). This suggests that high agricultural water consumption can be the reason for sub-basin wide high pressure on renewable water resources particularly with effects on aquatic ecosystems.

## Discussion

The ESAW-tool is successfully implemented in a test case, the Danube basin. The ESAW-tool satisfactorily addresses the research goals: The developed indicators are applied in a spatial assessment. The results transparently show the limits for sustainable water use in physical maps. The indicators apply thresholds of ecosystem functioning and service provision as limits for sustainable water use. If applied, these limits for sustainable water use prevent ecosystems from degradation and losses of ecosystem services and biodiversity. The present results inform about sustainability of water use in the Danube basin on two spatial levels and with different detail applicable for various applications that will be discussed. Nevertheless, some shortcomings remain with respect to the complexity of the system, its proper representation in the model and data availability limitations.

### Linkage to Legitimate International Sustainability Standards

The indicators of the ESAW-tool are based on legitimate international standards for sustainable water use. The default use of these internationally accepted standards makes them universally applicable and transparently communicates the minimum requirements from international law. Particularly countries with only partially developed environmental law may benefit (Smakhtin et al. 2004b). However, agricultural water demand and industrial water demand may be undervalued in the evaluation scheme due to missing or very vague legitimate international standards and the lack of disaggregated data. In principle, the flexible methodology would allow the integration of additional water balance standards, i.e. by including technical water infrastructure, following the ideas of Wada et al. (2011a) for desalinated water use, or by adaptation of limits of water use. However, the integration of further standards such as “adequate water for food production” and “equitable and reasonable transboundary water allocation” require further legal specification before they can be transformed to spatially measurable indicators for water use, though the possibilities for standardized operationalization are viewed critically (McIntyre 2015). Nevertheless, some propositions have been made for operationalization (Beaumont 2000). The use of legitimate international sustainability standards for the construction of the assessment methodology makes it worldwide applicable.

### Consideration of Biodiversity and Related Ecosystem Services

The watershed sustainability assessment implemented through the WSI supply-demand ratio considers ecosystem water-demand through green-blue water demand. This is more comprehensive than previous large-scale estimates that base their sustainability estimates only on e-flows. The present results show that in parts of the Danube basin current water use restricts water availability of ecosystems below acceptable limits. Thus, ecosystem service provision and biodiversity of (semi-) terrestrial ecosystems and rivers will be reduced in the future. The present assessment identifies threatened areas in the middle and lower Danube basin. There, regionally and seasonally adapted water management is needed to maintain ecosystem service provision.

The ecological risk assessment informs through three indicators more detailed on the kind and effects of water overuse. The first indicator, the WDI shows high agricultural water depletion for large areas concentrated in the middle and lower Danube during summer months. As standalone indicator the WDI may considered weak as it is usual that crop or plant growth exploits soil water resources during the vegetation period and therefore can naturally exceed the amount of monthly renewed resources. Nevertheless, a high overuse can also be indicator for excessive irrigation with ground- or surface water which causes negative effects on their dependent ecosystems. The implemented combination of the WDI and the area covering e-flow assessment (Hot Spot) and the WSI that informs about the watershed sustainability, strengthens the assumption that agricultural water use is driver for the degradation of aquatic and (semi-) terrestrial ecosystems since a spatial relation between a high WDI, a reduced flow and a bad water balance can be identified (Fig. 8). This information should be used to select measures and their location for most beneficial water savings: In view of these results local authorities should consider restricting permissions for agricultural irrigation to maintain the services of rivers and their connected ecosystems. Otherwise, long-term flow reduction results in loss of fish species (and biodiversity in general) in the aquatic ecosystem as well as the degradation of connected floodplain ecosystems with important functions such as water purification and climatic regulation (Poff and Zimmerman 2010). The thresholds applied for the second indicator, the environmental flows, represent minimum values proposed by science (Pastor et al. 2014). Such thresholds are always arbitrary. To guarantee multiple ecological functions of aquatic ecosystems, according to the precautionary principle, the minimum flows should be even increased.

Finally, the risk assessment for groundwater-dependent ecosystems complements the ecological risk assessment by determining the risk for the interruption of ecosystem groundwater use caused by groundwater drawdown. Likewise, the combination with the WDI may show the relation to agricultural water use. The present overlay of the assessment results with an ecosystem map shows that permanent wetlands and flooded wetlands will be degraded if interruption of groundwater access continues (Fig. 7). The degradation of wetlands and mires significantly reduce their capacity for carbon sequestration (source) an important function to reduce greenhouse gases in the atmosphere (Mitch et al. 2015).

The pragmatic desktop approaches such as inferential GDE identification, the use of IUCN protected sites to estimate biodiversity conservation value and estimation of net water consumption via return-flow ratios could be improved by more elaborate methods such as Verones et al. (2017) for consideration of biodiversity or water footprint estimates (Mekonnen and Hoekstra 2011) to derive net water consumption. Likewise, simplistic e-flow calculation may be replaced by more detailed approaches or separate, comprehensive assessments to be found in the IHA-toolbox (The Nature Conservancy 2021; Richter et al. 1998). However, this would require more data input that reduces flexibility and impedes application in data scarce regions (Heink and Kowarik 2010).

### Practical Application in Water Management and Spatial Planning on Different Scales

The different components of the ESAW-tool enable a spatial evaluation of water use sustainability which is considered as powerful instrument to identify and to evaluate water use conflicts and their impacts on water dependent habitats as demanded by Russi et al. (2013). The multi-scale approach is realized through the two modules on sub-basin level (WSI) and grid cell level (ecological risk assessment). Due to the normative basis of the indicators including societal water demands, the provided results and their combination possibilities, the ESAW-tool can be used for strategical planning as well as for reactive approaches. Particularly the consideration of the normative dimension of sustainable water use qualifies the assessment results for application in strategical planning and implementation of instruments (von Haaren et al. 2019).

The WSI on sub-basin level addresses political decision-making and administrative activities on regional level. On the regional level the results can be used by water authorities or agents (1) in strategical planning; (2) for the application of existing instruments and (3) for obligatory monitoring tasks.

Strategical planning pursues comprehensive spatial planning to mediate conflicts between all users. The present results provide the required information to integrate water related objectives into sustainability strategies: The knowledge about areas that would not allow water intensive developments could be used to design funding programs with identified “target areas” where low consumptive technologies or ecosystem restoration projects would be most beneficial. The information can also be used to decide on far-reaching political decisions such as regional water limits or the promotion of large-scale irrigation to raise agricultural revenues, as it is presently discussed for some Danube basin states (Dogaru et al. 2019). In screening tasks for Strategical Environmental Assessment (SEA) or Environmental Impact Assessments (EIA), the ESAW-tool may help to identify the impact of planned changes of water use on sustainable water allocation, by using altered water use or land use patterns as input for the assessment tool. As EIA and SEA and official spatial planning basically takes decisions on the basis of applicable law, the international normative perspective of the ESAW-tool supports this legal orientation. The normative perspective and its transparent communication to the user is particularly important when assessment results are used to support decision-making for at least two reasons: (1) indicators and results are better accepted and taken up when the stakeholders approve the underlying norms (Heink and Kowarik 2010), since they are based on international legitimate sustainability standards and; (2) the assessment results can be used for prioritization of rights to water consumption, evaluation of goal attainment or compliance with legal norms (van Oudenhoven et al. 2018). Though, typically the regional administrations are not responsible for operational water resources management that includes extraction permissions for agriculture or industries. The second assessment module operates on the local level and provides results that can support local authorities’ decisions on water extractions. The more detailed approach helps to identify critically affected ecosystems that require higher water supply for defined periods during the vegetation period. The “actors” can deduce local measures in favour of prioritized ecosystems and their services such as the restriction to rain fed agriculture or to strategically initiate restoration projects. Moreover, the reactive steering particularly benefits from the multi-scale results. Often local authorities’ responsibility refers to administrative boundaries such as municipalities or districts that only cover a part of or parts of different hydrological (sub-)basins. The capacities of the administrations usually not allow intensive sub-basin wide water balance estimates. Thus, decisions are often taken without consideration of regional scale effects on water balance. The ESAW-tool offers an additional source of knowledge to administrations, which are restricted in their practices and capacities, to strengthen sustainable decision-making. Concluding, the present assessment methodology can provide the required information and improve decision-making processes across spatial and administrative levels and thus, helps to overcome problems of spatial fit.

Summarizing, we described broad application possibilities for administration and authorities but with direct effects on private persons such as private water users, investors or agencies. Furthermore, it is conceivable that development banks make similar considerations for awarding credits as public institutions can use assessment results for the design of funding programmes.

### Uncertainty and Applicability in Other Case Study Regions

The design of the methodology as a desktop tool and the applicability in standard GIS makes the tool flexible and suitable for different stakeholders and problems. The tool will be made available open access as QGIS toolbox (in prep.). For any application it is important to communicate sensitivity and inherent uncertainties of the ESAW-tool to potential users (Neuendorf et al. 2018). The results of the ESAW-tool are indexes that are based on the spatial quantitative relationships between variables provided by the input data. Therefore, the main sources of uncertainty of the present results are the uncertainties related to the input data (Neuendorf et al. 2021). The model is more sensitive to uncertainties related to the input hydrological variables than to the water use and water demand variables, due to the prevailing presence of the modelled hydrological variables in the index equations. The key input data for the model simulation is provided by the PROMET and OpenGeoSys models respectively for the surface and groundwater hydrological variables.

The physical processes within PROMET are fully validated in Mauser and Bach (2009) and Hank et al. (2015). The main uncertainties of PROMET results originate from the process description and the generalization of model set-up assumptions. Finally, PROMET uncertainties are related to the input data (SRTM, HWSD, HydroSHEDS, CORINE/ESA land cover) which propagate into the results.

The groundwater model OpenGeoSys is validated by comparing the steady and transient state results with the observations from 96 piezometers located within the basin. The accuracy of the model is supported by the high coefficient of determination (*R*² = 0.98) obtained when steady state results are compared with measured data. The main sources of uncertainty of the groundwater model are related to the hydrogeological parameters (i.e., the transmissivity and the storage coefficient (Pujades et al. 2020).

The uncertainty of the water use and water demand variables is much harder to determine and quantify due to the lack of validation and calibration possibilities. This uncertainty also increases by the need to spatially disaggregate the data to fit the requirements of the tool (described in sections “Domestic water demand, Industrial water consumption, Risk for groundwater-dependent ecosystems”). Despite the impact of the uncertainty of the input data on the results, the tool provides sufficient measures to identify Hot Spots of water exploitation for the screening part of the planning process when reliable data sources are used. To minimize the result uncertainties related to the input data, it is recommended that the scale of the input data corresponds to the scale of the final results that will be taken in consideration. However, the design of the methodology allows to substitute the regional input data with local data or to run the tool with input data deduced from functional (eco-) hydrological relationships, such as rooting depths (Schenk and Jackson 2002a), or data from global reviews (Schenk and Jackson 2002b). Thus, uncertainty and sensitivity of the results produced with the ESAW-tool are variable and largely depend on the chosen data input.

## Conclusions

The ESAW-tool is composed of a basic assessment that evaluates the sustainability of water allocation to different user types on sub-basin level and an additional component which differentiates the ecological risk of impairment for water dependent ecosystems. The methodology is globally applicable and suited for standard GIS applications and flexible data inputs. New components are the use of an evaluation framework based on legitimate international sustainability standards, transferred into operable indicators, which can be spatially applied; the incorporation of the risk for biodiversity; and a multi-scale approach leading to a new quality of results bridging different planning levels. These features are an important asset for the acceptability of the results and practical implementation.

The spatial river basin approach is suited to support decision-making in water use and distribution contexts for different kinds of users and problem contexts. The scale and data policies make the ESAW-tool useful for strategic water resources management and landscape planning since conflicts of water allocation to different ecosystem services can be assessed in a broader spatial context before passing over to local specification and implementation. As example, the application of the tool in the Danube basin demonstrates the temporal and spatial fluctuations of the water use sustainability in the area and identifies critical periods and users as well as vulnerable areas that require special consideration. Even if the Danube basin is globally not considered as directly threatened by water scarcity, the results prove clear need of regional and local redistribution of the water resources in some critical areas to improve the sustainability of the water use practices. Concluding, the ESAW-tool can support public authorities, business, funding agencies and further stakeholders that are involved in decisions on sustainable water management and the development of the agricultural sector. Thus, the ESAW-tool contributes to the achievement of the water-related Sustainable Development Goals.

## Supplementary information

ESM 1

ESM 2

ESM 3

ESM 4

## Data Availability

The ESAW-tool will be made freely accessible in a data repository as QGIS toolbox after the end of the research project.
