# Supplementary material for: Ecological Sustainability Assessment of Water Distribution for the Maintenance of Ecosystems, their Services and Biodiversity
Source: Environ Manage. 2022 Jun 14;70(2):329–49. doi: 10.1007/s00267-022-01662-3 (PMC9252940; doi:10.1007/s00267-022-01662-3)
Supplement: Supplementary file 1 — ESM 1 [file 267_2022_1662_MOESM1_ESM.pdf]

Environmental Management

Ecological sustainability assessment of water distribution for the maintenance of ecosystems, their services and biodiversity

Anna Schlattmann[1], Felix Neuendorf, Kremena Burkhard, Elisabeth Probst, Estanislao Pujades, Wolfram Mauser, Sabine Attinger, Christina von Haaren

[1]Leibniz University Hannover, Institute of Environmental Planning, Herrenhaeuserstr. 2, 30419 Hannover, Germany, schlattmann@umwelt.uni-hannover.de

Online Resource 1: Technical documentation

The present technical documentation explains the methodological details and operations carried out throughout the ESAW-tool. Figure 1 summarizes the technical details and inputs to the model.

1. Data & resources

The methodology was computed on a monthly basis in a Geoinformation System (GIS) (QGis v3.16). If nothing other is mentioned, in WGS 1984 coordinate system. The ESAW tool will be available as QGIS toolbox after the end of the ViWA research project (more information on the project: https://viwa.geographie-muenchen.de/).
The presented case study application uses for hydro-agroecological variables model data from the Processes of Mass and Energy Transfer model (PROMET) and groundwater variables from OpenGeoSys code, complemented by open access data on water use, biodiversity and ecosystems. All data sources used can be found in table 1.
The spatial resolution of raster input data is 0.0083333°. For the aggregation of results on sub-basin level HydroSHEDS level 8 (Lehner and Grill 2013) was used. Temporal resolution of most of the data is monthly. The input variables for the water scarcity assessment were computed in m³/month. Each month was considered with 30 days. The vegetation period was defined from April to September for the Danube basin. For the harmonization of different data source formats (vector and raster data) most data was transformed into vector grids that enabled work with multiple feature attributes and spatial overlays.

1.1. Mechanistic hydro-agroecological model PROMET

The key variables on the water balance in the Danube basin including Danube discharge and vegetation water use is obtained from the mechanistic hydro-agroecological model PROMET (Mauser and Bach 2009), simulating water and carbon fluxes in the Danube basin on hourly time steps with a spatial resolution of approx. 1 km (0.00833333°). Dynamic plant growth is computed within the PROMET vegetation component (Hank et al. 2015) using the approaches of Farquhar et al. (1980) and Chen et al. (1994). River discharge is simulated using the Muskingum-Cunge-Todini approach (Cunge 1969; Todini 2007). Bias-corrected ERA5 reanalysis data (Fick and Hijmans 2017; Hersbach et al. 2020) is used as meteorological driver for the simulations. Topography information is drawn from the Shuttle Topography Mission (SRTM) (Farr et al. 2007), soil data from the Harmonized World Soil Database (HWSD) (FAO et al. 2009) and land cover distribution from CORINE Land Cover 2012 (EEA 2012) (for EU states) and ESA CCI Land Cover 2015 (ESA Climate Change Initiative 2017) for non-EU states.

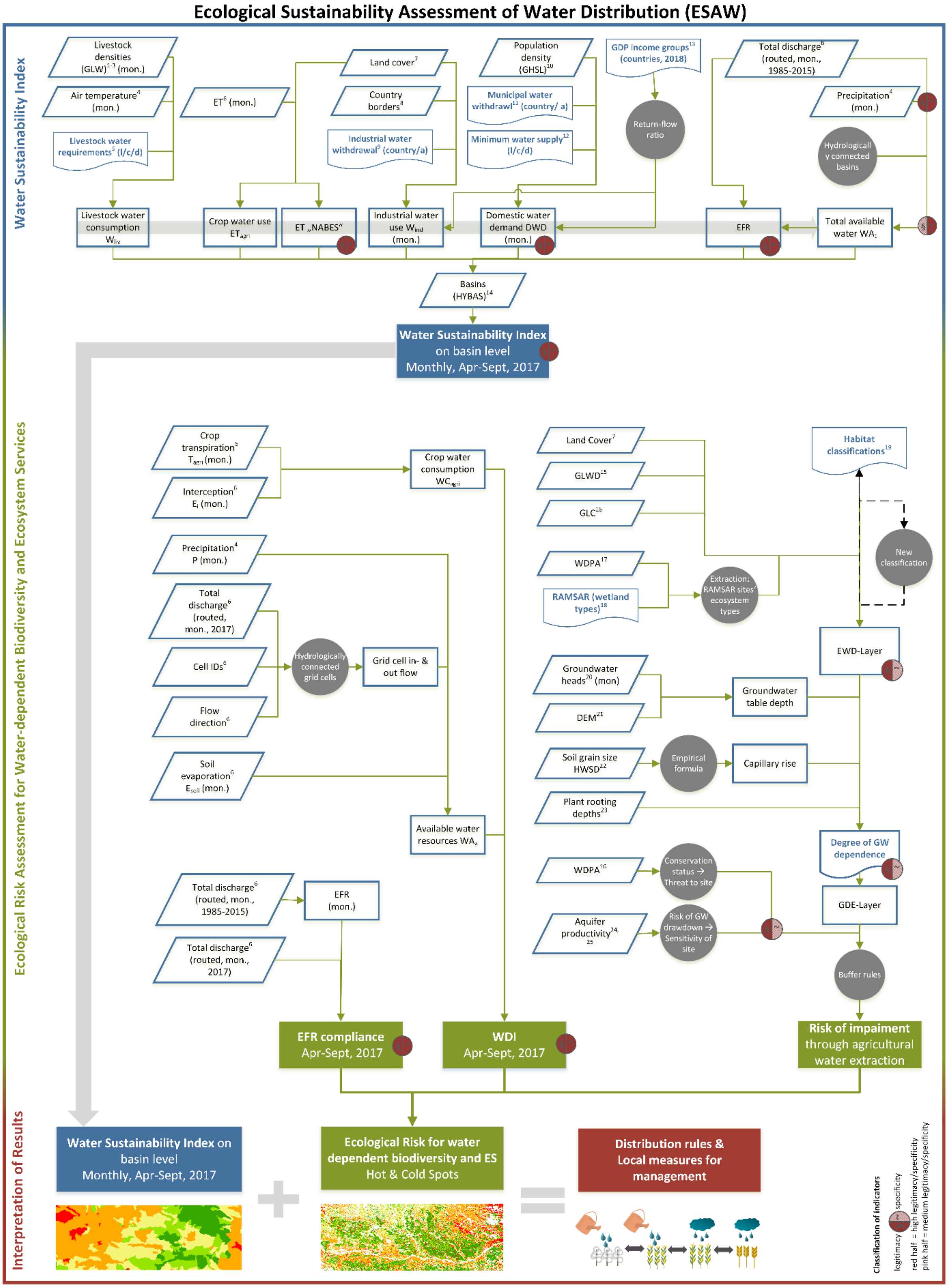

References: 1 Robinson et al. 2014; 2 Gilbert et al. 2018; 3 FAO 2020; 4 Copernicus Climate Change Service 2017; 5 Steinfeld et al. 2006; 6 PROMET model simulation, based on Mauser & Bach 2009 ; 7 Corine Land Cover, EEA 2012; Land Cover CCI; ESA 2017; 8 GADM 2018; 9 FAO/AQUASTAT 2019a; 10 Schiavina et al. 2019; 11 FAO/AQUASTAT 2019b; 12 Schlattmann et al. submitted; 13 World Bank 2018; 14 Lehner & Grill 2013; 15 WWF 2004; 16 European Commission, Joint Research Centre 2000; 17 IUCN, UNEP-WCMC 2019; 18 RSIS 2019; 19 EEA 2019; 20 OpenGeoSys model simulation, Pujades et al. 2019; 21 SRTM; Farr et al. 2007; 22 FAO/IIASA/ISRIC/ISSCAS/JRC, 2012; 23 Fan et al. 2017; 24 BGR 2019 (IHME1500); 25 Gleeson et al. 2011 (GLHYMPS).

Figure 1: Flow Chart of the Ecological Sustainability Assessment of Water Distribution (ESAW) with its two key components – Water Sustainability Index and Ecological Risk Assessment for water-dependent biodiversity and Ecosystem Services including key variables and information on legitimacy and specificity of the indicators. Parallelograms: geospatial data; waved rectangle: non-spatial data input or information from literature review; unfilled rectangle: interim results; green circle: data processing; filled rectangle: results; solid lines: one directional process; dashed line: iterative process; mon. = monthly resolution; a = annual resolution, l/c/d = liter per capita per day

1.2. Hydrogeological numerical model – OpenGeoSys code

Groundwater data is obtained from a hydrogeological numerical model developed with the code OpenGeoSys (Kolditz et al. 2012). The numerical model covers the whole Danube basin with a resolution of 500 m and allows computing the dynamics of shallow groundwater systems. It is a 2D model and the third dimension (i.e., aquifer thickness) is implemented through the aquifer's transmissivity, whose distribution is derived by analysing the spectral signal of the river baseflow measured at multiple gauging stations (Pujades et al. 2020). The values for storage coefficient were obtained from the GLobal Hydrology MaPs 2.0 (GLHYMPS 2.0) proposed by Gleeson et al. (2011). The numerical model was validated by comparing computed and observed groundwater head evolution at different locations. Groundwater elevation data is used to estimate groundwater dependence of ecosystems.

Table 1: Input variables and data sources for the application of the ESAW tool in the Danube basin

Variable	Unit (in Source)	Resolution	Data format	Source
Water Scarcity Assessment				
Country borders			Vector shape	GADM 2018
Municipal water withdrawal	Capita/a		Table/Statistics	FAO 2019b
Population density	Heads	1*1 km	Raster	GHSL; Schiavina et al. 2019
Country income groups based on GDP			Table	World Bank 2020
Industrial water withdrawal	Country/a		Table/Statistics	FAO 2019a
Danube basin delineation			Vector shape	HydroSHEDS lev. 3; Lehner and Grill 2013
Danube sub-basin delineation			Vector Shape	HydroSHEDS lev. 8; Lehner and Grill 2013
Land Cover		30 arc seconds/ 0.0083333°	Raster	Corine Land Cover (CLC), EEA 2012; Land Cover CCI; ESA Climate Change Initiative 2017
Evapotranspiration (all land cover)	mm/month	30 arc seconds/ 0.0083333°	Raster	PROMET
Livestock densities (cattle, goat, sheep, pig, chicken)	Heads	30 arc seconds/ 0.0083333°	Raster	GLW 2.01; Robinson et al. 2014
Livestock densities (buffalo)	Heads	5 minutes of arc/ 0.083333°	Raster	GLW 3; Gilbert et al. 2018
Livestock densities (horses)	Heads	Country	Table	FAO 2020
Air temperature	°Celsius	30 arc seconds/ 0.0083333°	Raster	ERA5, Copernicus Climate Change Service (C3S) 2017
Total discharge (routed); 2017	m^3/s	30 arc seconds/ 0.0083333°	Raster	PROMET
Total discharge	m^3/s	30 arc seconds/	Raster	PROMET

(routed); 1985-2015		0.0083333°		
Water Risk Assessment				
Precipitation	mm/month	30 arc seconds/ 0.0083333°	Raster	ERA5, Copernicus Climate Change Service (C3S) 2017
Soil evaporation	mm/month	30 arc seconds/ 0.0083333°	Raster	PROMET
Depression storage evaporation	mm/month	30 arc seconds/ 0.0083333°	Raster	PROMET
Total discharge	m^3/s	30 arc seconds/ 0.0083333°	Raster	PROMET
Flow directions		30 arc seconds/ 0.0083333°	Raster	PROMET
Transpiration	mm/month	30 arc seconds/ 0.0083333°	Raster	PROMET
Interception evaporation	mm/month	30 arc seconds/ 0.0083333°	Raster	PROMET
Global Lakes and Wetlands (GLWD)			Vector Shapes	WWF 2004
Global Land Cover 2000 (GLC)		1km at Equator (0.0089285714 dd)	Raster	EC 2004
World Database of Protected Areas (WDPA)		Polygons	Vector Shapes	UNEP-WCMC 2019
Ramsar Sites Information (wetland types)			Table	RSIS 2019
EUNIS habitat classification 2007			Table	EEA 2019
Crosswalk between EUNIS habitat classification 2007 and Habitat Directive Annex I habitat types 2008			Table	EEA 2008
Groundwater elevation	m; monthly	30 arc seconds/ 0.0083333°	Raster	Pujades et al. 2020
Digital Elevation Model (DEM)	m	1 degree	Raster	SRTM; Farr et al. 2007
Harmonised World Soil Database (HWSD)		30 arc seconds/ 0.0083333°	Raster	FAO et al. 2009
Key Biodiversity Areas		Polygons	Vector Shape	BirdLife International and Conservation International 2018
Hydrogeological Map of Europe (IHME 1500 v1.2)		Polygons	Vector Shape	BGR 2019
Global Hydrogeology MaPS 2.0 (GLHYMPS)		Polygons	Vector Shape	Gleeson et al. 2011

2. Water Scarcity modelling

2.1. Water demand

2.1.1. Domestic Water Demand (DWD)

Statistical data for municipal water withdrawals per capita was used to create a vector grid with per capita water consumption for the basin states. Country borders are based on (GADM 2018). Municipal per capita water withdrawal (FAO 2019b) was used for gross domestic water consumption and was compared with required minimum values for domestic water supply (figure 2). If, in a country, the statistical per capita water consumption was below required minimum supply, the medium-term target for domestic water demand was assumed as required domestic water demand instead of the statistical values. Domestic water demand was computed constant over the year. The most recent data for each country (range from 2008-2016) was used for the assessed years (2015 - 2018). The daily per capita water demand was projected on months and multiplied with population density from Global Human Settlement Layer (GHSL) (Schiavina et al. 2019) with 1*1 km resolution. In order to estimate the net water consumption, return-flow ratios as suggested by Wada et al. (2011a) were applied. Return-flow ratios depend on the Gross Domestic Product (GDP) of a country (World Bank 2020): 80 % return-flow in developed countries (high-income countries); 65 % return-flow in emerging countries (middle-income countries) and 40 % return-flow for developing countries (low-income countries) (table 2). The estimated, gridded domestic water demand was aggregated to sub-basin water demand (HydroSHEDS, lvl. 8) (Lehner and Grill 2013).

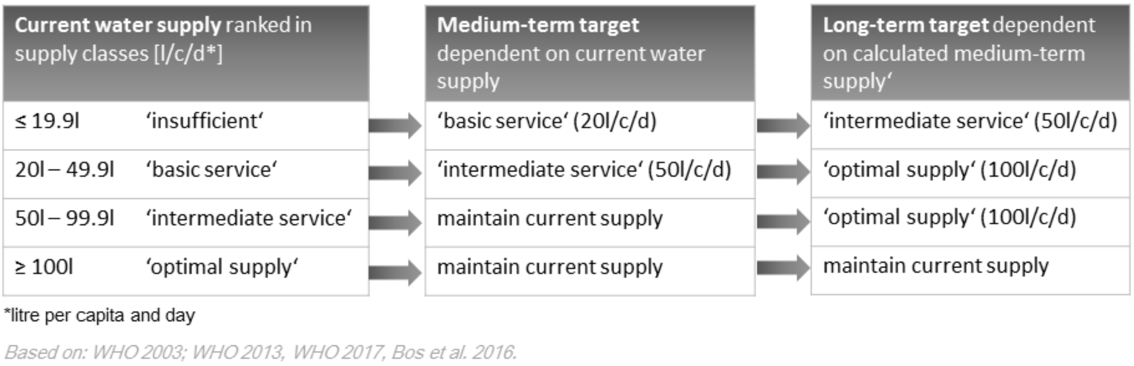

Figure 2: Minimum amount for domestic water supply for global medium-term and long-term targets depending on the current water supply situation. Recommendations based on WHO (from Schlattmann et al., submitted)

Table 2: Income groups based on GDP and related return-flow ratios for the Danube basin countries (based on: World Bank 2020; Wada et al. 2011).

Country	Income Group	Return-flow ratio
Austria	High income	80%
Switzerland	High income	80%
Czech Republic	High income	80%
Germany	High income	80%
Croatia	High income	80%
Hungary	High income	80%
Italy	High income	80%
Poland	High income	80%
Slovak Republic	High income	80%
Slovenia	High income	80%
Ukraine	Lower middle income	65%

Moldova	Lower middle income	65%
Albania	Upper middle income	65%
Bulgaria	Upper middle income	65%
Bosnia and Herzegovina	Upper middle income	65%
North Macedonia	Upper middle income	65%
Montenegro	Upper middle income	65%
Romania	Upper middle income	65%
Serbia	Upper middle income	65%

2.1.2. Industrial water consumption (W_{ind})

Statistical values for annual industrial water extraction were obtained from (FAO 2019a). For spatial disaggregation of the country data an even distribution of water extraction in each basin country was assumed. This means the share of industrial water withdrawn in the part of the country lying within the Danube basin is equivalent to the share of land area of that country lying within the Danube basin. The spatial intersect in GIS was carried out based on GADM (2018) country borders and HydroSHEDS level 3 (Lehner and Grill 2013) for the Danube basin delineation. Subsequently, the total amount of industrial water extracted within the share of a country lying in the Danube basin was divided by the amount of grid cells with "industrial built up" from 1*1 km resolution land cover mapping (PROMET setting) (EEA 2012; ESA Climate Change Initiative 2017). This water volume was allocated to all grid cells with industrial "land use". Grid cell values were divided by twelve in order to obtain monthly gross industrial water consumption. Net water consumption was computed similar to net domestic water demand (2.1.1.). The estimated, gridded industrial water consumption was aggregated to sub-basin level (HydroSHEDS, lvl. 8).

2.1.3. Agricultural water consumption

Agricultural water consumption is composed of crop water consumption (ET_{agri}) and livestock water demand (W_{liv}). Crop water consumption is equated to modelled crop evapotranspiration (PROMET). Monthly evapotranspiration [mm/month] of all grid cells with agricultural crops according to land use mapping (table 3) was summed up for each sub-basin, for each month from April to September of the years 2015 – 2018 and converted to m³/month.

Table 3: Agricultural crops and their grid-codes modelled by PROMET.

Code in land cover map	Agricultural area	Code in land cover map	Agricultural area
1	Extensive Grassland	15	Summer Wheat
2	Intensive Grassland	16	Winter Barley
3	Silage	17	Winter Wheat
4	Forage	31	Rice
5	Hop	33	Soybean
6	Legumes	34	Sunflower
7	Maize	35	Cotton
8	Oat	36	Vegetables
9	Oleaginous/Rapseed	37	Fruits and Berries
10	Potatoes	38	Wine
11	Rye	39	Olives
13	Sugar Beet	40	Winter Triticale
14	Summer Barley		

The calculation of livestock water demand is based on considerations of Wada et al. (2011b; 2011a). To obtain grid cell water demand per breed, livestock densities were multiplied with breed specific daily per capita water demands that are based on temperature and service water requirements (table 4). For livestock densities following datasets were used: Gridded Livestock of the World for cattle, goat, sheep, pig, chicken (GLW Version 2.01, 1 km spatial resolution, reference 2010) (Robinson et al. 2014) and for buffalo (GLW version 3) (Gilbert et al. 2018); and averaged horses density per country in 2018 (FAO 2020) in the Danube basin. Temperature dependent daily drinking water requirements were calculated based on the average monthly air temperature of the months April September (2015 – 2018) (Copernicus Climate Change Service (C3S) 2017; Fick and Hijmans 2017) for each grid cell within the Danube basin. The daily breed specific service water requirement was added to the drinking water demand for each grid cell. For the study region weighted service water requirements were assumed with 25 % industrial and 75 % grazing livestock systems based on the European agriculture characterization (Meyer 2014). Daily water requirements for each breed were multiplied by 30 to obtain monthly values. Finally, all water requirements for the different breeds were summed up and subsequently aggregated on sub-basin level.

Table 4: Livestock water requirements based on temperature and livestock systems averaged for the Danube basin (Based on: (Steinfeld et al. 2006; Luke 1987; SCARM 2003; FAO 2006).

Species	Physiological condition	Drinking water requirements based on air temperature [°C] in [l/animal/d]			Service water [l/animal/d]		
		15 ≤ 19.9	25 20 - 29.9	35 ≥ 30	Industrial	Grazing	Average prod. Syst. (75% grazing, 25% industrial)
**Cattle**	Average large breed dry cows and mid-lactation	73.45	94	114.55	8.75	2.75	4.25
**Goat**	Lactating 0.2 litres milk per day	7.6	9.6	11.9	2.5	2.5	2.5
**Sheep**	Lactating 0.4 litres milk per day	8.7	12.9	20.1	3.5	2.5	2.75
**Chicken*100 /poultry**	Average adult broilers and laying eggs	15.45	29.45	56.25	6.5	6.5	6.5
**Pigs (swine)**	Lactating daily weight gain of pigs 200g	17.2	28.3	46.7	60	17	27.75
**Horses***		87	129	201	2.5	5	4.375
**Buffalo****		95.485	122.2	148.915			0

*allowance for horses based on Luke 1987

** allowance for buffalo 30% more than cattle, based on SCARM 2003

2.1.4. Ecosystem water demand

Ecosystem water demand is composed of water demand of natural and semi natural ecosystems (ET_{nat}) and Environmental Flow Requirements (e-flows). Monthly evapotranspiration of natural and semi natural ecosystems was calculated similar to crop water consumption (see 2.1.3.). Land use codes of natural and semi natural ecosystems types can be found in table 5.

Table 5: Ecosystem types considered natural and semi natural

Code in land cover map	Natural and semi natural ecosystem types
20	Deciduous forest
21	Conifer forest
22	Rock
23	Wetland
24	Alpine
26	Glacier
25	Natural grass
27	Water

The calculation of E-FLOWs is based on the quantitative flow approach from Tessmann (1980), documented in Pastor et al. (2014) and Hatfield and Paul (2015) and others. The type of hydrological months was computed for the months April to September based on long-term mean monthly flow (MMF) and mean annual flow (MAF) (OSU 2005b, 2005a) in m^3/s of the period 1985-2015 (data modelled in PROMET) (table 6). Based on the type of the hydrological month each grid cell was assigned a recommended monthly flow for the months April to September, respectively. The recommended flow that should exit a sub-basin during the months April to September is estimated from the recommended flow (m^3/s) at the outlet-point of the respective basin. Outlet points of the sub-basins (HydroSHEDS lvl. 8) were identified through selection of the highest discharge value among the grid cells of a basin. For the E-FLOW demand of each month of the vegetation period the total discharge in m^3/s was multiplied by 60*60*24*30 to obtain m^3/month.

Table 6: Environmental flow requirements recommended by Tessmann (1980)

Type of hydrological month	Rule	Recommended Monthly Flow
Low-flow-month	$MMF_{long\text{-}term} \leq 40\%$ of $MAF_{long\text{-}term}$	100% of $MMF_{long\text{-}term}$
Intermediate-flow-month	$MMF_{long\text{-}term} > 40\%$ of $MAF_{long\text{-}term}$ & 40 % of $MMF_{long\text{-}term} \leq 40$ % of $MAF_{long\text{-}term}$	40% of $MAF_{long\text{-}term}$
High-flow-month	$MMF_{long\text{-}term} > 40\%$ of $MAF_{long\text{-}term}$ & 40 % of $MMF_{long\text{-}term} > 40$ % of $MAF_{long\text{-}term}$	40% of $MMF_{long\text{-}term}$

2.2. Water supply

The water supply within a sub basin was computed as sum of i) internal renewable water resources (WRt), where $WR_t(i)$ is precipitation, and ii) the inflow from upstream sub-basins that is left over after upstream water extractions have been taken. For the computation of the two components following method was applied:

i) Grid-cell precipitation data (ERA5) (Copernicus Climate Change Service (C3S) 2017) (Eq. 1) was summed for each sub-basin, based on sub-basin IDs (HydroSHEDS lvl. 8).

$$WRt(i) = P \qquad (1)$$

ii) A vector layer with data on total discharge, E-FLOWs, DWD, IWC and livestock water demand was prepared and dissolved for sub-basins. Following, E-FLOWs, DWD, IWC and livestock water demand were subtracted from total discharge to get remaining discharge (Eq. 2; 3). Then, flow relations were established between the sub-basins. The HydroSHEDS layer indicates the next downstream basin via IDs. The remaining discharge of all sub-basins with equal downstream neighbour was summed. The field calculator was used to assign the summed discharge values as “inflow” to the respective sub-basins based on basin IDs. Sub-basins without upstream neighbours got inflow value “0”. Inflow and precipitation of each sub-basin were summed to WAt (Eq. 4).

$$Qout\ (i) = \max\left(0, WAt(i) - WD(i)\right) \qquad (2)$$

$$WD(i) = Wliv(i) + ETagri + Wind(i) + DWD(i) + EFR(i) + ETnabes(i) \qquad (3)$$

$$WAt(i) = WRt(i) + \sum Qout\ (iup) \qquad (4)$$

Note: WA_t = total available water; WRt = internal renewable water; Q_{up} = outflow from upstream basins into basin; P = precipitation; Qout = out flow from the basin; WD = water demand

2.3. Water Sustainability Index (WSI)

For the computation of the Water Sustainability Index (WSI), the previously computed variables of water demand and water supply were compiled into one vector layer for sub-basins. The WSI was calculated in GIS according to equations (5) and (6) and was subsequently classified in scarcity classes (table 7).

$$WSIviwa = \frac{ETagri + Wliv + Wind}{WAt - Wprio} \qquad (5)$$

$$W_{prio} = DWD + EFR + ETnat \qquad (6)$$

Note: WSI_{viwa} = Water Scarcity Index; ETagri = agricultural water use for crop growth; Wliv = agricultural water use for livestock production; W_{ind} = industrial water consumption; WA_t = total available water; Wprio = priority water use; DWD = domestic water demand; E-FLOW = environmental flow requirements; ET_{nat} = evapotranspiration of natural and semi natural ecosystems; WRt = internal renewable water; Q_{up} = outflow from upstream basins into basin

Table 7: Classification of WSI from slight exploitation to extreme overexploitation in five classes depending on the degree of exploitation of sustainably usable water resources, adopted from (Smakhtin et al. 2004).

WSI (proportion)	Degrees of environmental-social water scarcity of river sub-basins	GIS class
WSI < 0	Extremely overexploited (monthly sustainably usable water is lower than sum of priority uses, fossil water or surplus of preceding months is used)	5
WSI ≥ 1	Overexploited (current water exploitation is higher than allowed sustainable levels)	4
0.6 ≤ WSI < 1	Heavily exploited (0 to 40% of sustainably usable water is still available in a sub-basin)	3
0.3 ≤ WSI < 0.6	Moderately exploited (40% to 70% of the sustainably usable water is still available in a sub-basin)	2
0 ≤ WSI < 0.3	Slightly exploited (70 % or more of the sustainably usable water is still available in a sub-basin)	1

3. Impact risk assessment for water dependent biodiversity and Ecosystem Services

3.1. Agricultural Water Depletion Index (WDI)

For the WDI agricultural areas were selected via land cover type (table 3). Available renewable water resources (WA_a) were estimated by summation of precipitation, soil evaporation (including depression storage evaporation) and inflow to the grid cell from upstream grid cells minus total discharge from the grid cell on a monthly basis (April – September (2015 – 2018)) (Eq. 7). Inflow from upstream grid cells was estimated from total discharge and flow directions based on a digital elevation model (DEM, SRTM; (Farr et al. 2007) (Eq. 8, 9). In order to establish cell relations, total discharge and flow direction data was transformed into vector features and then combined via spatial join. The resulting attribute table indicates cell IDs and cell IDs of the downstream neighbour. The attribute table was exported in Microsoft Excel table calculator. Cells without downstream neighbour (discharge into cell itself) were deleted. With the function of pivot tables all upstream neighbours of each cell were identified via cell IDs and their discharge was summed up. The summed discharge constitutes the inflow to the grid cells. After analysis was completed the excel file was transformed to .dbf format and joined to the initial vector grid. All variables were converted into same unit km³/month. Agricultural crop water use (WC_{agri}) for the selected areas was estimated by summation of monthly crop water consumption which is composed of transpiration and interception evaporation (Eq. 10). As before, all variables were converted into km³/month. Then, the ratio of WC_{agri} and WA_a was calculated (Eq. 11). The classification of index values was carried out according to table 8.

$$WAa = P + Qin - Qout - Esoil \qquad (7)$$

Where,

$$Qout = \ total\ discharge \qquad (8)$$

and,

$$Qin = Qout(up) \qquad (9)$$

$$WCagri = Tagri + Ei \qquad (10)$$

$$WDI = \frac{WCagri}{WAa} \qquad (11)$$

Note: WDI = Water depletion index; WC_{agri} = agricultural water consumption (for crop growth); WAa = available renewable water resources on agricultural areas; Tagri = crop transpiration; E_i = interception evaporation; P = precipitation; Q_{in} = inflow to grid cell; Q_{out} = outflows from grid cell; E_{soil} = soil evaporation including depression storage evaporation

Table 8: Thresholds for the classification of the WDI values of agricultural areas.

WDI	Degree of exploitation of water resources generated in grid cell	GIS class
WDI < 0 (neg.)	Local available water is < 0, meaning that cell outflow is higher than monthly water input	6
WDI > 1.5	Crop water exploitation is significantly higher than available water resources	5
1 < WDI ≤ 1.5	Crop water exploitation is slightly to considerably higher than available water resources	4
0.7 < WDI ≤ 1	0.7 < WDI ≤ 1 Almost full crop water exploitation of available water resources	3
0.3 < WDI ≤ 0.7	Intermediate crop water exploitation, recharge and storage of soil moisture possible	2
0 ≤ WDI ≤ 0.3	Low crop water exploitation, recharge and storage of soil moisture possible	1

3.2. Environmental Flow Requirements

Recommended minimum flows on grid cell level were taken from E-FLOW calculations in section 2.1.4. The calculated E-FLOWs were compared to the modelled total discharge of the respective months of April – September (2015 – 2018). The ratio of E-FLOW to modelled total discharge was classified according to table 9. The classification of category “1”, “E-FLOWs fully met”, was carried out in two steps: 1) The discharge of all months of the year was compared to MAF. Grid cells where discharge is ≥ 200% of MAF in at least one month of the year were selected. Out of this selection all cells were selected that were classified in category “2”, ”monthly E-FLOW met”, in all months of the vegetation period and were assigned the new category “1”, ”E-FLOWs fully met”.

Table 9: Degrees of E-FLOW compliance.

Q surplus or shortage to recommended E-FLOWs	Degrees of compliance with E-FLOWs	GIS class
Q ≥ E-FLOW and Q of one month of the year is ≥ 200% MAF	E-FLOWs fully met - healthy river conditions that can be regarded as near natural	1
Q ≥ E-FLOW	Monthly E-FLOW met – good status of river flow, only functions depending on seasonal extreme events cannot be met	2
0.7 E-FLOW < Q < E-FLOW	E-FLOW slightly deteriorated	3
0.4 E-FLOW < Q ≤ 0.7 E-FLOW	E-FLOW moderately deteriorated	4
Q ≤ 0.4 E-FLOW	River flow extremely unsustainable	5

3.3. Impact risk for groundwater dependent ecosystems (GDEs)

3.3.1. Identification of GDEs

The identification of GDEs was carried out in two steps: 1) identification and mapping of potentially water-dependent ecosystem types (Ecosystem Water Dependency, EWD) through review of existing literature about water dependency and relevant geodata; and 2) analysis of the pre-selected areas regarding their actual dependence on groundwater based on site specific characteristics.

Pre-selection for EWD: A global typology of Ecosystem Water Dependency was elaborated. Therefore, different global land cover datasets were overlaid: Global Lakes and Wetland Database (GLWD) (WWF 2004), Global Land Cover 2000 (GLC) (EC 2004), RAMSAR protected areas (World Database of Protected Areas (WDPA)) (UNEP-WCMC 2019) and Corine Land Cover (CLC 2012) (EEA 2012) / Land Cover CCI 2015 (ESA Climate Change Initiative 2017) map. In order to obtain detailed information on ecosystem types from the RAMSAR protected areas the information on the wetland type from the RAMSAR Sites information Service (RSIS 2019; Ramsar Conference of the Parties 2009) was joined to the geospatial mapped RAMSAR sites reported in the WDPA via site IDs. The RAMSAR information sheets report one to multiple different wetland types for each RAMSAR site, listed in descending order of their area contribution. The first wetland type indicated was chosen as wetland type of the RAMSAR site

From the overlaid land cover sets, a mapping was created that applies a new classification system that differentiates types of water-dependent ecosystems on the global scale. The classes were developed and assigned to each grid cell by overlay rules in an iterative process (see also ESM 2). The information of the individual data layers differs in part considerably. For the classification, different importance was assigned to the datasets: i.e. weight to the different data sets based on their purpose,

accuracy and reference date. The Corine/ CCI Land Cover was used as basis and serves for the identification of crop land or built up areas irrespective of diverging information in the other datasets. For further differentiation of natural or semi-natural habitats with focus on their water dependence following rules were applied:

- If site information from RAMSAR is available it was most important for the classification.
- The second most important layer is the GLWD layer.
- The third most important layer was the Corine/ CCI Land Cover map.
- The GLC 2000 layer was used for clarification in terms of largely deviating information.
- Classification was guided by the precautionary principle. That means if the information of the different datasets is significantly diverging, the classification was oriented at the wettest land cover type

The derived land cover mapping and classification was exported to Microsoft Excel table calculator and then linked to the European University Information Systems (EUNIS) (EEA 2019; Davies et al. 2004) habitat classification levels two and three and EU Habitat Directive annex I habitats (EEA 2008). The land cover mapping was enriched with further information on habitats potential groundwater dependency from the EUNIS database itself and further literature (Reich et al. 2012; BfN 2006; EC 2013; EEA 2008) (ESM 3). The excel table with the additional information was transformed in .dbf format and joined to the spatial mapping in GIS. All grid cells with habitat types that are unlikely to depend on further water resources than precipitation (table 10) were deleted from the layer. The category marine and coastal ecosystems were included only for the wetlands of the Danube delta that are related to the inland water supply and flows, others were excluded from the "EWD-Layer".

Table 10: Global classification of ecosystem types according to their potential water dependence "EWD" with ecosystem groups.

EWD Code	Ecosystem type	Water dependence "EUNIS + FFH"; ()= likely water source	Ecosystem group
**101**	**Inland surface waters**		
41	Lakes	w (aq)	wetland
42	Permanent freshwater lakes (>8ha), incl. Floodplain lakes	w (aq)	wetland
43	Seasonal/Intermittent freshwater lakes (>8ha), incl. Floodplain lakes	w (aq)	wetland
44	Permanent saline, brackish or alkaline lakes	w (aq)	wetland
45	Seasonal/Intermittent saline, brackish or alkaline lakes and flats	w (aq)	wetland
46	Rivers	w (aq)	wetland
47	Permanent Rivers, streams or creeks, incl. inland deltas	w (aq)	wetland
48	Freshwater springs, oases	w (aq)	wetland
49	Seasonal/Intermittent Rivers, streams or creek	w (aq)	wetland
**50**	**Wetlands (mires, bogs and fens)**	w	wetland

51	Permanent freshwater marshes, pools, marshes and swamps on inorganic soils; with emergent vegetation water-logged for at least most of the growing season (herb dominated)	w, (GW)	wetland
52	Permanent or seasonal wetlands, incl. Pan, Brackish/Saline/alkaline marshes or pools	w, (GW)	wetland
53	Seasonal/Intermittent freshwater marshes/pools on inorganic soils, incl. Sloughs, potholes, seasonally flooded meadows, sedge marshes (Herb dominated)	w, (GW)	wetland
54	Alpine wetlands, incl. Alpine meadows, temporary waters from snow melt	w, (GW)	wetland
55	Wetland, non-forested bogs, fens and mires, incl. Shrub cover	w, (GW)	wetland
	**Grasslands**		
56	Natural Grassland (fresh)	t-b	terrestrial/trees
57	Wet or fresh grasslands and hydrophillous tall-forb stand, degenerated or drained wetlands	w, (GW/U/S)	wetland
58	Alpine grasslands (fresh)	t-b	terrestrial/trees
	**Heathland, shrub and tundra**		
59	Shrub Cover, closed-open, evergreen or deciduous	t	terrestrial/trees
60	Wetland, regularly flooded with shrub or herbaceous vegetation on organic or inorganic soils	w	wetland
61	Tundra wetlands, incl. Tundra pools, temporary waters from snow melt	w	wetland
	**Woodland, forest and other woodland**		
62	Deciduous woodland, closed or open	t-w (predominantly dry)	terrestrial/trees
63	Regularly/seasonally flooded woodlands and tree-dominated wetlands, incl. Freshwater swamp forests, wooded swamps on inorganic soils, Forested peatlands, peatswamp forests	w	wetland
64	Wetland, regularly flooded, saline water with tree cover	w	wetland
65	Broadleaved evergreen woodland	t-w (predominantly dry)	terrestrial/trees
66	Needle-leaved/coniferous, evergreen or deciduous woodland	t-w (predominantly dry)	terrestrial/trees
68	Mixed broadleaved and coniferous woodland	t-w (predominantly dry)	terrestrial/trees
70	Tree Cover burnt	t (exclude)	terrestrial/trees
	**Complexes**		

71	Mosaic: Tree cover / Other natural vegetation	t-w	terrestrial/trees
	**Other habitats beyond our scope**		
72	Alpine mixed	t (exclude)	terrestrial/trees
73	Built up	t (exclude)	artificial
74	Crop Land	t (exclude)	crop land
75	Dry/fresh Extensive Grassland	t (exclude)	terrestrial/trees
76	Intensive Grassland	t (exclude)	terrestrial/trees
77	Karst and other subterranean hydrological systems, inland	w (exclude)	wetland
78	Artificial water bodies, reservoirs, treatment areas, channels, inc. combined with natural water bodies or wetlands or terrestrial habitats	w (anthrop)	wetland
79	Unvegetated or sparsely vegetated habitats	t (exclude)	unspecified
80	Marine or Coastal mixed landscapes (deltas)	w (coast)	wetland
81	Human-made wetlands incl. wet agricultural landscapes (drained agricultural peatlands, sewage fields, …)	w (anthrop)	wetland

Note: column "water dependence" indicates likely additional water sources to precipitation: w = water dependence not nearer defined, w(aq) = aquatic habitat with and without groundwater dependence, w(GW) = groundwater dependence, w(U) = influenced by flooding, w(S) = waterlogged areas, w(anthrop) = anthropogenic wetlands, undefined water source, w(coast) = coastal or marine habitats mostly influenced by salt water, t-b = dry to fresh types, t-w = dry to moist/wet types, t(exclude) = dry, no apparent water dependence. All types shaded in red were excluded from further analysis.

Identification of GDEs: The effective groundwater dependence of ecosystems was estimated through the combination of the EWD-Layer with additional site-specific information on groundwater levels and vegetation characteristics, in the following called "GDE-layer".

Monthly groundwater elevation data (2015-2018) from the UfZ (Pujades et al. 2020) was combined with the Digital Elevation Model (DEM) (Farr et al. 2007) to compute the monthly groundwater depth from surface. Groundwater depth and modelled rooting depth data (Fan et al. 2017) were transformed from raster into vector format and joined to the EWD-layer which forms the new GDE-layer. Rooting depths for permanently aquatic ecosystems where set to zero.

The height of the capillary rise was estimated by an empirical formula that is based on soil pore diameters (Rowell 1994) (Eq. 12). The Harmonised World Soil Database (HWSD) (FAO et al. 2009) attribute S_USDA_TEX_CLASS provided information on main soil texture. Pore diameter and capillary rise were deduced from simplified relationships between particle size and pore diameters (Blume et al. 2010; Boley 2012; Rowell 1994) (table 11). Averaged particles sizes for the different soil texture groups were used to calculate pore diameter (Eq. 13) and capillary rise (Eq. 12). Where no information on soil texture is available in the HWSD a default value of 350 cm of capillary rise, oriented at PROMET model assumptions of capillary rise, was applied. The USDA texture class codes served for the join with the vector grid.

$$C_h = 3000/d \qquad (12)$$

$$d = K/5 \qquad (13)$$

Note: C_h = height of capillary rise [cm]; d = diameter of soil pores [µm]; K = diameter of particles [µm]

A grid cell was identified as GDE if groundwater connection is given in at least one month of each of the vegetation periods 2015-2018. Groundwater connection is given when plant roots reach into groundwater or capillary fringe (Eq. 14) or, groundwater is permanently shallow. Shallow groundwater is given when mean monthly depth to the groundwater table of the period 2015-2018 was 100 cm or less during at least one month of the vegetation period (April – September) (Eq. 15).

$$GW_{dep} = GW_d - R_d - C_h \leq 0 \tag{14}$$

$$GW_{dep} = GW_d(MM_{veg2015-2018}) \leq 100cm\, for\, min. 1\, month \tag{15}$$

Note: GW_{dep} = groundwater dependent; GW_d = depth to groundwater table; R_d = average maximum plant rooting depth; GW_d (MM_{veg}) = mean monthly depth to groundwater table during for the month of the vegetation period

All grid cells that do not full fil one of the conditions of equations (14) or (15) were deleted from the GDE-layer.

Table 11: Textural classes of the HWSD grouped into texture groups and linked to grain size-groups, averaged grain sizes and deduced average pore diameters and capillary rise. (Based on: (Boley 2012; Blume et al. 2010)

S_USDA_TEX_CLASS	Textural class	Texture group	Grain size group [µm]	Texture sub-group	Grain size sub-group [µm]	Average grain diameter [µm]	Average pore diameter [µm]	Capillary rise [m]
1	clay (heavy)	Clayey soils	0 - 2	very fine texture	0,2 - 0,63	0.415	0.083	361.45
3	clay			very fine texture	0,2 - 0,63	0.415	0.083	361.45
2	silty clay			fine texture	0 - 2	1	0.2	150.00
8	sandy clay			fine texture	0 - 2	1	0.2	150.00
4	silty clay loam*	Loamy soils	2-63	moderately fine texture	6 - 20	13	2.6	11.54
10	sandy clay loam			moderately fine texture	6 - 20	13	2.6	11.54
5	clay loam*			moderately fine texture	6 - 20	13	2.6	11.54
6	silt	Loamy soils		medium texture	2 - 63	32.5	6.5	4.62
7	silty loam			medium texture	2 - 63	32.5	6.5	4.62
9	loam			medium texture	2 - 63	32.5	6.5	4.62
11	sandy loam*	Loamy soils	63 - 2000	moderately coarse texture	200 - 630	415	83	0.36
12	loamy sand	Sandy soils		Coarse texture	63 - 2000	1031.5	206.3	0.15
13	sand			Coarse texture	63 - 2000	1031.5	206.3	0.15

3.3.2. Evaluation of site-specific risk

3.3.2.1. Endangerment of site

The GDE-layer was overlaid with protected areas reported in the WDPA and the database of Key Biodiversity areas (KBA) (BirdLife International and Conservation International 2018) and transformed to a 1*1 km vector grid. GDEs that lay within protected areas according to IUCN, or RAMSAR with the status "inscribed", "established", "designated" or "adopted" or are confirmed Key Biodiversity Area (KBA), Area of Zero Extinction (AZE) or Important Bird Area (IBA) were assigned the respective category of the endangerment of the site (table 12).

Table 12: Endangerment of the site based on the conservation status.

Protective status	Endangerment of site
• IUCN cat. 1a, 1b, 4<br>• RAMSAR	Very high (4)
• IUCN cat. 2	High (3)
• IUCN cat. 5, 6, (3)<br>• IBA, AZE, KBA	Moderate (2)
• None	Low (1)

3.3.2.2. Sensitivity of site

For the evaluation of the sensitivity of a site two indicators were applied: 1) the risk of groundwater table decline and 2) the degree of groundwater dependence. The aquifer productivity served as proxy to estimate the risk of groundwater table decline caused by groundwater pumping. The digitized International Hydrogeological Map of Europe (IHME 1500 v1.2) (BGR 2019) was used to determine the productivity of aquifers that lie under the GDEs as presented in table 13. For all aquifers that are classified as "Low and moderately productive porous aquifers" and "Low and moderate productive fissured aquifers" the GLobal HYdrogeology MaPS 2.0 (GLHYMPS 2.0) (Gleeson et al. 2011) was additionally consulted to divide them into aquifers of low productivity and moderate productivity. To that issue the respective aquifers were selected from IHME map and were intersected with the GLHYMPS 2.0 map. The permeability values of GLHYMPS 2.0 were converted into hydraulic conductivity and aquifer transmissivity values (Eq. 16). For the classification of the aquifers according to table 12 the transmissivity values were averaged for the extent of the aquifers in IHME.

$$T = 10^{\frac{k}{100}} * 1e + 7 * 100 \tag{16}$$

Note: T = transmissivity [m²/d] of a 100 m thick aquifer; k = permeability [m²]

Table 13: Classification of aquifer productivity from negligible to high, of aquifer transmissivity from low to moderate and related risk of groundwater table decline for the two datasets used (based on: BGR 2019; Gleeson et al. 2011; Geological Survey Czech Republic 2007)

Aquifer class	Aquifer productivity „IHME“	Aquifer transmissivity "GLHYMPS 2.0" [m²/d]	Aquifer productivity "GLHYMPS"	Risk of groundwater table decline
Highly productive porous aquifers	High			Low (1)
Highly productive fissured aquifers (including karstified rocks)	High			Low (1)
Low and moderately productive porous aquifers & Low and moderately productive fissured aquifers (including karstified rocks)	Low/Moderate	>100	Moderate	Moderate (2)
		10.1 – 100	Moderate	Moderate (2)
		1.1 – 10	Moderate	Moderate (2)
		0.1 – 1	Low	High (3)
		< 0.1	Low	High (3)
Locally aquiferous rocks, porous or fissured	Very low			Very high (4)
Practically non-aquiferous rocks, porous or fissured	Very low/Neglegible			Very high (4)

The degree of groundwater dependence of the GDEs was estimated based on their ecosystem-type being either terrestrial or semi-terrestrial/ wetland (table 10 & 14).

Table 14: Ecosystems' degree of groundwater dependence (Based on: Hatton and Evans 1998; Froend and Loomes 2006; Eamus et al. 2006; Bell and Driscoll 2006)

Depth to groundwater 'wetlands'*	Depth to groundwater 'terrestrial/trees'**	Degree of groundwater dependence	Classes of dependency
≤ 1 m~	≤ 3 m°	Highly dependent; obligate use or obligate/ facultative mixed	Very high (4)
1.1-2 m~	3.1-6 m°	Facultative dependence	High (3)
2.1-3 m~	6.1-10 m°	Opportunistically use/ Individuals dependence	Moderate (2)
> 3 m~	>10"	No apparent dependence	Low (1)

°Froend and Loomes 2006; ~ Driscoll & Bell 2006; "Eamus et al. 2006

*wetland ES such as flood plain, mound spring, bog, fen, swamp (Table 10); ** terrestrial ES such as forest, woodland, shrub land (Table 10)

For the estimation of the sensitivity of the GDEs to alterations in the groundwater regime, the risk of groundwater table decline and the degree of groundwater dependence were combined through aggregation rules (table 15).

Table 15: Matrix for the aggregation of the indicators 'degree of groundwater dependence' and 'risk of groundwater table decline' to the valuation of the criterion 'sensitivity of site' in four classes from low to very high sensitivity

		Probability of groundwater drawdown			
		Very high 4	High 3	Moderate 2	Low 1
ES degree of groundwater dependence	Very high 4	4 (very high sensitivity)			
	High 3		3 (high sensitivity)		
	Moderate 2			2 (moderate sensitivity)	
	Low 1				1 (low sensitivity)

3.3.3. Evaluation of impact risk of agricultural water use to water dependent ecosystems

The risk of GDEs to experience negative impact through groundwater pumping was estimated by the combination of the endangerment of the site (section 3.3.2.1.) and the sensitivity of the site (section 3.3.2.2.) via aggregation rules similar to section 3.3.2.2. (table 16).

Table 16: Matrix for the aggregation of the criteria 'sensitivity of site' and 'endangerment of site' to the valuation of the impact of agricultural water use on groundwater-dependent ecosystems with a weighted aggregation of 2/3 and 1/3.

		Endangerment of site			
		Very high 4	High 3	Moderate 2	Low 1
Sensitivity of site	Very high 4	4 (very high impact)			
	High 3		3 (high impact)		
	Moderate 2			2 (moderate impact)	
	Low 1				1 (low impact)

The resulting risk values were projected on the adjacent agricultural areas with the GIS buffer tool. GDEs with risk categories 1 and 2 were buffered with 1 km, GDEs with risk categories 3 and four were buffered with 2 km. The agricultural grid cells within the buffer zones were selected and received an impact value. The impact value is the same as the risk category of the GDE that is buffered. Where buffer zones of different GDEs and/or risk values overlay, the greatest risk value was chosen as impact value.

References

1. Bell S, Driscoll C (2006) Vegetation of the Tomago and Tomaree Sandbeds, Port Stephens, New South Wales. https://www.researchgate.net/publication/274329074_Vegetation_of_the_Tomago_and_Tomaree_Sandbeds_Port_Stephens_New_South_Wales. Accessed 05 Nov 2020
2. BfN (2006) Wasserrahmenrichtlinie, wasserabhängige Lebensraumtypen nach Anhang I der FFH-Richtlinie. https://www.bfn.de/themen/natura-2000/management/kooperation-mit-nutzern/grundwassernutzung.html. Accessed 11 Nov 2020
3. BGR (2019) Extended vector data of the International Hydrological Map of Europe 1:1,500,000 (Version IHME 1500 v1.2). https://www.bgr.bund.de/ihme1500. Accessed 10 Nov 2020
4. BirdLife International, Conservation International (2018) Key Biodiversity Area (KBA) digital boundaries: September 2018 version. : http://www.ibatforbusiness.org. Accessed 31 Jul 2019
5. Blume H-P, Brümmer GW, Horn R, Kandeler E, Kögel-Knabner I, Kretzschmar R, Stahr K, Wilke B-M (2010) Scheffer/Schachtschabel: Lehrbuch der Bodenkunde, 16th edn. Springer Berlin Heidelberg, Berlin, Heidelberg
6. Boley C (ed) (2012) Handbuch Geotechnik, Grundlagen - Anwendungen - Praxiserfahrungen ; mit 185 Tabellen, 1. Aufl., Praxis. Vieweg+Teubner Verlag, Wiesbaden
7. Chen, D.-X., Coughenour, M. B., Knapp AK, Owensby CE (1994) Mathematical simulation of C4 grass photosynthesis in ambient and elevated CO2. Ecological Modelling 73: 63–80. https://doi.org/10.1016/0304-3800(94)90098-1
8. Copernicus Climate Change Service (2017) ERA5: Fifth generation of ECMWF athmospheric reanalyses of the global climate. https://cds.climate.copernicus.eu/cdsapp#!/home.
9. Cunge JA (1969) On The Subject Of A Flood Propagation Computation Method (Muskingum Method). Journal of Hydraulic Research 7: 205–230. https://doi.org/10.1080/00221686909500264
10. Davies CE, Moss D, Hill MO (2004) EUNIS Habitat Classification Revised 2004. Report to European Environment Agency, European Topic Centre on Nature Protectio and Biodiversity. European Environment Agency, Copenhagen
11. Eamus D, Froend R, Loomes R, Hose G, Murray B (2006) A functional methodology for determining the groundwater regime needed to maintain the health of groundwater-dependent vegetation. Aust J Bot 54: 97–114. https://doi.org/10.1071/BT05031
12. EC (2004) The Land Cover of the World in the Year 2000. https://forobs.jrc.ec.europa.eu/products/glc2000/glc2000.php. Accessed 11 Nov 2020
13. EC (2013) Interpretation Manual of the European Habitats, EUR 28. https://ec.europa.eu/environment/nature/legislation/habitatsdirective/. Accessed 09 Nov 2020
14. EEA (2008) Crosswalk between EUNIS habitat classification 2007 and Habitat Directive Annex I habitat types 2008. https://www.eea.europa.eu/data-and-maps/data/eunis-habitat-classification/documentation/link-between-eunis-2007-and.xls. Accessed 27 Nov 2020
15. EEA (2012) CORINE Land Cover (CLC) 2012. https://land.copernicus.eu/pan-european/corine-land-cover/clc-2012?tab=mapview. Accessed 11 Nov 2020
16. EEA (2019) EUNIS habitat classification 2007 (Revised descriptions 2012) amended 2019. https://www.eea.europa.eu/data-and-maps/data/eunis-habitat-classification/habitats/eunis-habitats-complete-with-descriptions.xls. Accessed 05 Nov 2020
17. ESA Climate Change Initiative (2017) Land Cover Project 2017 - Land Cover CCI 2015. https://maps.elie.ucl.ac.be/CCI/viewer/. Accessed 11 Nov 2020

18. Fan Y, Miguez-Macho G, Jobbágy EG, Jackson RB, Otero-Casal C (2017) Hydrologic regulation of plant rooting depth. Proc Natl Acad Sci U S A 114: 10572–10577. https://doi.org/10.1073/pnas.1712381114
19. FAO (2006) Livestock's long shadow, Environmental issues and options. Food and Agriculture Organization of the United Nations, Rome
20. FAO, IIASA, ISRIC, ISS-CAS, JRC (2009) Harmonized World Soil Database (version 1.1). http://www.fao.org/fileadmin/templates/nr/documents/HWSD/HWSD_Documentation.pdf. Accessed 13 Nov 2020
21. FAO (2019a) AQUASTAT Core Database. Industrial water withdrawal. http://www.fao.org/nr/water/aquastat/data/query/index.html?lang=en. Accessed 10 Oct 2019
22. FAO (2019b) AQUASTAT Core Database. Municipal water withdrawal. http://www.fao.org/nr/water/aquastat/data/query/index.html?lang=en. Accessed 10 Oct 2019
23. FAO (2020) Agricultural production - Livestock Horses. http://www.fao.org/faostat/en/#data/QA. Accessed 03 May 2020
24. Farquhar GD, Caemmerer S von, Berry JA (1980) A biochemical model of photosynthetic CO2 assimilation in leaves of C3 species. Planta 149: 78–90. https://doi.org/10.1007/bf00386231
25. Farr TG, Rosen PA, Caro e, Crippen R, Duren R, Hensley S, Kobrick M, Paller M, Rodriguez E, Roth L, Seal D, Shaffer S, Shimada J, Umland J, Werner M, Oskin M, Burbank D, Alsdorf D (2007) The Shuttle Radar Topography Mission. Reviews of Geophysics 45: 1–33. https://doi.org/10.1029/2005RG000183
26. Fick SE, Hijmans RJ (2017) WorldClim2: new 1-km spatial resolution climate surfaces for global land areas. International Journal of Climatology 37: 4302–4315. https://doi.org/10.1002/joc.5086
27. Froend R, Loomes R (2006) Determination of Ecological Water Requirements for Wetland and Terrestrial Vegetation - Southern Blackwood and Eastern Scott Coastal Plain. Centre for Ecosystem Management, Joondalup
28. GADM (2018) Administrative Country Borders. https://gadm.org/data.html. Accessed 25 Oct 2018
29. Geological Survey Czech Republic (2007) Hydrogeological Mapping. : http://www.geology.cz/projekt681900/vyukove-materialy/01_HG_final.pdf. Accessed 13 May 2020
30. Gilbert M, Nicolas G, Cinardi G, van Boeckl TP, Vanwambeke S, Wint W, Robinson TP (2018) Global buffaloes distribution in 2010 (5 minutes of arc). https://dataverse.harvard.edu/dataset.xhtml?persistentId=doi:10.7910/DVN/5U8MWI. Accessed 13 Jul 2020
31. Gleeson T, Smith L, Jansen N, Hartmann J, Dürr HH, Manning AH, Beek R von, Jellinek AM (2011) Mapping permeability over the surface of the earth. Geophysical Letters 38: L02401. https://doi.org/10.1029/2010GL045565.
32. Hank T, Bach H, Mauser W (2015) Using a Remote Sensing-Supported Hydro-Agroecological Model for Field-Scale Simulation of Heterogeneous Crop Growth and Yield: Application for Wheat in Central Europe. Remote Sensing 7: 3934–3965. https://doi.org/10.3390/rs70403934
33. Hatfield T, Paul AJ (2015) A comparison of desktop hydrologic methods for determining environmental flows. Canadian Water Resources Journal / Revue canadienne des ressources hydriques 40: 303–318. https://doi.org/10.1080/07011784.2015.1050459

34. Hatton T, Evans R (1998) Dependence of Ecosystems on Groundwater and its Significance to Australia, Occasional Paper No 12/98. Land and Water Resources Research and Development Corporation, Canberra
35. Hersbach H, Bell B, Berrisford P, Hirahara S, Horányi A, Muñoz-Sabater J, Nicolas J, Peubey C, Radu R, Schepers D, Simmons A, Soci C, Abdalla S, Abellan X, Balsamo G, Bechtold P, Biavati G, Bidlot J, Bonavita M, Chiara G de, Dahlgren P, Dee D, Diamantakis M, Dragani R, Flemming J, Forbes R, Fuentes M, Geer A, Haimberger L, Healy S, Hogan RJ, Hólm E, Janisková M, Keeley S, Laloyaux P, Lopez P, Lupu C, Radnoti G, Rosnay P de, Rozum I, Vamborg F, Villaume S, Thépaut J-N (2020) The ERA5 global reanalysis. Quarterly Journal of the Royal Meteorological Society 146: 1999–2049. https://doi.org/10.1002/qj.3803
36. Kolditz O, Bauer S, Bilke L, Böttcher N, Delfs JO, Fischer T, Görke UJ, Kalbacher T, Kosakowski G, McDermott CI, Park CH, Radu F, Rink K, Shao H, Shao HB, Sun F, Sun YY, Singh AK, Taron J, Walther M, Wang W, Watanabe N, Wu Y, Xie M, Xu W, Zehner B (2012) OpenGeoSys: an open-source initiative for numerical simulation of thermo-hydro-mechanical/chemical (THM/C) processes in porous media. Environmental Earth Sciences 67: 589–599. https://doi.org/10.1007/s12665-012-1546-x
37. Lehner, B, Verdin, K, Jarvis, A (2008) New Global Hydrography Derived From Spaceborne Elevation Data. Eos, Transactions American Geophysical Union, 89(10): 93-94. https://doi.org/10.1029/2008EO100001
38. Lehner B, Grill G (2013) Global river hydrography and network routing: baseline data and new approaches to study the world's large river systems. Hydrological Processes 27: 2171–2186. https://doi.org/10.1002/hyp.9740
39. Luke GJ (1987) Consumption of water by livestock. Department of Agriculture and Food, Western Austrailia, Perth
40. Mauser W, Bach H (2009) PROMET – Large scale distributed hydrological modelling to study the impact of climate change on the water flows of mountain watersheds. Journal of Hydrology 376: 362–377. https://doi.org/10.1016/j.jhydrol.2009.07.046
41. Meyer R (2014) Diversity of european farming Systems and Pathways to Sustainable Intensification. Technikfolgenabschätzung - Theorie und Praxis 23: 11–21. https://doi.org/10.1007/s10584-014-1169-1
42. OSU (2005a) Analysis Techniques: Annual Analysis. https://streamflow.engr.oregonstate.edu/analysis/annual/index.htm. Accessed 13 Jul 2020
43. OSU (2005b) Analysis Techniques: Monthly Analysis. https://streamflow.engr.oregonstate.edu/analysis/monthly/. Accessed 13 Jul 2020
44. Pastor AV, Ludwig F, Biemans H, Hoff H, Kabat P (2014) Accounting for environmental flow requirements in global water assessments. Hydrology and Earth System Sciences 18: 5041–5059. https://doi.org/10.5194/hess-18-5041-2014
45. Pujades E, Houben T, Di Dato M, Kumar R, Attinger S (2020) A European groundwater model with variable aquifer thickness derived from spectral analyses of baseflow. EGU General Assembly 2020, Online 4-8 May 2020, online, id.11140
46. Ramsar Conference of the Parties (2009) Strategic Framework and guidelines for the future development of the List of Wetlands of International Importance of the Convention on Wetlands (Ramsar, Iran, 1971). Third edition, as adopted by Resolution VII.11 (COP7, 1999) and amended by Resolutions VII.13 (1999), VIII.11 and VIII.33 (COP8, 2002), IX.1 Annexes A and B (COP9, 2005), and X.20 (COP10, 2008).

47. Reich M, Rode M, von Haaren C, Weiß C (2012) Regionales Management von Klimafolgen in der Metropolregion Hannover-Braunschweig-Göttingen, Teilprojekt 4 Klimawandel: lokales und regionales Naturschutzmanagement. Anhang zum Schlussbericht. Institut für Umweltplanung, Hannover
48. Robinson TP, Wint G, Conchedda G, van Boeckl TP, Ercoli, V., Palamara, E., Cinardi G, D'Aietti L, Hay SI, Gilbert M (2014) Mapping the Global Distribution of Livestock. PLoS ONE 9: e96084. https://doi.org/10.1371/journal.pone.0096084
49. Rowell DL (1994) Soil science, Methods and applications. Longman Scientific & Technical; Wiley, Harlow Essex, New York
50. RSIS (2019) Export Sites information. https://rsis.ramsar.org/ris-search?&pagetab=3. Accessed 05 Aug 2019
51. SCARM (2003) Farmed Buffalo, Model Code of practice for the Welfare of Animals. CSIRO Publishing, Collingwood Victoria
52. Schiavina M, Freire S, MacManus K (2019) GHS population grid multitemporal (1975, 1990, 2000, 2015) R2019A. http://data.europa.eu/89h/0c6b9751-a71f-4062-830b-43c9f432370f. Accessed 13 Nov 2020
53. Smakhtin V, Revenga C, Döll P (2004) A Pilot Global Assessment of Environmental Water Requirements and Scarcity. Water International 29: 307–317. https://doi.org/10.1080/02508060408691785
54. Steinfeld H, Wassenaar T, Jutzi S (2006) Livestock production systems in developing countries: status, drivers, trends. Rev. sci. tech. Off. int. Epiz. 25: 505–516. https://doi.org/10.20506/rst.25.2.1677
55. Tessmann S (1980) Environmental assessment, technical appendix e in environmental use sector reconnaissance elements of the western dakotas region of south dakota study. South Dakota State University, Brookings
56. Todini E (2007) A mass conservative and water storage consistent variable parameter Muskingum-Cunge approach. Hydrol Earth Syst Sci 11: 1645–1659. https://doi.org/10.5194/hess-11-1645-2007
57. UNEP-WCMC (2019) World Databse on Protected Areas (WDPA). : https://www.protectedplanet.net/c/world-database-on-protected-areas. Accessed 31 Jul 2019
58. Wada Y, van Beek LPH, Viviroli D, Dürr HH, Weingartner R, Bierkens MFP (2011a) Global monthly water stress: 2. Water demand and severity of water stress. Water Resour Res 47: 352. https://doi.org/10.1029/2010WR009792
59. Wada Y, van Beek LPH, Bierkens MFP (2011b) Modelling global water stress of the recent past: on the relative importance of trends in water demand and climate variability. Hydrol Earth Syst Sci 15: 3785–3808. https://doi.org/10.5194/hess-15-3785-2011
60. World Bank (2020) GNI income groups. https://data.worldbank.org/indicator/NY.GNP.PCAP.CD. Accessed 10 Feb 2020
61. WWF (2004) Global Lakes and Wetlands Database: Lakes and Wetlands Grid (Level 3). https://www.worldwildlife.org/publications/global-lakes-and-wetlands-database-lakes-and-wetlands-grid-level-3. Accessed 13 Nov 2020
