# Supplementary material for: Ecological Sustainability Assessment of Water Distribution for the Maintenance of Ecosystems, their Services and Biodiversity
Source: Environ Manage. 2022 Jun 14;70(2):329–49. doi: 10.1007/s00267-022-01662-3 (PMC9252940; doi:10.1007/s00267-022-01662-3)
Supplement: Supplementary file 2 — ESM 2 [file 267_2022_1662_MOESM2_ESM.pdf]

Environmental Management

Ecological sustainability assessment of water distribution for the maintenance of ecosystems, their services and biodiversity

Anna Schlattmann[1], Felix Neuendorf, Kremena Burkhard, Elisabeth Probst, Estanislao Pujades, Wolfram Mauser, Sabine Attinger, Christina von Haaren

[1]Leibniz University Hannover, Institute of Environmental Planning, Herrenhaeuserstr. 2, 30419 Hannover, Germany, schlattmann@umwelt.uni-hannover.de

Online Resource 2: Overlay rules for EWD typology

PROMET	GLC	GLWD	Ramsar	PROMET value	GLC value	GLWD value	RAMSAR value	EWD value
Water			Human-made wetlands, 2: Ponds	0	0	0	2	78
Water			Humand-made wetlands, 5: Salt exploitation sites	0	0	0	5	78
Water			Human-made wetlands, 6: Water storage areas	0	0	0	6	78
Water			Humand-made wetlands, 7: Excavations	0	0	0	7	78
Water			Humand-made wetlands, 8: Wastewater treatment areas	0	0	0	8	78
Water			Human-made wetlands, 9: Canals and drainage channels or ditches	0	0	0	9	78
			Inland wetlands, Zk(b): Karst and other subterrenean hydrological systems	0	0	0	Zk(b)	77

Water			Human-made wetlands, Zk(c): Karst and other subterrenean hydrological systems	0	0	0	Zk(c)	78
NA	Tree Cover, broadleaved, deciduous, closed			0	2	0	0	62
NA	Tree Cover, broadleaved, deciduous, closed	Lake		0	2	1	0	41
NA	Tree Cover, needle-leaved, evergreen			0	4	0	0	66
NA	Tree Cover, needle-leaved, evergreen		Marine or coastal wetlands, E: Sand, shingle or pebble shores, F: Estuarine waters, Inland wetlands, M: Permanent rivers/ streams/ creeks, O: Permanent freshwater lakes, P: Seasonal/ intermittent freshwater lakes, Tp: Permanent freshwater marshes/ pools,	0	4	0	E	80

NA	Tree Cover, needle-leaved, evergreen		Marine or coastal wetlands, J: Coastal brackish / saline lagoons, H: Intertidal marshes, F: Estuarine waters, E: Sand, shingle or pebble shores, G: Intertidal mud, sand or salt flats, Inland wetlands, Tp: Permanent freshwater marshes/ pools	0	4	0	J	80
NA	Tree Cover, needle-leaved, evergreen	Lake		0	4	1	0	41
NA	Tree Cover, needle-leaved, evergreen	Coastal Wetland		0	4	6	0	80
NA	Tree Cover, needle-leaved, evergreen	Coastal Wetland	Marine or coastal wetlands, E: Sand, shingle or pebble shores, F: Estuarine waters, Inland wetlands, M: Permanent rivers/ streams/ creeks, O: Permanent freshwater lakes, P: Seasonal/ intermittent freshwater lakes, Tp: Permanent freshwater marshes/ pools,	0	4	6	E	80
NA	Tree Cover, mixed leaf type			0	6	0	0	68
NA	Tree Cover, mixed leaf type	Coastal Wetland		0	6	5	0	80

NA	Shrub Cover, closed-open, evergreen			0	11	0	0	59
NA	Shrub Cover, closed-open, evergreen		Marine or coastal wetlands, J: Coastal brackish / saline lagoons, H: Intertidal marshes, F: Estuarine waters, E: Sand, shingle or pebble shores, G: Intertidal mud, sand or salt flats, Inland wetlands, Tp: Permanent freshwater marshes/ pools	0	11	0	J	80
NA	Shrub Cover, closed-open, evergreen	Lake		0	11	1	0	41
NA	Shrub Cover, closed-open, evergreen	Coastal Wetland		0	11	5	0	80
NA	Shrub Cover, closed-open, deciduous			0	12	0	0	59
NA	Shrub Cover, closed-open, deciduous		Marine or coastal wetlands, J: Coastal brackish / saline lagoons, H: Intertidal marshes, F: Estuarine waters, E: Sand, shingle or pebble shores, G: Intertidal mud, sand or salt flats, Inland wetlands, Tp: Permanent freshwater marshes/ pools	0	12	0	J	80

NA	Shrub Cover, closed-open, deciduous	Lake		0	12	1	0	41
NA	Herbaceous Cover, closed-open			0	13	0	0	56
NA	Herbaceous Cover, closed-open		Marine or coastal wetlands, H: Intertidal marshes, J: Coastal brackish / saline lagoons, Human-made wetlands, 9: Canals and drainage channels or ditches	0	13	0	H	80
NA	Herbaceous Cover, closed-open		Marine or coastal wetlands, J: Coastal brackish / saline lagoons, H: Intertidal marshes, F: Estuarine waters, E: Sand, shingle or pebble shores, G: Intertidal mud, sand or salt flats, Inland wetlands, Tp: Permanent freshwater marshes/ pools	0	13	0	J	80
NA	Herbaceous Cover, closed-open	Coastal Wetland		0	13	5	0	80
NA	Regularly flooded shrub and/or herbaceous cover			0	15	0	0	57

NA	Regularly flooded shrub and/or herbaceous cover	Lake		0	15	1	0	60
NA	Regularly flooded shrub and/or herbaceous cover	Coastal Wetland		0	15	5	0	80
	Cultivated and managed areas			0	16	0	0	74
NA	Cultivated and managed areas			0	16	0	0	74
NA	Cultivated and managed areas		Marine or coastal wetlands, E: Sand, shingle or pebble shores, F: Estuarine waters, Inland wetlands, M: Permanent rivers/ streams/ creeks, O: Permanent freshwater lakes, P: Seasonal/ intermittent freshwater lakes, Tp: Permanent freshwater marshes/ pools,	0	16	0	E	80

NA	Cultivated and managed areas		Marine or coastal wetlands, J: Coastal brackish / saline lagoons, H: Intertidal marshes, F: Estuarine waters, E: Sand, shingle or pebble shores, G: Intertidal mud, sand or salt flats, Inland wetlands, Tp: Permanent freshwater marshes/ pools	0	16	0	J	80
NA	Cultivated and managed areas	Lake		0	16	1	0	41
NA	Cultivated and managed areas	Coastal Wetland		0	16	5	0	80
NA	Cultivated and managed areas	Coastal Wetland	Marine or coastal wetlands, E: Sand, shingle or pebble shores, F: Estuarine waters, Inland wetlands, M: Permanent rivers/ streams/ creeks, O: Permanent freshwater lakes, P: Seasonal/ intermittent freshwater lakes, Tp: Permanent freshwater marshes/ pools,	0	16	5	E	80
NA	Mosaic: Cropland / Shrub and/or grass cover			0	18	0	0	74
NA	Bare Areas			0	19	0	0	79
NA	Bare Areas	Lake		0	19	1	0	41
NA	Bare Areas	Coastal Wetland		0	19	5	0	80

NA	Water Bodies		Human-made wetlands, 3: Irrigated land, Marine or coastal wetlands, E: Sand, shingle or pebble shores, J: Coastal brackish / saline lagoons, Inland wetlands, Ts: Seasonal/ intermittent freshwater marshes/ pools on inorganic soils	0	20	0	3	81
NA	Water Bodies		Marine or coastal wetlands, B: Marine subtidal aquatic beds (Underwater vegetation), K: Coastal freshwater lagoons, J: Coastal brackish / saline lagoons, H: Intertidal marshes, E: Sand, shingle or pebble shores, A: Permanent shallow marine waters, F: Est	0	20	0	B	80
NA	Water Bodies		Marine or coastal wetlands, E: Sand, shingle or pebble shores, F: Estuarine waters, Inland wetlands, M: Permanent rivers/ streams/ creeks, O: Permanent freshwater lakes, P: Seasonal/ intermittent freshwater lakes, Tp: Permanent freshwater marshes/ pools,	0	20	0	E	80

NA	Water Bodies		Marine or coastal wetlands, H: Intertidal marshes, G: Intertidal mud, sand or salt flats, E: Sand, shingle or pebble shores, F: Estuarine waters, J: Coastal brackish / saline lagoons, Inland wetlands, M: Permanent rivers/ streams/ creeks	0	20	0	H	80
NA	Water Bodies		Marine or coastal wetlands, J: Coastal brackish / saline lagoons, H: Intertidal marshes, F: Estuarine waters, E: Sand, shingle or pebble shores, G: Intertidal mud, sand or salt flats, Inland wetlands, Tp: Permanent freshwater marshes/ pools	0	20	0	J	80
NA	Water Bodies		Inland wetlands, Q: Permanent saline/ brackish/ alkaline lakes, M: Permanent rivers/ streams/ creeks, Human-made wetlands, 5: Salt exploitation sites	0	20	0	Q	44

NA	Water Bodies		Inland wetlands, Sp: Permanent saline/ brackish/ alkaline marshes/ pools, Marine or coastal wetlands, K: Coastal freshwater lagoons, J: Coastal brackish / saline lagoons, Human-made wetlands, 9: Canals and drainage channels or ditches	0	20	0	Sp	52
NA	Water Bodies		Inland wetlands, Ss: Seasonal/ intermittent saline/ brackish/ alkaline marshes/ pools, Q: Permanent saline/ brackish/ alkaline lakes, R: Seasonal/ intermittent saline/ brackish/ alkaline lakes and flats, Sp: Permanent saline/ brackish/ alkaline marshes/	0	20	0	Ss	52
NA	Water Bodies	Lake		0	20	1	0	41

NA	Water Bodies	Lake	Marine or coastal wetlands, E: Sand, shingle or pebble shores, F: Estuarine waters, Inland wetlands, M: Permanent rivers/ streams/ creeks, O: Permanent freshwater lakes, P: Seasonal/ intermittent freshwater lakes, Tp: Permanent freshwater marshes/ pools,	0	20	1	E	80
NA	Water Bodies	Lake	Marine or coastal wetlands, H: Intertidal marshes, J: Coastal brackish / saline lagoons, Human-made wetlands, 9: Canals and drainage channels or ditches	0	20	1	H	80
NA	Water Bodies	River		0	20	3	0	46
NA	Water Bodies	River	Marine or coastal wetlands, E: Sand, shingle or pebble shores, F: Estuarine waters, Inland wetlands, M: Permanent rivers/ streams/ creeks, O: Permanent freshwater lakes, P: Seasonal/ intermittent freshwater lakes, Tp: Permanent freshwater marshes/ pools,	0	20	3	E	80
NA	Water Bodies	Coastal Wetland		0	20	5	0	80

NA	Water Bodies	Coastal Wetland	Marine or coastal wetlands, B: Marine subtidal aquatic beds (Underwater vegetation), K: Coastal freshwater lagoons, J: Coastal brackish / saline lagoons, H: Intertidal marshes, E: Sand, shingle or pebble shores, A: Permanent shallow marine waters, F: Est	0	20	5	B	80
NA	Water Bodies	Coastal Wetland	Marine or coastal wetlands, E: Sand, shingle or pebble shores, F: Estuarine waters, Inland wetlands, M: Permanent rivers/ streams/ creeks, O: Permanent freshwater lakes, P: Seasonal/ intermittent freshwater lakes, Tp: Permanent freshwater marshes/ pools,	0	20	5	E	80
NA	Water Bodies	Coastal Wetland	Inland wetlands, Ss: Seasonal/ intermittent saline/ brackish/ alkaline marshes/ pools, Q: Permanent saline/ brackish/ alkaline lakes, R: Seasonal/ intermittent saline/ brackish/ alkaline lakes and flats, Sp: Permanent saline/ brackish/ alkaline marshes/	0	20	5	Ss	52

NA	Snow and Ice			0	21	0	0	79
NA	Artificial surfaces and associated areas			0	22	0	0	73
NA	Artificial surfaces and associated areas		Marine or coastal wetlands, H: Intertidal marshes, G: Intertidal mud, sand or salt flats, E: Sand, shingle or pebble shores, F: Estuarine waters, J: Coastal brackish / saline lagoons, Inland wetlands, M: Permanent rivers/ streams/ creeks	0	22	0	H	80
NA	Artificial surfaces and associated areas	Lake		0	22	1	0	41
NA	Artificial surfaces and associated areas	Coastal Wetland		0	22	5	0	80
NA	Artificial surfaces and associated areas	Coastal Wetland	Marine or coastal wetlands, B: Marine subtidal aquatic beds (Underwater vegetation), K: Coastal freshwater lagoons, J: Coastal brackish / saline lagoons, H: Intertidal marshes, E: Sand, shingle or pebble shores, A: Permanent shallow marine waters, F: Est	0	22	5	B	80
Extensive Grassland				1	0	0	0	75

Intensive Grassland				2	0	0	0	76
Silage				3	0	0	0	76
Forage				4	0	0	0	76
Hop				5	0	0	0	74
Legumes				6	0	0	0	74
Maize				7	0	0	0	74
Oat				8	0	0	0	74
Oleaginous/Rapseed				9	0	0	0	74
Potatoes				10	0	0	0	74
Rye				11	0	0	0	74
Setaside				12	0	0	0	74
Sugar Beet				13	0	0	0	74
Summer Barley				14	0	0	0	74
Summer Wheat				15	0	0	0	74
Winter Barley				16	0	0	0	74
Winter Wheat				17	0	0	0	74
Industrial built up				18	0	0	0	73
Residential built up				19	0	0	0	73
Deciduous Forest	Cultivated and managed areas			20	16	0	0	62
Deciduous Forest	Mosaic: Cropland / Tree Cover / Other natural vegetation			20	17	0	0	62
Deciduous Forest	Water Bodies			20	20	0	0	62

Deciduous Forest	Artificial surfaces and associated areas			20	22	0	0	62
Deciduous Forest	Tree Cover broadleaved, deciduous closed			20	2	0	0	62
Deciduous Forest	Tree Cover, needle leaved, evergreen			20	4	0		62
Deciduous Forest	Tree Cover, needle leaved, evergreen		1	20	4	0	1	62
Deciduous Forest	Tree Cover, needle leaved, evergreen		4	20	4	0	4	81
Deciduous Forest	Tree Cover, needle leaved, evergreen		6	20	4	0	6	78
Deciduous Forest	Tree Cover, needle leaved, evergreen		7	20	4	0	7	78
Deciduous Forest	Tree Cover, needle leaved, evergreen		9	20	4	0	9	78
Deciduous Forest	Tree Cover, needle leaved, evergreen		M	20	4	0	M	47
Deciduous Forest	Tree Cover, needle leaved, evergreen		O	20	4	0	O	42

Deciduous Forest	Tree Cover, needle leaved, evergreen		Tp	20	4	0	Tp	51
Deciduous Forest	Tree Cover, needle leaved, evergreen		Ts	20	4	0	Ts	53
Deciduous Forest	Tree Cover, needle leaved, evergreen		U	20	4	0	U	55
Deciduous Forest	Tree Cover, needle leaved, evergreen		W	20	4	0	W	60
Deciduous Forest	Tree Cover, needle leaved, evergreen		Xf	20	4	0	Xf	63
Deciduous Forest	Tree Cover, needle leaved, evergreen		Xp	20	4	0	Xp	63
Deciduous Forest	Tree Cover, needle leaved, evergreen		Y	20	4	0	Y	48
Deciduous Forest	Tree Cover, needle leaved, evergreen		Zk(b)	20	4	0	Zk(b)	77
Deciduous Forest	Tree Cover, mixed leaf type			20	6	0	0	68
Deciduous Forest	Tree Cover, mixed leaf type		Human-made wetlands, 1: Aquaculture	20	6	0	1	78
Deciduous Forest	Tree Cover, mixed leaf type		Human-made wetlands, 4: Seasonally flooded agricultural land	20	6	0	4	81
Deciduous Forest	Tree Cover, mixed leaf type		Human-made wetlands, 6: Water storage areas	20	6	0	6	78

Deciduous Forest	Tree Cover, mixed leaf type		Human-made wetlands, 7: Excavations	20	6	0	7	78
Deciduous Forest	Tree Cover, mixed leaf type		Inland wetlands, L: Permanent inland deltas	20	6	0	L	63
Deciduous Forest	Tree Cover, mixed leaf type		Inland wetlands, M: Permanent rivers/ streams/ creeks	20	6	0	M	63
Deciduous Forest	Tree Cover, mixed leaf type		Inland wetlands, O: Permanent freshwater lakes	20	6	0	O	42
Deciduous Forest	Tree Cover, mixed leaf type		Inland weltands, Tp: Permanent freshwater marshes/ pools	20	6	0	Tp	51
Deciduous Forest	Tree Cover, mixed leaf type		Inland wetlands, Ts: Seasonal/intermittent freshwater marshes/pools on inorganic soils	20	6	0	Ts	53
Deciduous Forest	Tree Cover, mixed leaf type		Inland wetlands, U: Non-forested peatlands	20	6	0	U	63
Deciduous Forest	Tree Cover, mixed leaf type		Inland wetlands, W: Shrub-dominated wetlands	20	6	0	W	63
Deciduous Forest	Tree Cover, mixed leaf type		Inland wetlands, Xf: Freshwater, tree-dominated wetlands	20	6	0	Xf	63
Deciduous Forest	Tree Cover, mixed leaf type		Inland wetlands, Xp: Forested peatlands	20	6	0	Xp	63
Deciduous Forest	Tree Cover, mixed leaf type		Inland wetlands, Y: Freshwater springs; oases	20	6	0	Y	48
Deciduous Forest	Shrub Cover, closed-open, deciduous			20	12	0	0	59

Deciduous Forest	Shrub Cover, closed-open, deciduous			20	12	0	6	59
Deciduous Forest	Shrub Cover, closed-open, deciduous	Lake		20	12	1	0	41
Deciduous Forest	Herbaceous Cover, closed-open			20	13	0	0	62
Deciduous Forest	Herbaceous Cover, closed-open		1	20	13	0	1	62
Deciduous Forest	Herbaceous Cover, closed-open		Ts	20	13	0	Ts	53
Deciduous Forest	Herbaceous Cover, closed-open	Lake		20	13	1	0	41
Deciduous Forest	Herbaceous Cover, closed-open	River		20	13	3	0	46
Deciduous Forest	Sparse herbaceous or sparse shrub cover			20	14	0	0	62
Deciduous Forest	Regularly flooded shrub and/or herbaceous cover			20	15	0	0	60

Deciduous Forest	Regularly flooded shrub and/or herbaceous cover	Lake		20	15	1	0	41
Deciduous Forest	Regularly flooded shrub and/or herbaceous cover	River		20	15	3	0	46
Deciduous Forest	Regularly flooded shrub and/or herbaceous cover	River	E	20	15	3	E	80
Deciduous Forest	Regularly flooded shrub and/or herbaceous cover	Coastal Wetland		20	15	5	0	80
Deciduous Forest	Regularly flooded shrub and/or herbaceous cover	Coastal Wetland	1	20	15	5	1	78
Deciduous Forest	Regularly flooded shrub and/or herbaceous cover	Coastal Wetland	E	20	15	5	E	80

Deciduous Forest	Mosaic: Cropland / Shrub and/or grass cover			20	18	0	0	62
Deciduous Forest	Mosaic: Cropland / Shrub and/or grass cover	Reservoir		20	18	2	0	78
Conifer Forest	Tree Cover, broadleaved, deciduous, closed		Human-made wetlands, 6: Water storage areas/Reservoirs, 9: Canals and drainage channels or ditches, 1: Aquaculture ponds, 4: Seasonally flooded agricultural land, Inland wetlands, M: Permanent rivers/ streams/ creeks, Tp: Permanent freshwater marshes/ po	21	2	0	6	78
Conifer Forest	Shrub Cover, closed-open, deciduous	Lake		21	12	1	0	41
Conifer Forest	Herbaceous Cover, closed-open	Lake		21	13	1	0	71
Conifer Forest	Sparse herbaceous or sparse shrub cover			21	14	0	0	71

Conifer Forest	Cultivated and managed areas		Human-made wetlands, 6: Water storage areas/Reservoirs, 4: Seasonally flooded agricultural land, 7: Excavations, 9: Canals and drainage channels or ditches, 2: Ponds, Inland wetlands, Ts: Seasonal/ intermittent freshwater marshes/ pools on inorganic soil	21	16	0	6	78
Conifer Forest	Cultivated and managed areas		Human-made wetlands, 7: Excavations, 6: Water storage areas/Reservoirs, 4: Seasonally flooded agricultural land, 1: Aquaculture ponds, 9: Canals and drainage channels or ditches, Inland wetlands, Xf: Freshwater, tree-dominated wetlands, W: Shrub-dominate	21	16	0	7	78
Conifer Forest	Cultivated and managed areas	Lake		21	16	1	0	41
Conifer Forest	Cultivated and managed areas	River		21	16	3	0	46
Conifer Forest	Artificial surfaces and associated areas	Lake		21	22	1	0	41
Conifer Forest			Inland wetlands, Xf: Freshwater, tree-dominated wetlands	21	0	0	Xf	63

Conifer Forest			Inland wetlands, Xp: Forested peatlands	21	0	0	Xp	63
Conifer Forest	Tree Cover, broadleaved, deciduous, closed			21	2	0	0	66
Conifer Forest	Tree Cover, broadleaved, deciduous, closed		Human-made wetlands, 1: Aquaculture ponds, Inland wetlands, Xf: Freshwater, tree-dominated wetlands, U: Permanent Non-forested peatlands, W: Shrub-dominated wetlands	21	2	0	1	63
Conifer Forest	Tree Cover, broadleaved, deciduous, closed		Human-made wetlands, 4: Seasonally flooded agricultural land, 6: Water storage areas/Reservoirs, Inland wetlands, M: Permanent rivers/ streams/ creeks, Tp: Permanent freshwater marshes/ pools, Ts: Seasonal/ intermittent freshwater marshes/ pools on inorg	21	2	0	4	81

Conifer Forest	Tree Cover, broadleaved, deciduous, closed		Human-made wetlands, 9: Canals and drainage channels or ditches, 4: Seasonally flooded agricultural land, 2: Ponds, Inland wetlands, U: Permanent Non-forested peatlands, Tp: Permanent freshwater marshes/ pools, Xp: Permanent Forested peatlands	21	2	0	9	81
Conifer Forest	Tree Cover, broadleaved, deciduous, closed		Marine or coastal wetlands, H: Intertidal marshes, G: Intertidal mud, sand or salt flats, E: Sand, shingle or pebble shores, F: Estuarine waters, J: Coastal brackish / saline lagoons, Inland wetlands, M: Permanent rivers/ streams/ creeks	21	2	0	H	80
Conifer Forest	Tree Cover, broadleaved, deciduous, closed		Inland wetlands, M: Permanent rivers/ streams/ creeks, N: Seasonal/ intermittent/ irregular rivers/ streams/ creeks, O: Permanent freshwater lakes, Tp: Permanent freshwater marshes/ pools, Ts: Seasonal/ intermittent freshwater marshes/ pools on inorganic	21	2	0	M	63

~~Conifer Forest~~	~~Tree Cover, broadleaved, deciduous, closed~~		~~Inland wetlands, M: Permanent rivers/ streams/ creeks, O: Permanent freshwater lakes, Tp: Permanent freshwater marshes/ pools, W: Shrub dominated wetlands, Xf: Freshwater, tree-dominated wetlands, L: Permanent inland deltas, Human-made wetlands, 9: Canal~~	~~21~~	~~2~~	~~0~~	~~M~~	~~63~~
Conifer Forest	Tree Cover, broadleaved, deciduous, closed		Inland wetlands, N: Seasonal/intermittent/irregular rivers/ streams creeks	21	2	0	N	63
Conifer Forest	Tree Cover, broadleaved, deciduous, closed		Inland wetlands, O: Permanent freshwater lakes, Tp: Permanent freshwater marshes/ pools	21	2	0	O	42
Conifer Forest	Tree Cover, broadleaved, deciduous, closed		Inland wetlands, Ts: Seasonal/ intermittent freshwater marshes/ pools on inorganic soils, M: Permanent rivers/ streams/ creeks, W: Shrub-dominated wetlands, Xf: Freshwater, tree-dominated wetlands, P: Seasonal/ intermittent freshwater lakes, Zk(b): Karst	21	2	0	Ts	63

Conifer Forest	Tree Cover, broadleaved, deciduous, closed		Inland wetlands, Ts: Seasonal/ intermittent freshwater marshes/ pools on inorganic soils, M: Permanent rivers/ streams/ creeks, Xf: Freshwater, tree-dominated wetlands, Human-made wetlands, 1: Aquaculture ponds	21	2	0	Ts	63
Conifer Forest	Tree Cover, broadleaved, deciduous, closed		Inland wetlands, Xp: Forested peatlands	21	2	0	Xp	63
Conifer Forest	Tree Cover, broadleaved, deciduous, closed		Inland wetlands, Y: Permanent Freshwater springs; oases, M: Permanent rivers/ streams/ creeks, Tp: Permanent freshwater marshes/ pools, Xp: Permanent Forested peatlands, Xf: Freshwater, tree-dominated wetlands, U: Permanent Non-forested peatlands, W: Shr	21	2	0	Y	63
Conifer Forest	Tree Cover, broadleaved, deciduous, closed	Lake		21	2	1	0	66
Conifer Forest	Tree Cover, broadleaved, deciduous, closed	Freshwater Marsch, Floodplain		21	2	4	0	51

Conifer Forest	Tree Cover, broadleaved, deciduous, closed	Coastal Wetland		21	2	5	0	80
Conifer Forest	Tree Cover, needle leaved, evergreen			21	4	0	0	66
Conifer Forest	Tree Cover, needle leaved, deciduous			21	5	0	0	66
Conifer Forest	Tree Cover, mixed leaf type			21	6	0	0	68
Conifer Forest	Tree Cover regularly flooded, freshwater			21	7	0	0	63
Conifer Forest	Tree Cover regularly flooded, freshwater			~~21~~	~~7~~	~~0~~	~~0~~	~~67~~
Conifer Forest	Tree Cover regularly flooded, saline water			21	8	0	0	64
Conifer Forest	Mosaic: Tree Cover / Other natural vegetation			21	9	0	0	71
Conifer Forest	Tree Cover, burnt			21	10	0	0	70
Conifer Forest	Shrub Cover, closed-open, evergreen			21	11	0	0	59

Conifer Forest	Shrub Cover, closed-open, deciduous			21	12	0	0	59
Conifer Forest	Herbaceous Cover, closed-open			21	13	0	0	66
Conifer Forest	Herbaceous Cover, closed-open		Human-made wetlands, 3: Irrigated land, Marine or coastal wetlands, E: Sand, shingle or pebble shores, J: Coastal brackish / saline lagoons, Inland wetlands, Ts: Seasonal/ intermittent freshwater marshes/ pools on inorganic soils	21	13	0	3	81
Conifer Forest	Herbaceous Cover, closed-open		Inland wetlands, Y: Permanent Freshwater springs; oases, Zk(b): Karst and other subterranean hydrological systems, Va: Montane wetlands, M: Permanent rivers/ streams/ creeks, U: Permanent Non-forested peatlands, Human-made wetlands, 4: Seasonally flooded	21	13	0	Y	55

Conifer Forest	Herbaceous Cover, closed-open	Lake	Human-made wetlands, 3: Irrigated land, Marine or coastal wetlands, E: Sand, shingle or pebble shores, J: Coastal brackish / saline lagoons, Inland wetlands, Ts: Seasonal/ intermittent freshwater marshes/ pools on inorganic soils	21	13	1	3	81
Conifer Forest	Herbaceous Cover, closed-open	Coastal Wetland		21	13	5	0	80
Conifer Forest	Sparse herbaceous or sparse shrub cover		Inland wetlands, Y: Permanent Freshwater springs; oases, Zk(b): Karst and other subterranean hydrological systems, Va: Montane wetlands, M: Permanent rivers/ streams/ creeks, U: Permanent Non-forested peatlands, Human-made wetlands, 4: Seasonally flooded	21	14	0	Y	55
Conifer Forest	Sparse herbaceous or sparse shrub cover	Lake		21	14	1	0	41
Conifer Forest	Sparse herbaceous or sparse shrub cover	Bog, Fen, Mire (Peatland)		21	14	7	0	52

Conifer Forest	Regularly flooded shrub and/or herbaceous cover			21	15	0	0	60
Conifer Forest	Regularly flooded shrub and/or herbaceous cover	Lake		21	15	1	0	60
Conifer Forest	Regularly flooded shrub and/or herbaceous cover	Reservoir		21	15	2	0	60
Conifer Forest	Regularly flooded shrub and/or herbaceous cover	Coastal Wetland		21	15	5	0	80
Conifer Forest	Regularly flooded shrub and/or herbaceous cover	Coastal Wetland	Marine or coastal wetlands, E: Sand, shingle or pebble shores, F: Estuarine waters, Inland wetlands, M: Permanent rivers/ streams/ creeks, O: Permanent freshwater lakes, P: Seasonal/ intermittent freshwater lakes, Tp: Permanent freshwater marshes/ pools,	21	15	5	E/9	80

Conifer Forest	Regularly flooded shrub and/or herbaceous cover	Coastal Wetland	Inland wetlands, Ss: Seasonal/ intermittent saline/ brackish/ alkaline marshes/ pools, Q: Permanent saline/ brackish/ alkaline lakes, R: Seasonal/ intermittent saline/ brackish/ alkaline lakes and flats, Sp: Permanent saline/ brackish/ alkaline marshes/	21	15	5	Ss	45
Conifer Forest	Cultivated and managed areas			21	16	0	0	66
Conifer Forest	Cultivated and managed areas		Human-made wetlands, 4: Seasonally flooded agricultural land, 6: Water storage areas/Reservoirs, Inland wetlands, M: Permanent rivers/ streams/ creeks, Tp: Permanent freshwater marshes/ pools, Ts: Seasonal/ intermittent freshwater marshes/ pools on inorg	21	16	0	4	81

Conifer Forest	Cultivated and managed areas		Marine or coastal wetlands, G: Intertidal mud, sand or salt flats, K: Coastal freshwater lagoons, H: Intertidal marshes, B: Marine subtidal aquatic beds (Underwater vegetation), J: Coastal brackish / saline lagoons, Human-made wetlands, 9: Canals and dra	21	16	0	G	80
Conifer Forest	Cultivated and managed areas		Inland wetlands, M: Permanent rivers/ streams/creeks	21	16	0	M	63
Conifer Forest	Cultivated and managed areas		Inland wetlands, Tp: Permanent freshwater marshes/ pools, O: Permanent freshwater lakes	21	16	0	Tp	51
Conifer Forest	Cultivated and managed areas		Inland wetlands, Ts: Seasonal/ intermittent freshwater marshes/ pools on inorganic soils, M: Permanent rivers/ streams/ creeks, W: Shrub-dominated wetlands, Xf: Freshwater, tree-dominated wetlands, P: Seasonal/ intermittent freshwater lakes, Zk(b): Karst	21	16	0	Ts	53

Conifer Forest	Cultivated and managed areas		Inland wetlands, U: Permanent Non-forested peatlands, Ts: Seasonal/ intermittent freshwater marshes/ pools on inorganic soils, Xp: Permanent Forested peatlands, Tp: Permanent freshwater marshes/ pools, Human-made wetlands, 9: Canals and drainage channels	21	16	0	U	55
Conifer Forest	Cultivated and managed areas		Inland wetlands, Xf: Freshwater, tree-dominated wetlands	21	16	0	Xf	63
Conifer Forest	Cultivated and managed areas		Inland wetlands, Y: Permanent Freshwater springs; oases, M: Permanent rivers/ streams/ creeks, Tp: Permanent freshwater marshes/ pools, Xp: Permanent Forested peatlands, Xf: Freshwater, tree-dominated wetlands, U: Permanent Non-forested peatlands, W: Shr	21	16	0	Y	63
Conifer Forest	Cultivated and managed areas	Lake	Inland wetlands, Tp: Permanent freshwater marshes/ pools, O: Permanent freshwater lakes	21	16	1	Tp	51

Conifer Forest	Cultivated and managed areas	Freshwater Marsch, Floodplain		21	16	4	0	51
Conifer Forest	Cultivated and managed areas	Coastal Wetland		21	16	5	0	80
Conifer Forest	Cultivated and managed areas	Coastal Wetland	Marine or coastal wetlands, E: Sand, shingle or pebble shores, F: Estuarine waters, Inland wetlands, M: Permanent rivers/ streams/ creeks, O: Permanent freshwater lakes, P: Seasonal/ intermittent freshwater lakes, Tp: Permanent freshwater marshes/ pools,	21	16	5	E	80
Conifer Forest	Mosaic: Cropland / Tree Cover / Other natural vegetation			21	17	0	0	66
Conifer Forest	Mosaic: Cropland / Shrub and/or grass cover			21	18	0	0	66
Conifer Forest	Bare Areas			21	19	0	0	66

Conifer Forest	Bare Areas		Marine or coastal wetlands, A: Permanent shallow marine waters, E: Sand, shingle or pebble shores, F: Estuarine waters, G: Intertidal mud, sand or salt flats, H: Intertidal marshes, J: Coastal brackish / saline lagoons, Inland wetlands, M: Permanent rive	21	19	0	A	80
Conifer Forest	Water Bodies			21	20	0	0	66
Conifer Forest	Water Bodies		Human-made wetlands, 3: Irrigated land, Marine or coastal wetlands, E: Sand, shingle or pebble shores, J: Coastal brackish / saline lagoons, Inland wetlands, Ts: Seasonal/ intermittent freshwater marshes/ pools on inorganic soils	21	20	0	3	81
Conifer Forest	Water Bodies		Human-made wetlands, 4: Seasonally flooded agricultural land, 6: Water storage areas/Reservoirs, Inland wetlands, M: Permanent rivers/ streams/ creeks, Tp: Permanent freshwater marshes/ pools, Ts: Seasonal/ intermittent freshwater marshes/ pools on inorg	21	20	0	4	81

Conifer Forest	Water Bodies		Human-made wetlands, 5: Salt exploitation sites, Marine or coastal wetlands, A: Permanent shallow marine waters, G: Intertidal mud, sand or salt flats, J: Coastal brackish / saline lagoons	21	20	0	5	64
Conifer Forest	Water Bodies		Marine or coastal wetlands, A: Permanent shallow marine waters, E: Sand, shingle or pebble shores, F: Estuarine waters, G: Intertidal mud, sand or salt flats, H: Intertidal marshes, J: Coastal brackish / saline lagoons, Inland wetlands, M: Permanent rive	21	20	0	A	80
Conifer Forest	Water Bodies		Marine or coastal wetlands, G: Intertidal mud, sand or salt flats, J: Coastal brackish / saline lagoons, B: Marine subtidal aquatic beds (Underwater vegetation), H: Intertidal marshes, Human-made wetlands, 9: Canals and drainage channels or ditches	21	20	0	G	80

Conifer Forest	Water Bodies		Inland wetlands, N: Seasonal/ intermittent/ irregular rivers/ streams/ creeks, M: Permanent rivers/ streams/ creeks, Human-made wetlands, 9: Canals and drainage channels or ditches, 4: Seasonally flooded agricultural land, 7: Excavations, Marine or coast	21	20	0	N	49
Conifer Forest	Water Bodies		Inland wetlands, O: Permanent freshwater lakes, Tp: Permanent freshwater marshes/ pools	21	20	0	O	42
Conifer Forest	Water Bodies		Inland wetlands, Sp: Permanent saline/ brackish/ alkaline marshes/ pools, Marine or coastal wetlands, K: Coastal freshwater lagoons, J: Coastal brackish / saline lagoons, Human-made wetlands, 9: Canals and drainage channels or ditches	21	20	0	Sp	52

Conifer Forest	Water Bodies		Inland wetlands, Ss: Seasonal/ intermittent saline/ brackish/ alkaline marshes/ pools, Q: Permanent saline/ brackish/ alkaline lakes, R: Seasonal/ intermittent saline/ brackish/ alkaline lakes and flats, Sp: Permanent saline/ brackish/ alkaline marshes/	21	20	0	Ss	45
Conifer Forest	Water Bodies		Inland wetlands, Y: Permanent Freshwater springs; oases, N: Seasonal/ intermittent/ irregular rivers/ streams/ creeks, Ts: Seasonal/ intermittent freshwater marshes/ pools on inorganic soils, Q: Permanent saline/ brackish/ alkaline lakes, Sp: Permanent s	21	20	0	Y	51
Conifer Forest	Water Bodies	Lake		21	20	1	0	41
Conifer Forest	Water Bodies	Lake	Human-made wetlands, 3: Irrigated land, Marine or coastal wetlands, E: Sand, shingle or pebble shores, J: Coastal brackish / saline lagoons, Inland wetlands, Ts: Seasonal/ intermittent freshwater marshes/ pools on inorganic soils	21	20	1	3	81

Conifer Forest	Water Bodies	Lake	Human-made wetlands, 4: Seasonally flooded agricultural land, 6: Water storage areas/Reservoirs, Inland wetlands, M: Permanent rivers/ streams/ creeks, Tp: Permanent freshwater marshes/ pools, Ts: Seasonal/ intermittent freshwater marshes/ pools on inorg	21	20	1	4	81
Conifer Forest	Water Bodies	Lake	Inland wetlands, O: Permanent freshwater lakes, Tp: Permanent freshwater marshes/ pools	21	20	1	O	42
Conifer Forest	Water Bodies	Lake	Inland wetlands, Tp: Permanent freshwater marshes/ pools, O: Permanent freshwater lakes	21	20	1	Tp	51
Conifer Forest	Water Bodies	Lake	Inland wetlands, U: Permanent Non-forested peatlands, M: Permanent rivers/ streams/ creeks, O: Permanent freshwater lakes, Tp: Permanent freshwater marshes/ pools	21	20	1	U	55

Conifer Forest	Water Bodies	Lake	Inland wetlands, Y: Permanent Freshwater springs; oases, N: Seasonal/ intermittent/ irregular rivers/ streams/ creeks, Ts: Seasonal/ intermittent freshwater marshes/ pools on inorganic soils, Q: Permanent saline/ brackish/ alkaline lakes, Sp: Permanent s	21	20	1	Y	42
Conifer Forest	Water Bodies	River		21	20	3	0	46
Conifer Forest	Water Bodies	Freshwater Marsch, Floodplain		21	20	4	0	51
Conifer Forest	Water Bodies	Coastal Wetland		21	20	5	0	80
Conifer Forest	Water Bodies	Coastal Wetland	Marine or coastal wetlands, E: Sand, shingle or pebble shores, F: Estuarine waters, Inland wetlands, M: Permanent rivers/ streams/ creeks, O: Permanent freshwater lakes, P: Seasonal/ intermittent freshwater lakes, Tp: Permanent freshwater marshes/ pools,	21	20	5	E	80

Conifer Forest	Water Bodies	Coastal Wetland	Inland wetlands, Ss: Seasonal/ intermittent saline/ brackish/ alkaline marshes/ pools, Q: Permanent saline/ brackish/ alkaline lakes, R: Seasonal/ intermittent saline/ brackish/ alkaline lakes and flats, Sp: Permanent saline/ brackish/ alkaline marshes/	21	20	5	Ss	45
Conifer Forest	Water Bodies	Bog, Fen, Mire (Peatland)		21	20	8	0	52
Conifer Forest	Snow and Ice			21	21	0	0	66
Conifer Forest	Artificial surfaces and associated areas			21	22	0	0	66
Conifer Forest	Artificial surfaces and associated areas		Human-made wetlands, 3: Irrigated land, Marine or coastal wetlands, E: Sand, shingle or pebble shores, J: Coastal brackish / saline lagoons, Inland wetlands, Ts: Seasonal/ intermittent freshwater marshes/ pools on inorganic soils	21	22	0	3	81

Conifer Forest	Artificial surfaces and associated areas		Marine or coastal wetlands, G: Intertidal mud, sand or salt flats, J: Coastal brackish / saline lagoons, B: Marine subtidal aquatic beds (Underwater vegetation), H: Intertidal marshes, Human-made wetlands, 9: Canals and drainage channels or ditches	21	22	0	G	80
Conifer Forest	Artificial surfaces and associated areas		Marine or coastal wetlands, H: Intertidal marshes, G: Intertidal mud, sand or salt flats, E: Sand, shingle or pebble shores, F: Estuarine waters, J: Coastal brackish / saline lagoons, Inland wetlands, M: Permanent rivers/ streams/ creeks	21	22	0	H	80
Conifer Forest	Artificial surfaces and associated areas	Lake	Human-made wetlands, 3: Irrigated land, Marine or coastal wetlands, E: Sand, shingle or pebble shores, J: Coastal brackish / saline lagoons, Inland wetlands, Ts: Seasonal/ intermittent freshwater marshes/ pools on inorganic soils	21	22	1	3	81
Wetland	Tree Cover, mixed leaf type			23	6	0	0	63
Wetland	Tree Cover, mixed leaf type			23	6	0	1	63

Wetland	Tree Cover, mixed leaf type			23	6	0	4	63
Wetland	Tree Cover, mixed leaf type			23	6	0	M	63
Wetland	Tree Cover, mixed leaf type			23	6	0	Xf	63
Wetland	Tree Cover, mixed leaf type			23	6	0	U	55
Wetland	Tree Cover, mixed leaf type			23	6	0	W	60
Wetland	Shrub Cover, closed-open, deciduous			23	12	0	0	60
Wetland	Shrub Cover, closed-open, deciduous			23	12	0	6	60
Wetland	Regularly flooded shrub and/or herbaceous cover			23	15	0	0	60
Wetland	Regularly flooded shrub and/or herbaceous cover			23	15	0	9	60
Wetland	Regularly flooded shrub and/or herbaceous cover			23	15	0	E	60

Alpine	Herbaceous Cover, closed-open			24	13	0	0	58
Alpine	Herbaceous Cover, closed-open			24	13	0	M	54
Natural Grass	Tree Cover broadleaved			25	2	0	0	56
Natural Grass	Tree Cover broadleaved		Human-made wetlands, 1: Aquaculture	25	2	0	1	78
Natural Grass	Tree Cover broadleaved		Human-made wetlands, 4: Seasonally flooded agricultural land	25	2	0	4	81
Natural Grass	Tree Cover broadleaved		Human-made wetlands, 6: Water storage areas	25	2	0	6	78
Natural Grass	Tree Cover broadleaved		Inland wetlands, M: Permanent rivers/ streams/ creeks	25	2	0	M	47
Natural Grass	Tree Cover broadleaved		Inland wetlands, W: Shrub-dominated wetlands	25	2	0	W	60
Natural Grass	Tree Cover broadleaved		Inland wetlands, Xf: Freshwater, tree-dominated wetlands	25	2	0	Xf	63
Natural Grass	Tree Cover broadleaved		Inland wetlands, Y: Freshwater springs; oases	25	2	0	Y	48
Natural Grass	Tree Cover broadleaved	Lake		25	2	1	0	41
Natural Grass	Tree Cover broadleaved	Reservoir		25	2	2	0	78
Natural Grass	Tree Cover broadleaved	River		25	2	3	0	46

Natural Grass	Tree Cover broadleaved	Freshwater Marsch, Floodplain		25	2	4	0	51
Natural Grass	Tree Cover, needle-leaved, evergreen			25	4	0	0	56
Natural Grass	Tree Cover, needle-leaved, evergreen		Human-made wetlands, 4: Seasonally flooded agricultural land	25	4	0	4	81
Natural Grass	Tree Cover, needle-leaved, evergreen		Human-made wetlands, 7: Excavations	25	4	0	7	78
Natural Grass	Tree Cover, needle-leaved, evergreen		Inland wetlands, M: Permanent rivers/ streams/ creeks	25	4	0	M	47
Natural Grass	Tree Cover, needle-leaved, evergreen		Inland wetlands, O: Permanent freshwater lakes	25	4	0	O	42
Natural Grass	Tree Cover, needle-leaved, evergreen		Inland wetlands, Tp: Permanent freshwater marshes/ pools	25	4	0	Tp	51
Natural Grass	Tree Cover, needle-leaved, evergreen		Inland wetlands, Ts: Seasonal/ intermittent freshwater marshes/ pools on inorganic soils	25	4	0	Ts	53
Natural Grass	Tree Cover, needle-leaved, evergreen		Inland wetlands, U: Non-forested peatlands	25	4	0	U	55
Natural Grass	Tree Cover, needle-leaved, evergreen		Inland wetlands, W: Shrub-dominated wetlands	25	4	0	W	60

Natural Grass	Tree Cover, needle-leaved, evergreen		Inland wetlands, Xf: Freshwater, tree-dominated wetlands	25	4	0	Xf	63
Natural Grass	Tree Cover, needle-leaved, evergreen		Inland wetlands, Y: Freshwater springs; oases	25	4	0	Y	48
Natural Grass	Tree Cover, needle-leaved, evergreen	Lake		25	4	1	0	41
Natural Grass	Tree Cover, needle-leaved, evergreen	Lake	Inland wetlands, Tp: Permanent freshwater marshes/ pools	25	4	1	Tp	51
Natural Grass	Tree Cover, needle-leaved, evergreen	Lake	Inland wetlands, U: Non-forested peatlands	25	4	1	U	55
Natural Grass	Tree Cover, needle-leaved, evergreen	Lake	Inland wetlands, Xf: Freshwater, tree-dominated wetlands	25	4	1	Xf	63
Natural Grass	Tree Cover, needle-leaved, evergreen	River		25	4	3	0	46
Natural Grass	Tree Cover, needle-leaved, evergreen	River	Inland wetlands, Ts: Seasonal/ intermittent freshwater marshes/ pools on inorganic soils	25	4	3	Ts	53
Natural Grass	Tree Cover, mixed leaf type			25	6	0	0	56
Natural Grass	Tree Cover, mixed leaf type		Inland wetlands, Ts: Seasonal/ intermittent freshwater marshes/ pools on inorganic soils	25	6	0	Ts	53
Natural Grass	Tree Cover, mixed leaf type		Inland wetlands, U: Non-forested peatlands	25	6	0	U	55

Natural Grass	Tree Cover, mixed leaf type		Inland wetlands, W: Shrub-dominated wetlands	25	6	0	W	60
Natural Grass	Tree Cover, mixed leaf type		Inland wetlands, Xf: Freshwater, tree-dominated wetlands	25	6	0	Xf	63
Natural Grass	Tree Cover, mixed leaf type	Lake		25	6	1	0	41
Natural Grass	Tree Cover, mixed leaf type	River		25	6	3	0	46
Natural Grass	Tree Cover, mixed leaf type	Freshwater Marsch, Floodplain		25	6	4	0	51
Natural Grass	Shrub Cover, closed-open, deciduous			25	12	0	0	59
Natural Grass	Herbaceous Cover, closed-open			25	13	0	0	56
Natural Grass	Herbaceous Cover, closed-open		Inland wetlands, M: Permanent rivers/ streams/ creeks	25	13	0	M	47
Natural Grass	Herbaceous Cover, closed-open		Inland wetlands, R: Seasonal/intermittent saline/brackish/alkaline lakes and flats	25	13	0	R	45
Natural Grass	Herbaceous Cover, closed-open		Inland wetlands, W: Shrub-dominated wetlands	25	13	0	W	60
Natural Grass	Herbaceous Cover, closed-open	Lake		25	13	1	0	41

Natural Grass	Herbaceous Cover, closed-open	Pan, Brakish/Saline Wetland		25	13	6	Ss	52
Natural Grass	Herbaceous Cover, closed-open	Bog, Fen, Mire (Peatland)		25	13	7	0	55
Natural Grass	Sparse herbaceous or sparse shrub cover			25	14	0	0	79
Natural Grass	Sparse herbaceous or sparse shrub cover			25	14	0	O/Tp	57
Natural Grass	Regularly flooded shrub and/or herbaceous cover			25	15	0	0	57
Natural Grass	Cultivated and managed areas	Lake		25	16	1	0	
Natural Grass	Cultivated and managed areas	Lake	Human-made wetlands, E:	25	16	1	E	80
Natural Grass	Cultivated and managed areas	Lake	Inland wetlands, Sp:	25	16	1	Sp	52
Natural Grass	Cultivated and managed areas	Lake	Inland wetlands, Tp:	25	16	1	Tp	51
Natural Grass	Cultivated and managed areas	Lake	Inland wetlands, R: Seasonal/intermittent saline/brackish/alkaline lakes and flats	25	16	1	R	45
Natural Grass	Cultivated and managed areas	Lake	Inland wetlands, Ss:	25	16	1	Ss	52

Natural Grass	Cultivated and managed areas	Lake	Human-made wetlands, 1	25	16	1	1	78
Natural Grass	Cultivated and managed areas	River		25	16	3	0	46
Natural Grass	Cultivated and managed areas	River	Human-made wetlands, 6:	25	16	3	6	78
Natural Grass	Cultivated and managed areas	River	Human-made wetlands, 7:	25	16	3	7	78
Natural Grass	Cultivated and managed areas	Freshwater Marsch, Floodplain		25	16	4	0	51
Natural Grass	Cultivated and managed areas	Coastal Wetland		25	16	5	0	80
Natural Grass	Cultivated and managed areas	Pan, Brakish/Saline Wetland	Marine/ Coastal wetlands, E:	25	16	5	E	80
Natural Grass	Cultivated and managed areas	Pan, Brakish/Saline Wetland		25	16	6	0	52
Natural Grass	Cultivated and managed areas	Pan, Brakish/Saline Wetland	Inland wetlands, W:	25	16	6	W	52
Natural Grass	Cultivated and managed areas	Pan, Brakish/Saline Wetland	Inland wetlands, Ss:	25	16	6	Ss	52
Natural Grass	Mosaic: Cropland / Tree Cover / Other natural vegetation			25	17	0	0	56

Natural Grass	Mosaic: Cropland / Shrub and/or grass cover			25	18	0	0	56
Natural Grass	Water Bodies			25	20	0	0	56
Natural Grass	Water Bodies		Xf	25	20	0	Xf	63
Natural Grass	Water Bodies		Tp	25	20	0	Tp	51
Natural Grass	Water Bodies		4	25	20	0	4	81
Natural Grass	Water Bodies	Lake		25	20	1	0	41
Natural Grass	Water Bodies	River		25	20	3	0	46
Natural Grass	Water Bodies	Freshwater Marsch, Floodplain		25	20	4	0	51
Natural Grass	Water Bodies	Coastal Wetland		25	20	5	0	80
Natural Grass	Water Bodies	Coastal Wetland	E	25	20	5	E	80
Natural Grass	Snow and Ice			25	21	0	0	56
Natural Grass	Tree Cover, mixed leaf type		Human-made wetlands, 7: Excavations	25	6	0	7	78
Water	Tree Cover, broadleaved, deciduous, closed			27	2	0	0	63

Water	Tree Cover, broadleaved, deciduous, closed		Human-made wetlands, 1: Aquaculture ponds, Inland wetlands, Xf: Freshwater, tree-dominated wetlands, Ts: Seasonal/ intermittent freshwater marshes/ pools on inorganic soils, Tp: Permanent freshwater marshes/ pools, O: Permanent freshwater lakes, M: Perma	27	2	0	1	63
Water	Tree Cover, broadleaved, deciduous, closed		Human-made wetlands, 6: Water storage areas	27	2	0	6	63
Water	Tree Cover, broadleaved, deciduous, closed		Inland wetlands, M: Permanent rivers/ streams/ creeks	27	2	0	M	47
Water	Tree Cover, broadleaved, deciduous, closed		Inland wetlands, N: Seasonal/intermittent/irregular rivers/ streams creeks	27	2	0	N	49

Water	Tree Cover, broadleaved, deciduous, closed		Inland wetlands, Ts: Seasonal/ intermittent freshwater marshes/ pools on inorganic soils, W: Shrub-dominated wetlands, M: Permanent rivers/ streams/ creeks, Xf: Freshwater, tree-dominated wetlands, O: Permanent freshwater lakes, N: Seasonal/ intermittent	27	2	0	Ts	53
Water	Tree Cover, broadleaved, deciduous, closed		Inland wetlands, W: Shrub-dominated wetlands, Xf: Freshwater, tree-dominated wetlands, Tp: Permanent freshwater marshes/ pools, P: Seasonal/ intermittent freshwater lakes, O: Permanent freshwater lakes	27	2	0	W	60
Water	Tree Cover, broadleaved, deciduous, closed	Freshwater Marsch, Floodplain		27	2	4	0	51
Water	Tree Cover, broadleaved, deciduous, closed	Freshwater Marsch, Floodplain	Human-made wetlands, 6: Water storage areas	27	2	4	6	51
Water	Tree Cover, broadleaved, deciduous, closed	Coastal Wetland		27	2	5	0	80

Water	Tree Cover, broadleaved, deciduous, closed	Coastal Wetland	Marine or coastal wetlands, E: Sand, shingle or pebble shores, F: Estuarine waters, Inland wetlands, M: Permanent rivers/ streams/ creeks, O: Permanent freshwater lakes, P: Seasonal/ intermittent freshwater lakes, Tp: Permanent freshwater marshes/ pools,	27	2	5	E	80
Water	Tree Cover, needle-leaved, evergreen			27	4	0	0	63
Water	Tree Cover, needle-leaved, evergreen		Human-made wetlands, 1: Aquaculture ponds, Inland wetlands, Xf: Freshwater, tree-dominated wetlands, Ts: Seasonal/ intermittent freshwater marshes/ pools on inorganic soils, Tp: Permanent freshwater marshes/ pools, O: Permanent freshwater lakes, M: Perma	27	4	0	1	63

Water	Tree Cover, needle-leaved, evergreen		Human-made wetlands, 4: Seasonally flooded agricultural land, 6: Water storage areas/Reservoirs, Inland wetlands, M: Permanent rivers/ streams/ creeks, Tp: Permanent freshwater marshes/ pools, Ts: Seasonal/ intermittent freshwater marshes/ pools on inorg	27	4	0	4	81
Water	Tree Cover, needle-leaved, evergreen		Human-made wetlands, 6: Water storage areas	27	4	0	6	63
Water	Tree Cover, needle-leaved, evergreen		Human-made wetlands, 7: Excavations	27	4	0	7	78
Water	Tree Cover, needle-leaved, evergreen		Marine or coastal wetlands, E: Sand, shingle or pebble shores, F: Estuarine waters, Inland wetlands, M: Permanent rivers/ streams/ creeks, O: Permanent freshwater lakes, P: Seasonal/ intermittent freshwater lakes, Tp: Permanent freshwater marshes/ pools,	27	4	0	E	80
Water	Tree Cover, needle-leaved, evergreen		Inland wetlands, M: Permanent rivers/ streams/ creeks	27	4	0	M	47

Water	Tree Cover, needle-leaved, evergreen		Inland wetlands, O: Permanent freshwater lakes	27	4	0	O	42
Water	Tree Cover, needle-leaved, evergreen		Inland wetlands, Tp: Permanent freshwater marshes/ pools	27	4	0	Tp	51
Water	Tree Cover, needle-leaved, evergreen		Inland wetlands, Ts: Seasonal/ intermittent freshwater marshes/ pools on inorganic soils, M: Permanent rivers/ streams/ creeks, Xf: Freshwater, tree-dominated wetlands, Human-made wetlands, 1: Aquaculture ponds	27	4	0	Ts	53
Water	Tree Cover, needle-leaved, evergreen		Inland wetlands, U: Permanent Non-forested peatlands, M: Permanent rivers/ streams/ creeks, O: Permanent freshwater lakes, Tp: Permanent freshwater marshes/ pools	27	4	0	U	55
Water	Tree Cover, needle-leaved, evergreen		Inland wetlands, W: Shrub-dominated wetlands	27	4	0	W	60

Water	Tree Cover, needle-leaved, evergreen		Inland wetlands, Xf: Freshwater, tree-dominated wetlands, M: Permanent rivers/ streams/ creeks, Tp: Permanent freshwater marshes/ pools, U: Permanent Non-forested peatlands, Human-made wetlands, 6: Water storage areas/Reservoirs	27	4	0	Xf	63
Water	Tree Cover, needle-leaved, evergreen	Freshwater Marsch, Floodplain		27	4	4	0	51
Water	Tree Cover, needle-leaved, evergreen	Freshwater Marsch, Floodplain	Human-made wetlands, 6: Water storage areas	27	4	4	6	78
Water	Tree Cover, needle-leaved, evergreen	Freshwater Marsch, Floodplain	Inland wetlands, M: Permanent rivers/ streams/ creeks	27	4	4	M	47
Water	Tree Cover, needle-leaved, evergreen	Freshwater Marsch, Floodplain	Inland wetlands, Tp: Permanent freshwater marshes/ pools	27	4	4	Tp	51
Water	Tree Cover, needle-leaved, evergreen	Freshwater Marsch, Floodplain	Inland wetlands, Xf: Freshwater, tree-dominated wetlands	27	4	4	Xf	
Water	Tree Cover, needle-leaved, evergreen	Coastal Wetland		27	4	5	0	80

Water	Tree Cover, needle-leaved, evergreen	Coastal Wetland	Marine or coastal wetlands, E: Sand, shingle or pebble shores, F: Estuarine waters, Inland wetlands, M: Permanent rivers/ streams/ creeks, O: Permanent freshwater lakes, P: Seasonal/ intermittent freshwater lakes, Tp: Permanent freshwater marshes/ pools,	27	4	5	E	80
Water	Tree Cover, mixed leaf type			27	6	0	0	69
Water	Tree Cover, mixed leaf type		Human-made wetlands, 1: Aquaculture ponds, Inland wetlands, Xf: Freshwater, tree-dominated wetlands, Ts: Seasonal/ intermittent freshwater marshes/ pools on inorganic soils, Tp: Permanent freshwater marshes/ pools, O: Permanent freshwater lakes, M: Perma	27	6	0	1	78

Water	Tree Cover, mixed leaf type		Human-made wetlands, 4: Seasonally flooded agricultural land, 7: Excavations, 9: Canals and drainage channels or ditches, Inland wetlands, M: Permanent rivers/ streams/ creeks, O: Permanent freshwater lakes, P: Seasonal/ intermittent freshwater lakes, Ts	27	6	0	4	81
Water	Tree Cover, mixed leaf type		Human-made wetlands, 7: Excavations	27	6	0	7	78
Water	Tree Cover, mixed leaf type		Inland wetlands, M: Permanent rivers/ streams/ creeks	27	6	0	M	47
Water	Tree Cover, mixed leaf type		Inland wetlands, Ts: Seasonal/ intermittent freshwater marshes/ pools on inorganic soils, M: Permanent rivers/ streams/ creeks, Xf: Freshwater, tree-dominated wetlands, Human-made wetlands, 1: Aquaculture ponds	27	6	0	Ts	53

Water	Tree Cover, mixed leaf type		Inland wetlands, W: Shrub-dominated wetlands, Xf: Freshwater, tree-dominated wetlands, Tp: Permanent freshwater marshes/ pools, P: Seasonal/ intermittent freshwater lakes, O: Permanent freshwater lakes	27	6	0	W	60
Water	Tree Cover, mixed leaf type		Inland wetlands, Xf: Freshwater, tree-dominated wetlands, M: Permanent rivers/ streams/ creeks, Tp: Permanent freshwater marshes/ pools, U: Permanent Non-forested peatlands, Human-made wetlands, 6: Water storage areas/Reservoirs	27	6	0	Xf	63
Water	Tree Cover, mixed leaf type	Freshwater Marsch, Floodplain		27	6	4	0	51
Water	Tree Cover, mixed leaf type	Freshwater Marsch, Floodplain	Human-made wetlands, 6: Water storage areas	27	6	4	6	51
Water	Tree Cover, mixed leaf type	Coastal Wetland		27	6	5	0	80

Water	Tree Cover, mixed leaf type	Coastal Wetland	Marine or coastal wetlands, E: Sand, shingle or pebble shores, F: Estuarine waters, Inland wetlands, M: Permanent rivers/ streams/ creeks, O: Permanent freshwater lakes, P: Seasonal/ intermittent freshwater lakes, Tp: Permanent freshwater marshes/ pools,	27	6	5	E	80
Water	Shrub Cover, closed-open, deciduous			27	12	0	0	60
Water	Herbaceous Cover, closed-open			27	13	0	0	57
Water	Herbaceous Cover, closed-open		Human-made wetlands, 1: Aquaculture ponds, Inland wetlands, Xf: Freshwater, tree-dominated wetlands, Ts: Seasonal/ intermittent freshwater marshes/ pools on inorganic soils, Tp: Permanent freshwater marshes/ pools, O: Permanent freshwater lakes, M: Perma	27	13	0	1	63
Water	Herbaceous Cover, closed-open		Inland wetlands, Tp: Permanent freshwater marshes/ pools	27	13	0	Tp	51

Water	Herbaceous Cover, closed-open		Inland wetlands, U: Non-forested peatlands	27	13	0	U	
Water	Herbaceous Cover, closed-open		Inland wetlands, W: Shrub-dominated wetlands, Xf: Freshwater, tree-dominated wetlands, Tp: Permanent freshwater marshes/ pools, P: Seasonal/ intermittent freshwater lakes, O: Permanent freshwater lakes	27	13	0	W	60
Water	Sparse herbaceous or sparse shrub cover			27	14	0	0	51
Water	Sparse herbaceous or sparse shrub cover		Inland wetlands, M: Permanent rivers/streams/creeks	27	14	0	M	47
Water	Regularly flooded shrub and/or herbaceous cover			27	15	0	0	60

Water	Regularly flooded shrub and/or herbaceous cover		Marine or coastal wetlands, E: Sand, shingle or pebble shores, F: Estuarine waters, Inland wetlands, M: Permanent rivers/ streams/ creeks, O: Permanent freshwater lakes, P: Seasonal/ intermittent freshwater lakes, Tp: Permanent freshwater marshes/ pools,	27	15	0	E	80
Water	Regularly flooded shrub and/or herbaceous cover	Coastal Wetland		27	15	5	0	80
Water	Regularly flooded shrub and/or herbaceous cover	Coastal Wetland	Human-made wetlands, 9: Cannals and drainage channels, ditches	27	15	5	9	80
Water	Regularly flooded shrub and/or herbaceous cover	Coastal Wetland	Marine or coastal wetlands, E: Sand, shingle or pebble shores, F: Estuarine waters, Inland wetlands, M: Permanent rivers/ streams/ creeks, O: Permanent freshwater lakes, P: Seasonal/ intermittent freshwater lakes, Tp: Permanent freshwater marshes/ pools,	27	15	5	E	80

Water	Cultivated and managed areas			27	16	0	0	101
Water	Cultivated and managed areas		Human-made wetlands, 1: Aquaculture ponds, 2: Ponds, 9: Canals and drainage channels or ditches, 4: Seasonally flooded agricultural land, Inland wetlands, O: Permanent freshwater lakes, Tp: Permanent freshwater marshes/ pools, M: Permanent rivers/ stream	27	16	0	1	78
Water	Cultivated and managed areas		Human-made wetlands, 2: Ponds	27	16	0	2	78
Water	Cultivated and managed areas		Human-made wetlands, 4: Seasonally flooded agricultural land, 6: Water storage areas/Reservoirs, Inland wetlands, M: Permanent rivers/ streams/ creeks, Tp: Permanent freshwater marshes/ pools, Ts: Seasonal/ intermittent freshwater marshes/ pools on inorg	27	16	0	4	81
Water	Cultivated and managed areas		Human-made wetlands, 6: Water storage areas	27	16	0	6	78
Water	Cultivated and managed areas		Human-made wetlands, 7: Excavations	27	16	0	7	78

Water	Cultivated and managed areas		Human-made wetlands, 9: Cannals and drainage channels, ditches	27	16	0	9	78
Water	Cultivated and managed areas		Marine or coastal wetlands, E: Sand, shingle or pebble shores, F: Estuarine waters, Inland wetlands, M: Permanent rivers/ streams/ creeks, O: Permanent freshwater lakes, P: Seasonal/ intermittent freshwater lakes, Tp: Permanent freshwater marshes/ pools,	27	16	0	E	80
Water	Cultivated and managed areas		Inland wetlands, L: Permanent inland deltas	27	16	0	L	47
Water	Cultivated and managed areas		Inland wetlands, M: Permanent rivers/streams/creeks	27	16	0	M	47
Water	Cultivated and managed areas		Inland wetlands, R: Seasonal/intermittent saline/brackish/alkaline lakes and flats	27	16	0	R	

Water	Cultivated and managed areas		Inland wetlands, Sp: Permanent saline/ brackish/ alkaline marshes/ pools, Marine or coastal wetlands, K: Coastal freshwater lagoons, J: Coastal brackish / saline lagoons, Human-made wetlands, 9: Canals and drainage channels or ditches	27	16	0	Sp	52
Water	Cultivated and managed areas		Inland wetlands, Tp: Permanent freshwater marshes/ pools	27	16	0	Tp	51
Water	Cultivated and managed areas		Inland wetlands, Ts: Seasonal/ intermittent freshwater marshes/ pools on inorganic soils, M: Permanent rivers/ streams/ creeks, Xf: Freshwater, tree-dominated wetlands, Human-made wetlands, 1: Aquaculture ponds	27	16	0	Ts	53
Water	Cultivated and managed areas		Inland-wetlands, U: Non-forested peatlands	27	16	0	U	55

Water	Cultivated and managed areas		Inland wetlands, W: Shrub-dominated wetlands, Xf: Freshwater, tree-dominated wetlands, Tp: Permanent freshwater marshes/ pools, P: Seasonal/ intermittent freshwater lakes, O: Permanent freshwater lakes	27	16	0	W	60
Water	Cultivated and managed areas		Inland wetlands, Xf: Freshwater, tree-dominated wetlands, M: Permanent rivers/ streams/ creeks, Tp: Permanent freshwater marshes/ pools, U: Permanent Non-forested peatlands, Human-made wetlands, 6: Water storage areas/Reservoirs	27	16	0	Xf	63
Water	Cultivated and managed areas	Freshwater Marsch, Floodplain		27	16	4	0	51
Water	Cultivated and managed areas	Freshwater Marsch, Floodplain	Human-made wetlands, 6: Water storage areas	27	16	4	6	78
Water	Cultivated and managed areas	Freshwater Marsch, Floodplain	Inland wetlands, Tp: Permanent freshwater marshes/pools	27	16	4	Tp	51
Water	Cultivated and managed areas	Coastal Wetland		27	16	5	0	80

Water	Cultivated and managed areas	Coastal Wetland	Marine or coastal wetlands, E: Sand, shingle or pebble shores, F: Estuarine waters, Inland wetlands, M: Permanent rivers/ streams/ creeks, O: Permanent freshwater lakes, P: Seasonal/ intermittent freshwater lakes, Tp: Permanent freshwater marshes/ pools,	27	16	5	E	80
Water	Cultivated and managed areas	Pan, Brakish/Saline Wetland	Inland wetlands, Ss: Seasonal/ intermittent saline/ brackish/ alkaline marshes/ pools, Q: Permanent saline/ brackish/ alkaline lakes, R: Seasonal/ intermittent saline/ brackish/ alkaline lakes and flats, Sp: Permanent saline/ brackish/ alkaline marshes/	27	16	6	Ss	52
Water	Cultivated and managed areas	Pan, Brakish/Saline Wetland	Inland wetlands, W: Shrub-dominated wetlands, Q: Permanent saline/ brackish/ alkaline lakes, Ss: Seasonal/ intermittent saline/ brackish/ alkaline marshes/ pools, Human-made wetlands, 9: Canals and drainage channels or ditches	27	16	6	W	64

Water	Cultivated and managed areas	Intermittent Wetland/Lake		27	16	8	0	43
Water	Mosaic: Cropland / Tree Cover / Other natural vegetation			27	17	0	0	101
Water	Mosaic: Cropland / Tree Cover / Other natural vegetation	Freshwater Marsch, Floodplain		27	17	4	0	51
Water	Mosaic: Cropland / Tree Cover / Other natural vegetation	Coastal Wetland		27	17	5	0	80
Water	Mosaic: Cropland / Tree Cover / Other natural vegetation	Coastal Wetland	Marine or coastal wetlands, E: Sand, shingle or pebble shores, F: Estuarine waters, Inland wetlands, M: Permanent rivers/ streams/ creeks, O: Permanent freshwater lakes, P: Seasonal/ intermittent freshwater lakes, Tp: Permanent freshwater marshes/ pools,	27	17	5	E	80
Water	Mosaic: Cropland / Shrub and/or grass cover			27	18	0	0	101
Water	Water Bodies			27	20	0	0	101

Water	Water Bodies		Human-made wetlands, 1: Aquaculture	27	20	0	1	78
Water	Water Bodies		Human-made wetlands, 4: Seasonally flooded agricultural land, 6: Water storage areas/Reservoirs, Inland wetlands, M: Permanent rivers/ streams/ creeks, Tp: Permanent freshwater marshes/ pools, Ts: Seasonal/ intermittent freshwater marshes/ pools on inorg	27	20	0	4	81
Water	Water Bodies		Human-made wetlands, 7: Excavations	27	20	0	7	78
Water	Water Bodies		Human-made wetlands, 9: Cannals and drainage channels, ditches	27	20	0	9	78
Water	Water Bodies		Marine or coastal wetlands, E: Sand, shingle or pebble shores, F: Estuarine waters, Inland wetlands, M: Permanent rivers/ streams/ creeks, O: Permanent freshwater lakes, P: Seasonal/ intermittent freshwater lakes, Tp: Permanent freshwater marshes/ pools,	27	20	0	E	80
Water	Water Bodies		Inland wetlands, O: Permanent freshwater lakes	27	20	0	O	42

Water	Water Bodies		Inland wetlands, Tp: Permanent freshwater marshes/ pools	27	20	0	Tp	51
Water	Water Bodies		Inland wetlands, Ts: Seasonal/ intermittent freshwater marshes/ pools on inorganic soils, Tp: Permanent freshwater marshes/ pools, O: Permanent freshwater lakes, Human-made wetlands, 1: Aquaculture ponds, 9: Canals and drainage channels or ditches	27	20	0	Ts	53
Water	Water Bodies		Inland wetlands, W: Shrub-dominated wetlands	27	20	0	W	60
Water	Water Bodies		Inland wetlands, Xf: Freshwater, tree-dominated wetlands, M: Permanent rivers/ streams/ creeks, Tp: Permanent freshwater marshes/ pools, U: Permanent Non-forested peatlands, Human-made wetlands, 6: Water storage areas/Reservoirs	27	20	0	Xf	63
Water	Water Bodies	Freshwater Marsch, Floodplain		27	20	4	0	51
Water	Water Bodies	Freshwater Marsch, Floodplain	Inland wetlands, Xf: Freshwater, tree-dominated wetlands	27	20	4	Xf	63

Water	Water Bodies	Coastal Wetland		27	20	5	0	80
Water	Water Bodies	Coastal Wetland	Marine or coastal wetlands, E: Sand, shingle or pebble shores	27	20	5	E	80
Water	Water Bodies	Coastal Wetland	Inland wetlands, Ts: Seasonal/intermittent freshwater marshes/pools on inorganic soils	27	20	5	Ts	53
Water	Water Bodies	Pan, Brakish/Saline Wetland	Inland wetlands, Ss: Seasonal/ intermittent saline/ brackish/ alkaline marshes/ pools, Q: Permanent saline/ brackish/ alkaline lakes, R: Seasonal/ intermittent saline/ brackish/ alkaline lakes and flats, Sp: Permanent saline/ brackish/ alkaline marshes/	27	20	6	Ss	52
Water	Water Bodies	Intermittent Wetland/Lake		27	20	8	0	43
Water	Snow and Ice			27	21	0	0	101
Water	Artificial surfaces and associated areas			27	22	0	0	101
Water	Artificial surfaces and associated areas	Freshwater Marsch, Floodplain		27	22	4	0	51
Water	Artificial surfaces and associated areas	Freshwater Marsch, Floodplain	Inland wetlands, Tp: Permanent freshwater marshes/ pools	27	22	4	Tp	51

Water	Artificial surfaces and associated areas	Coastal Wetland		27	22	5	0	80
Water	Tree Cover, broadleaved, deciduous, closed		Human-made wetlands, 4: Seasonally flooded agricultural land, 7: Excavations, 9: Canals and drainage channels or ditches, Inland wetlands, M: Permanent rivers/ streams/ creeks, O: Permanent freshwater lakes, P: Seasonal/ intermittent freshwater lakes, Ts	27	2	0	4	63
Rice	0-n	0-n	0-n	31	0-n	0-n	0-n	74
Soybean	0-n	0-n	0-n	33	0-n	0-n	0-n	74
Sunflower	0-n	0-n	0-n	34	0-n	0-n	0-n	74
Cotton	0-n	0-n	0-n	35	0-n	0-n	0-n	74
Vegetables	0-n	0-n	0-n	36	0-n	0-n	0-n	74
Fruits & Berries	0-n	0-n	0-n	37	0-n	0-n	0-n	74
Wine	0-n	0-n	0-n	38	0-n	0-n	0-n	74
Olives	0-n	0-n	0-n	39	0-n	0-n	0-n	74
Winter Triticale	0-n	0-n	0-n	40	0-n	0-n	0-n	74
Water	Tree Cover, needle-leaved, evergreen		Human-made wetlands, 9: Cannals and drainage channels, ditches	27	4	0	9	78
