# Supplementary material for: Ecological Sustainability Assessment of Water Distribution for the Maintenance of Ecosystems, their Services and Biodiversity
Source: Environ Manage. 2022 Jun 14;70(2):329–49. doi: 10.1007/s00267-022-01662-3 (PMC9252940; doi:10.1007/s00267-022-01662-3)
Supplement: Supplementary file 3 — ESM 3 [file 267_2022_1662_MOESM3_ESM.pdf]

Environmental Management

Ecological sustainability assessment of water distribution for the maintenance of ecosystems, their services and biodiversity

Anna Schlattmann[1], Felix Neuendorf, Kremena Burkhard, Elisabeth Probst, Estanislao Pujades, Wolfram Mauser, Sabine Attinger, Christina von Haaren
[1]Leibniz University Hannover, Institute of Environmental Planning, Herrenhaeuserstr. 2, 30419 Hannover, Germany, schlattmann@umwelt.uni-hannover.de

Online Resource 3: Crosswalk EWD typology and EUNIs habitat classification with information on water dependence

EWD Code	EWD-Layer	EUNIS Lvl 1*	EUNIS Lvl 2*	EUNIS Lvl 3*	Water dependence** ()= likely water source	Ecosystem group
	Inland surface waters					
41	**Lakes**	C	C1	C1.1-C1.6	**w (aq)**	wetland
42	**Permanent freshwater lakes (>8ha), incl. Floodplain lakes**	C	C1	C1.1-C1.4	**w (aq)**	wetland
43	**Seasonal/Intermittent freshwater lakes (>8ha), incl. Floodplain lakes**	C	C1, C3	C1.6, C3	**w (aq)**	wetland
44	**Permanent saline, brackish or alkaline lakes**	C	C1	C1.5	**w (aq)**	wetland
45	**Seasonal/Intermittent saline, brackish or alkaline lakes and flats**	C	C1	C1.6, C3, C1.5	**w (aq)**	wetland
46	**Rivers**	C	C2	\	**w (aq)**	wetland
47	**Permanent Rivers, streams or creeks, incl. inland delatas**	C	C2	C2.2, C2.3, C2.4, C2.6	**w (aq)**	wetland
48	**Freshwater springs, oases**	C	C2	C2.1	**w (aq)**	wetland
49	**Seasonal/Intermittent Rivers, streams or creek**	C	C2	C2.5	**w (aq)**	wetland
50	**Wetlands (mires, bogs and fens)**	D/E/F	\	\	**w**	wetland

51	**Permanent freshwater marshes, pools, marshes and swamps on inorganic soils; with emergent vegetation water-logged for at least most of the growing season (herb dominated)**	D	D4, D5	\	**w, (GW)**	wetland
52	**Permanent or seasonal wetlands, incl. Pan, Brackish/Saline/alkaline marshes or pools**	D	D6	\	**w, (GW)**	wetland
53	**Seasonal/Intermittent freshwater marshes/pools on inorganic soils, incl. Sloughs, potholes, seasonally flooded meadows, sedge marshes (Herb dominated)**	D/E	D4, D5, (E3, E5)	\	**w, (GW)**	wetland
54	**Alpine wetlans, incl. Alpine meadows, temporary waters from snow melt**	D/E	D1, D2, D3, D4, D5, E4	D4.2, E4	**w, (GW)**	wetland
55	**Wetland, non-forested bogs, fens and mires, incl. Shrub cover**	D/F	D1, D2, D4, D5, F9	\	**w, (GW)**	wetland
	Grasslands					
56	**Natural Grass**	E	E1, E2, E5	E1.1-E1.C, E2.1-E28, E5.1-E5.3	**t-b**	wetland
57	**Wet grasslands and hydrophillous tall-forb stand, degenerated or drained wetlands**	E/F	E3, E5, F4	E3.1-E3.5, E5.4, E5.5, F4.1	**w, (GW/U/S)**	wetland
58	**Alpine grasslands**	E	E4	\	**t-b**	wetland
	Heathland, shrub and tundra					
59	**Shrub Cover, closed-open, evergreen or deciduous**	F	F2-F8	\	**t**	terrestrial/trees
60	**Wetland, regularly flooded with shrub or herbaceous vegetation on organic or inorganic soils**	F	F9	F91-F9.3	**w**	wetland
61	**Tundra wetlands, incl. Tundra pools, temporary waters from snow melt**	F	F1	\	**w**	wetland

	Woodland, forest and other woodland					
**62**	**Deciduous woodland, closed or open**	G	G1	G1.6, G1.7, G1.8, G1.9, G1.A, G1.B, G1.C, (G1.D)	**t-w (predominantly dry)**	terrestrial/trees
**63**	**Regularly/seasonally flooded woodlands and tree-dominated wetlands, incl. Freshwater swamp forests, wooded swamps on inorganic soils, Forested peatlands, peatswamp forests**	G	G1, G3, G4	G1.1, G1.2; G1.3, G3.D, G3.E, (G.1.4, G1.5), G4.1	**w**	wetland
**64**	**Wetland, regularly flooded, saline water with tree cover**	G	G1, G4	G1.1, G1.2, G1.3, G4.1	**w**	wetland
**65**	**Broadleaved evergreen woodland**	G	G2	\	**t-w (predominantly dry)**	terrestrial/trees
**66**	**Needle-leaved/coniferous, evergreen or deciduous woodland**	G	G3	\	**t-w (predominantly dry)**	terrestrial/trees
**68**	**Mixed broadleaved and coniferous woodland**	G	G4	\	**t-w (predominantly dry)**	terrestrial/trees
**70**	**Tree Cover burnt**	G	G5	G5.8	**t**	terrestrial/trees
	Complexes					
**71**	**Mosaic: Tree cover / Other natural vegetation**	G	X09, X10, X13, X14, X15, X16	\	**t-w**	terrestrial/trees
	Other habitats beyond our scope					
**72**	**Alpine mixed**	\	\	\	**t exclude**	terrestrial/trees
**73**	**Built up**	J	keine weitere Zuordnung		**t exclude**	artificial
**74**	**Crop Land**	**I**	keine weitere Zuordnung		**t exclude**	crop land
**75**	**Extensive Grassland**	**I**	keine weitere Zuordnung		**t exclude**	terrestrial/trees
**76**	**Intensive Grassland**	I	keine weitere Zuordnung		**t exclude**	terrestrial/trees

**77**	**Karst and other subterranean hydrological systems, inland**	H	keine weitere Zuordnung	**w exclude**	wetland
**78**	**Artificial water bodies, reservoirs, treatment areas, channels, inc. combined with natural water bodies or wetlands or terrestrial habitats**	J	J5\ keine weitere Zuordnung	**w (anthrop)**	wetland
**79**	**Unvegetated or sparesly vegetated habitats**	H	keine weitere Zuordnung	**t exclude**	unspecified
**80**	**Marine or Coastal mixed landscapes (deltas)**	A	keine weitere Zuordnung	**w (coast)**	wetland
**81**	**Human-made wetlands incl. Agricultural landscapes**			**w (anthrop)**	wetland

Legend:

(shaded)	= ecosystem types excluded from further GDE analysis
Water dependence/ water source	w = wet/wetland; t = dry/terrestrial; t-w = dry to wet; (aq) = aquatic ecosystem; (GW) = groundwater dependent; (U) = flooding; (S) = waterlogged; (anthrop) = anthropogenic ecosystem; (coast) = coastal ecosystem

References:

* EEA (2019): EUNIS habitat classification 2007 (Revised descriptions 2012) amended 2019; available at: https://www.eea.europa.eu/data-and-maps/data/eunis-habitat-classification/habitats/eunis-habitats-complete-with-descriptions.xls.

EC (2013): Interpretation Manual of the European Habitats EUR 28, Eurpean Commission DG Environment; available at: https://ec.europa.eu/environment/nature/legislation/habitatsdirective/

** BfN (2006): Wasserrahmenrichtlinie, wasserabhängige Lebensraumtypen nach Anhang I der FFH-Richtlinie; available at: https://www.bfn.de/themen/natura-2000/management/kooperation-mit-nutzern/grundwassernutzung.html.

Reich, M.; Rode, M.; von Haaren, Christina & Weiß, C. (2012): Regionales Management von Klimafolgen in der Metropolregion Hannover-Braunschweig-Göttingen, Teilprojekt 4 Klimawandel: lokales und regionales Naturschutzmanagement. Anhang zum Schlussbericht. Institut für Umweltplanung, Hannover.
