# Supplementary material for: Ecological Sustainability Assessment of Water Distribution for the Maintenance of Ecosystems, their Services and Biodiversity
Source: Environ Manage. 2022 Jun 14;70(2):329–49. doi: 10.1007/s00267-022-01662-3 (PMC9252940; doi:10.1007/s00267-022-01662-3)
Supplement: Supplementary file 4 — ESM 4 [file 267_2022_1662_MOESM4_ESM.pdf]

Environmental Management

Ecological sustainability assessment of water distribution for the maintenance of ecosystems, their services and biodiversity

Anna Schlattmann[1], Felix Neuendorf, Kremena Burkhard, Elisabeth Probst, Estanislao Pujades, Wolfram Mauser, Sabine Attinger, Christina von Haaren

[1]Leibniz University Hannover, Institute of Environmental Planning, Herrenhaeuserstr. 2, 30419 Hannover, Germany, schlattmann@umwelt.uni-hannover.de

Online Resource 4: Additional maps and figures

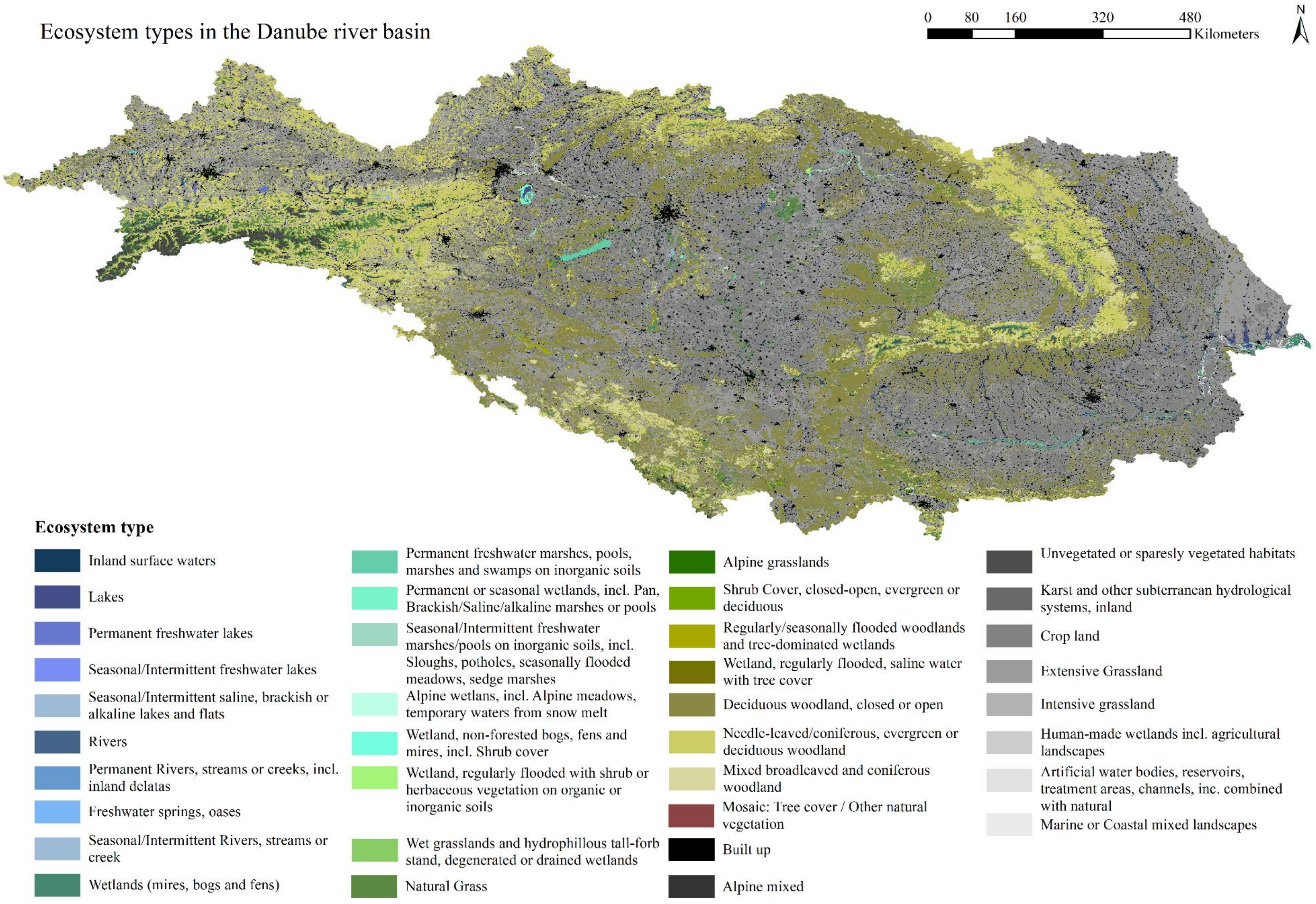

Figure 1: Ecosystem types in the Danube river basin according to the EWD typology.

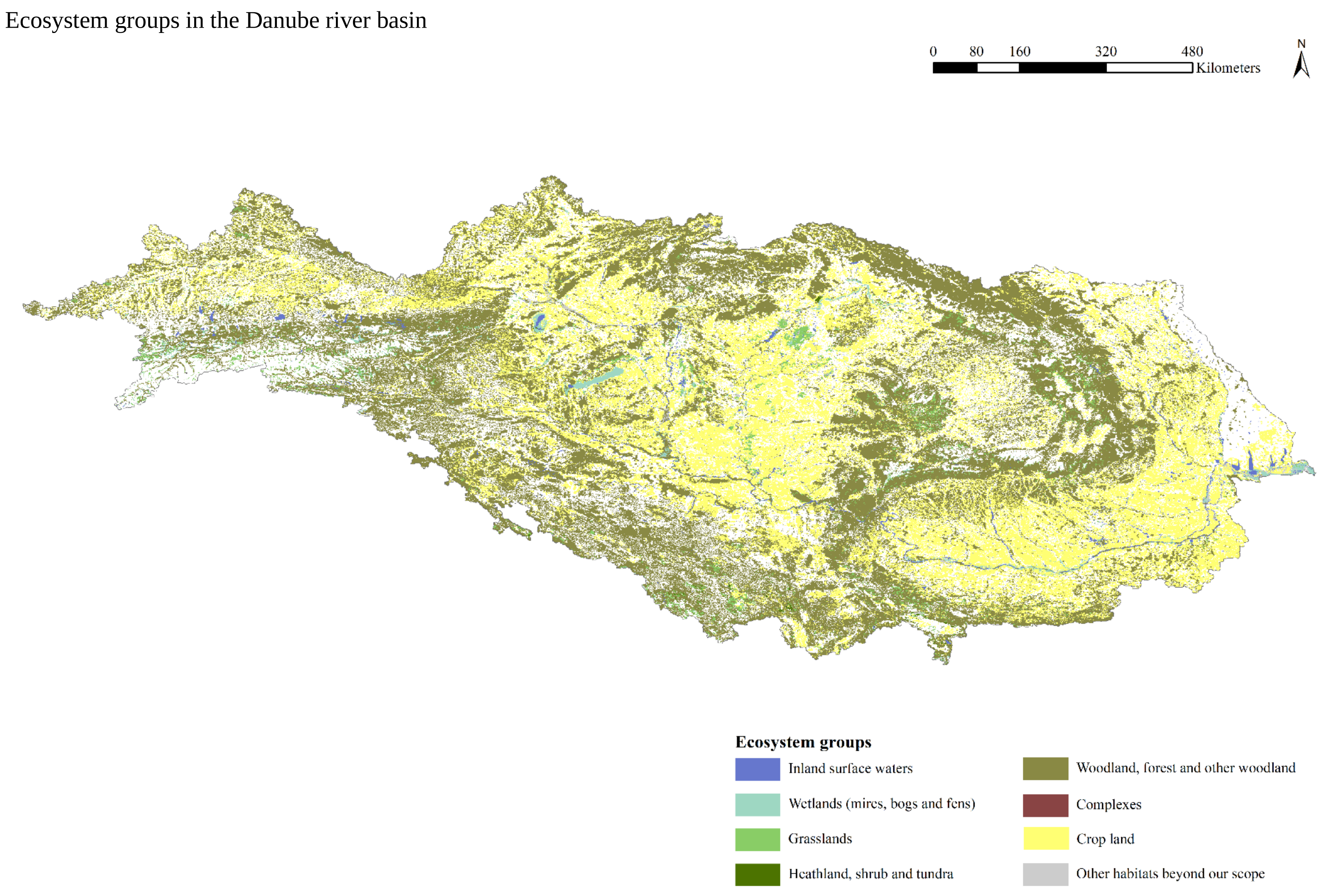

Figure 2: Ecosystem groups in the Danube river basin according to the EWD typology

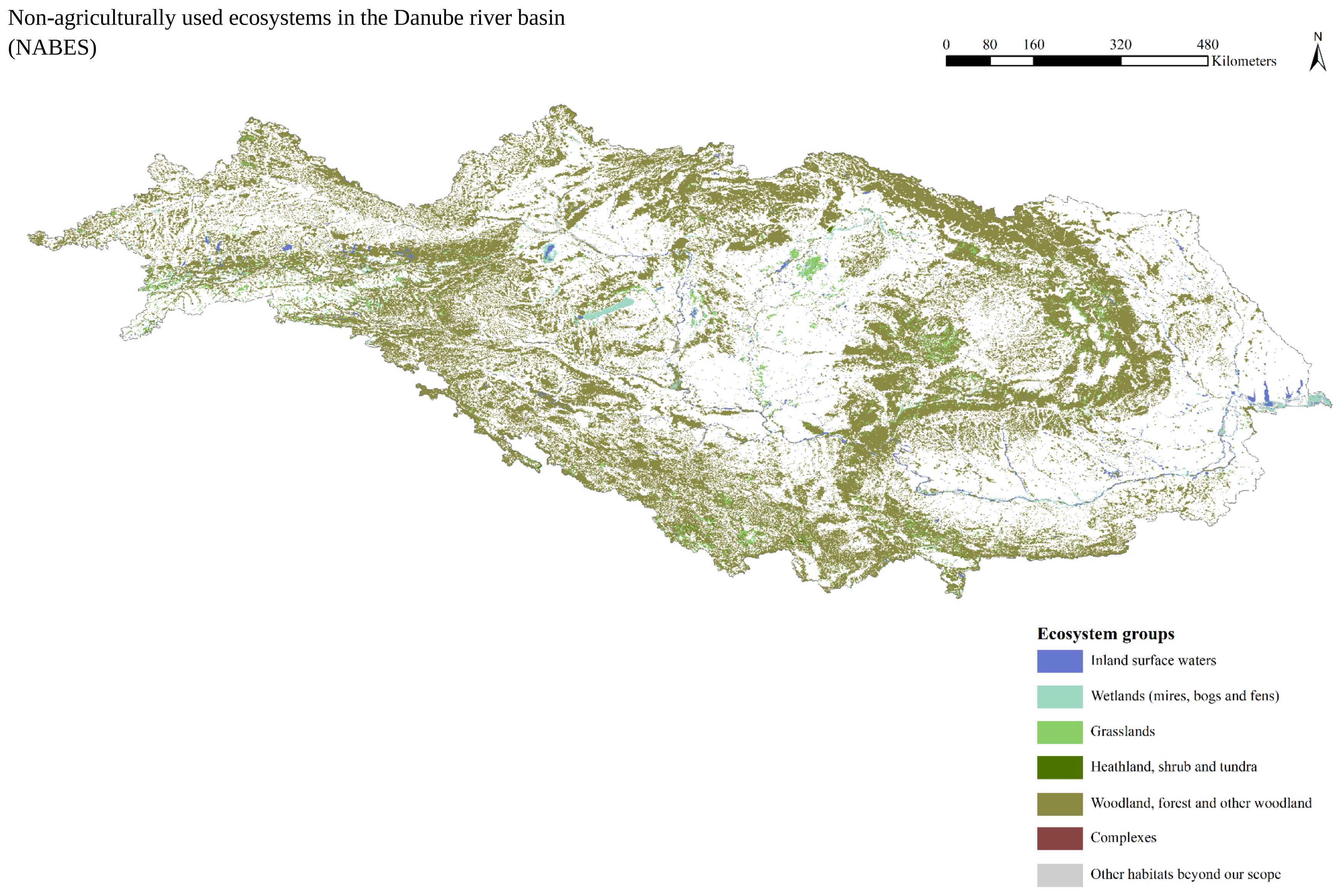

Figure 3: Non-agriculturally used ecosystem groups in the Danube river basin according to the EWD typology

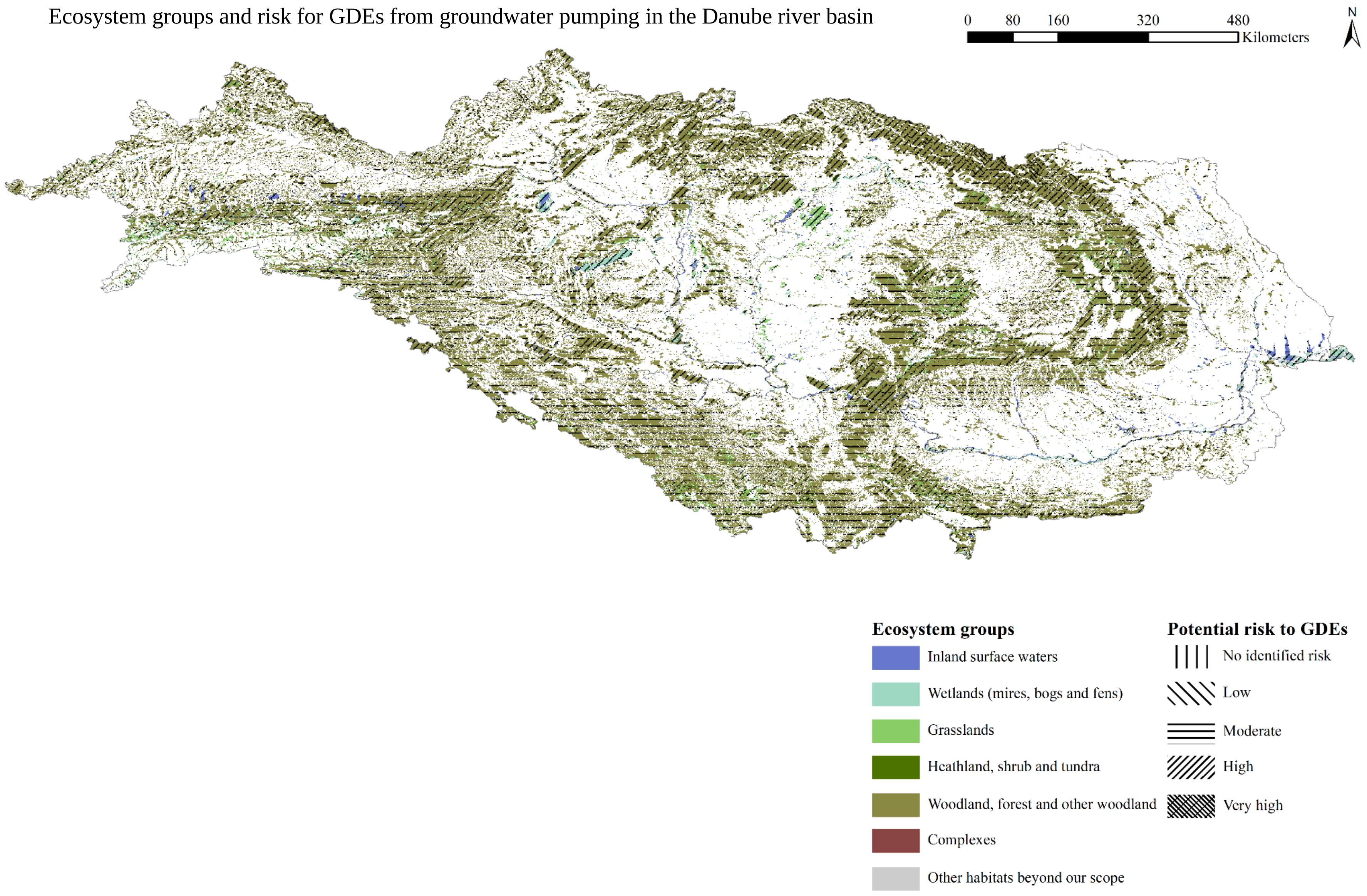

Figure 4: Ecosystem groups in the Danube river basin according to the EWD typology with potential risk for GDEs when groundwater pumping is in place.

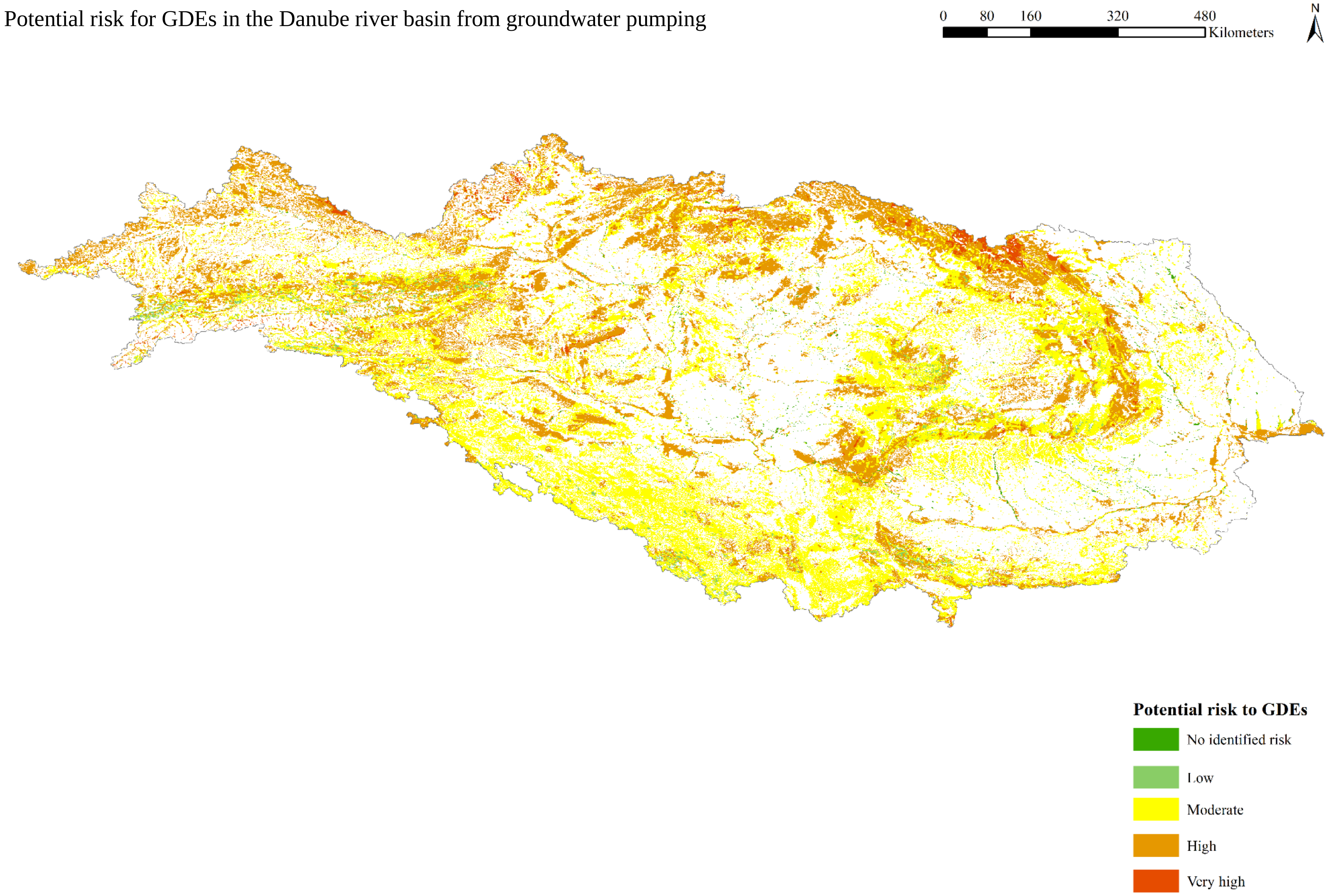

Figure 5: Potential risk for GDEs in the Danube river basin to be impacted when groundwater pumping takes place.
